# International norms for adult handgrip strength: A systematic review of data on 2.4 million adults aged 20 to 100+ years from 69 countries and regions

**DOI:** 10.1016/j.jshs.2024.101014

**Published:** 2024-12-06

**Authors:** Grant R. Tomkinson, Justin J. Lang, Lukáš Rubín, Ryan McGrath, Bethany Gower, Terry Boyle, Marilyn G. Klug, Alexandra J. Mayhew, Henry T. Blake, Francisco B. Ortega, Cristina Cadenas-Sanchez, Costan G. Magnussen, Brooklyn J. Fraser, Tetsuhiro Kidokoro, Yang Liu, Kaare Christensen, Darryl P. Leong, the iGRIPS (international handGRIP Strength) Group, Mette Aadahl, Edimansyah Abdin, Julian Alcazar, Aqeel Alenazi, Bader Alqahtani, Cledir De A. Amaral, Thatiana L.M. Amaral, Alex Andrade Fernandes, Peter Axelsson, Jennifer N. Baldwin, Karin Bammann, Aline R. Barbosa, Ameline Bardo, Inosha Bimali, Peter Bjerregaard, Martin Bobak, Colin A. Boreham, Klaus Bös, João Carlos Bouzas Marins, Joshua Burns, Nadezda Capkova, Lilia Castillo-Martínez, Liang-Kung Chen, Siu Ming Choi, Rebecca K.J. Choong, Susana C. Confortin, Cyrus Cooper, Jorge E. Correa-Bautista, Amandine Cournil, Grace Cruz, Eling D. de Bruin, José Antonio De Paz, Bruno De Souza Moreira, Luiz Antonio Dos Anjos, María Cristina Enríquez Reyna, Eduardo Ferriolli, Gillian Forrester, Elena Frolova, Abadi K. Gebre, Atef M. Ghaleb, Tiffany K. Gill, Yasuyuki Gondo, M. Cristina Gonzalez, Citlali Gonzalez Alvarez, Mary K. Hannah, Nicholas C. Harvey, Jean-Yves Hogrel, Marie-Theres Huemer, Toshiko Iidaka, Lewis A. Ingram, Dmitri A. Jdanov, Victoria L. Keevil, Wolfgang Kemmler, Rose Anne Kenny, Dae-Yeon Kim, Tracy L. Kivell, Ingirid G.H. Kjær, Alexander Kluttig, Rumi Kozakai, Danit Langer, Lisbeth A. Larsen, Wei-Ju Lee, David A. Leon, Eric Lichtenstein, Bertis B. Little, Roberto Alves Lourenço, Rahul Malhotra, Robert M. Malina, Kiyoaki Matsumoto, Tal Mazor-Karsenty, Marnee J. McKay, Sinéad McLoughlin, Abhishek L. Mensegere, Mostafa Mohammadian, Virgilio Garcia Moreira, Hiroshi Murayama, Anne Murray, Anita Liberalesso Neri, Claudia Niessner, Gabriel Núñez Othón, Gabriel Olveira, Suzanne G. Orchard, Andrezj Pajak, Chan Woong Park, Julie A. Pasco, Maria E. Peña Reyes, Leani Souza Máximo Pereira, Annette Peters, Eric Tsz-Chun Poon, Margareth C. Portela, Jedd Pratt, Robinson Ramírez-Vélez, Wendy Rodríguez-García, Joanne Ryan, Mauricio A. San-Martín, Francisco José Sánchez-Torralvo, Mahnaz Saremi, Arno Schmidt-Trucksäss, Satoshi Seino, Shamsul Azhar Shah, Marc Sim, Bjørn Heine Strand, Mythily Subramaniam, Charlotte Suetta, Sophia X. Sui, Jonas S. Sundarakumar, Koya Suzuki, Abdonas Tamosiunas, Maw Pin Tan, Yu Taniguchi, Barbara Thorand, Anna Turusheva, Anne Therese Tveter, Jonathan Wagner, Dao Wang, Stuart J. Warden, Julia Wearing, Shiou Liang Wee, Leo D. Westbury, Agnieszka Wiśniowska-Szurlej, Alexander Woll, Noriko Yoshimura, Ruby Yu

**Affiliations:** 1 Centre for Clinical Research and Prevention, Bispebjerg and Frederiksberg Hospital, Denmark; 2 Department of Clinical Medicine, Faculty of Health and Medical Sciences, University of Copenhagen, Denmark; 3 Research Division, Institute of Mental Health, Singapore; 4 GENUD Toledo Research Group, Faculty of Sports Sciences, University of Castilla-La Mancha, Spain; 5 Department of Geriatric and Palliative Medicine, Copenhagen University Hospital, Bispebjerg and Frederiksberg, Denmark; 6 Centro de Investigación Biomédica en Red Fragilidad y Envejecimiento Saludable (CIBERFES), Instituto de Salud Carlos III, Spain; 7 Grupo Mixto de Fragilidad y Envejecimiento Exitoso UCLM-SESCAM, Universidad de Castilla-La Mancha-Servicio de Salud de Castilla-La Mancha, IDISCAM, Spain; 8 Department of Health and Rehabilitation Sciences, College of Applied Medical Sciences, Prince Sattam Bin Abdulaziz University, Saudi Arabia; 9 Department of Health and Rehabilitation Sciences, College of Applied Medical Sciences, Prince Sattam Bin Abdulaziz University, Saudi Arabia; 10 Federal Institute of Acre, Brazil; 11 Federal University of Acre, Brazil; 12 Instituto Federal de Educação, Ciência e Tecnologia de Minas Gerais—Campus Ipatinga, Brazil; 13 Department of Hand Surgery, Sahlgrenska University Hospital, Sweden; 14 Department of Clinical Sciences, Sahlgrenska Academy, University of Gothenburg, Sweden; 15 Sydney School of Health Sciences, Faculty of Medicine and Health, The University of Sydney, Australia; 16 Institute for Public Health and Nursing Sciences, University of Bremen, Germany; 17 Centro de Desportos, Universidade Federal de Santa Catarina, Brazil; 18 UMR 7194-HNHP, CNRS-MNHN, Département Homme et Environnement, Musée de l'Homme, Paris, France; 19 Department of Human Origins, Max Planck Institute for Evolutionary Anthropology, Germany; 20 Department of Physiotherapy, Kathmandu University School of Medical Sciences, Nepal; 21 National Institute of Public Health, University of Southern Denmark, Denmark; 22 International Institute for Health and Society, Department of Epidemiology and Public Health, University College London, UK; 23 Institute for Sport and Health, University College Dublin, Ireland; 24 Institute of Sports and Sports Science, Karlsruhe Institute of Technology, Germany; 25 Physical Education Department, Federal University of Viçosa, Brazil; 26 Disability Prevention Program, St. Jude Children's Research Hospital, USA; 27 Environmental and Population Health Monitoring Centre, National Institute of Public Health, Czech Republic; 28 Clinical Nutrition Department, Instituto Nacional de Ciencias Médicas y Nutrición Salvador Zubirán, Mexico; 29 Center for Geriatrics and Gerontology, Taipei Veterans General Hospital; 30 Center for Healthy Longevity and Aging Sciences, National Yang Ming Chiao Tung University; 31 Taipei Municipal Gan-Dau Hospital; 32 Faculty of Education, University of Macau, Macao, China; 33 Department of Medicine, Universiti Malaya Medical Centre, Malaysia; 34 Postgraduate Program in Public Health (PPGSCol), University of the Extreme South of Santa Catarina (UNESC), Brazil; 35 MRC Lifecourse Epidemiology Centre, University of Southampton, UK; 36 NIHR Southampton Biomedical Research Centre, University of Southampton and University Hospital Southampton NHS Foundation Trust, UK; 37 NIHR Oxford Biomedical Research Centre, University of Oxford, UK; 38 Facultad de Ciencias del Deporte y la Educación Física, Universidad de Cundinamarca, Colombia; 39 Mission pour la Science Ouverte, Institut de Recherche pour le Développement, France; 40 Population Institute, University of the Philippines, Philippines; 41 Institute of Human Movement Sciences and Sport (IBWS), Department of Health Sciences and Technology, ETH Zurich, Switzerland; 42 OST—Eastern Swiss University of Applied Sciences, Department of Health, Switzerland; 43 Division of Physiotherapy, Department of Neurobiology, Care Sciences and Society, Karolinska Institute, Sweden; 44 Institute of Biomedicine, University of León, Spain; 45 Division of Biological Sciences and Health, University of Sonora, Mexico; 46 Center for Studies in Public Health and Aging—Federal University of Minas Gerais and Oswaldo Cruz Foundation-Minas Gerais, Brazil; 47 Departamento de Nutrição Social, Faculdade de Nutrição Emilia de Jesus Ferreiro, Universidade Federal Fluminense (UFF), Brazil; 48 Universidad Autónoma de Nuevo León, Facultad de Organización Deportiva Monterrey, Mexico; 49 Department of Internal Medicine, Ribeirao Preto Medical School, University of São Paulo, Brazil; 50 School of Psychology, University of Sussex, UK; 51 The North-Western State Medical University named after I.I. Mechnikov, Russia; 52 Nutrition & Health Innovation Research Institute, School of Medical and Health Sciences, Edith Cowan University, Australia; 53 School of Pharmacy, College of Health Sciences, Mekelle University, Mekelle, Ethiopia; 54 Department of Industrial Engineering, College of Engineering, Alfaisal University, Saudi Arabia; 55 Adelaide Medical School, The University of Adelaide, Australia; 56 Alliance for Research in Exercise, Nutrition and Activity (ARENA), Allied Health and Human Performance, University of South Australia, Australia; 57 Graduate School of Human Sciences, Osaka University, Japan; 58 Postgraduate Program in Nutrition and Food, Federal University of Pelotas, Brazil; 59 Pennington Biomedical Research Center, USA; 60 Escuela Nacional de Antropologia e Historia, Instituto Nacional de Antropologia e Historia, Mexico; 61 MRC/CSO Social and Public Health Sciences Unit, School of Health and Wellbeing, University of Glasgow, UK; 62 MRC Lifecourse Epidemiology Centre, University of Southampton, UK; 63 NIHR Southampton Biomedical Research Centre, University of Southampton and University Hospital Southampton NHS Foundation Trust, UK; 64 Neuromuscular Investigation Center, Institute of Myology, France; 65 Institute of Epidemiology, Helmholtz Zentrum München, German Research Center for Environmental Health (GmbH), Germany; 66 Department of Preventive Medicine for Locomotive Organ Disorders, 22^nd^ Century Medical and Research Center, The University of Tokyo, Japan; 67 Alliance for Research in Exercise, Nutrition and Activity (ARENA), Allied Health and Human Performance, University of South Australia, Australia; 68 Max Planck Institute for Demographic Research, Germany; 69 National Research University Higher School of Economics, Russia; 70 Department of Medicine, University of Cambridge, UK; 71 Institute of Radiology, University-Hospital Erlangen, Germany; 72 Institute of Medical Physics, University of Erlangen-Nürnberg, Germany; 73 The Irish Longitudinal Study on Ageing (TILDA), School of Medicine, Trinity College Dublin, Ireland; 74 Measurement and Evaluation in Physical Education and Sport Science, Korea National Sport University, Republic of Korea; 75 Department of Human Origins, Max Planck Institute for Evolutionary Anthropology, Germany; 76 Department of Sport Science and Physical Education, The University of Agder, Norway; 77 Institute of Medical Epidemiology, Biometrics and Informatics, Interdisciplinary Center for Health Sciences, Medical Faculty of the Martin-Luther-University Halle-Wittenberg, Germany; 78 Department of Health and Welfare Science, School of Lifelong Sport, Hokusho University, Japan; 79 Department of Epidemiology of Aging, Research Institute, National Center for Geriatrics and Gerontology, Japan; 80 School of Occupational Therapy, Faculty of Medicine, Hebrew University, Israel; 81 Department of Public Health, Epidemiology, Biostatistics and Biodemography, University of Southern Denmark, Denmark; 82 Center for Healthy Longevity and Aging Sciences, National Yang Ming Chiao Tung University; 83 Department of Family Medicine, Taipei Veterans General Hospital Yuanshan Branch; 84 Faculty of Epidemiology and Population Health, London School of Hygiene & Tropical Medicine, UK; 85 Department of Sport, Exercise and Health, University of Basel, Switzerland; 86 School of Public Health and Information Sciences, University of Louisville, USA; 87 Research Laboratory on Human Aging—GeronLab, Internal Medicine Department, Faculty of Medical Sciences, State University of Rio de Janeiro, Brazil; 88 Department of Medicine, Pontifical Catholic University, Brazil; 89 Centre for Ageing Research and Education, Duke-National University of Singapore Medical School, Singapore; 90 Health Services and Systems Research, Duke-National University of Singapore Medical School, Singapore; 91 Department of Kinesiology and Health Education, University of Texas, USA; 92 School of Public Health and Information Sciences, University of Louisville, USA; 93 Graduate School of Human Sciences, Osaka University, Japan; 94 School of Occupational Therapy, Faculty of Medicine, Hebrew University, Israel; 95 Sydney School of Health Sciences, Faculty of Medicine and Health, The University of Sydney, Australia; 96 The Irish Longitudinal Study on Ageing (TILDA), Trinity Central, Trinity College Dublin, Ireland; 97 Centre for Brain Research, Indian Institute of Science, India; 98 Health Foresight and Innovation Research Center, Institute for Futures Studies in Health, Kerman University of Medical Sciences, Iran; 99 Research Laboratory on Human Aging—GeronLab, Internal Medicine Department, Faculty of Medical Sciences, State University of Rio de Janeiro, Brazil; 100 Tokyo Metropolitan Institute for Geriatrics and Gerontology (TMIG), Japan; 101 Berman Centre for Outcomes and Clinical Research, Hennepin Healthcare Research Institute, USA; 102 University of Minnesota, USA; 103 Department of Educational Psychology, Faculty of Education, State University of Campinas, Brazil; 104 Institute of Sports and Sports Science, Karlsruhe Institute of Technology, Germany; 105 Division of Biological Sciences and Health, University of Sonora, Mexico; 106 Servicio de Endocrinología y Nutrición, Hospital Regional Universitario de Málaga, Spain; 107 IBIMA/plataforma Bionand, Spain; 108 Departamento de Medicina y Dermatología, Universidad de Málaga, Spain; 109 CIBER de Diabetes y Enfermedades Metabólicas Asociadas, Instituto de Salud Carlos III, Spain; 110 School of Public Health and Preventive Medicine, Monash University, Australia; 111 Department of Epidemiology and Population Studies, Jagellonian University Collegium Medicum, Poland; 112 Department of Kinesiology, College of Health & Human Services, Sacramento State University, USA; 113 Deakin University, Institute for Mental and Physical Health and Clinical Translation (IMPACT), Australia; 114 Department of Medicine—Western Health, The University of Melbourne, Australia; 115 Escuela Nacional de Antropologia e Historia, Instituto Nacional de Antropologia e Historia, Mexico; 116 Postgraduate program in Health Sciences at the Faculty of Medical Sciences of Minas Gerais, Brazil; 117 Institute of Epidemiology, Helmholtz Zentrum München, German Research Center for Environmental Health (GmbH), Germany; 118 Institute for Medical Information Processing, Biometry and Epidemiology (IBE), Faculty of Medicine, LMU Munich, Pettenkofer School of Public Health, Germany; 119 Department of Sports Science and Physical Education, The Chinese University of Hong Kong, Hong Kong, China; 120 Sergio Arouca National School of Public Health, Oswaldo Cruz Foundation, Brazil; 121 Institute for Sport and Health, University College Dublin, Ireland; 122 Department of Sport and Exercise Sciences, Manchester Metropolitan University Institute of Sport, UK; 123 Navarrabiomed, Hospital Universitario de Navarra (HUN)-Universidad Pública de Navarra (UPNA), IdiSNA, Spain; 124 CIBER of Frailty and Healthy Aging (CIBERFES), Instituto de Salud Carlos III, Spain; 125 Licenciatura en Nutriología, Facultad de Estudios Superiores Zaragoza, Universidad Nacional Autónoma de México, Mexico; 126 School of Public Health and Preventive Medicine, Monash University, Australia; 127 Locomotor Apparatus and Rehabilitation Institute, Faculty of Medicine, Universidad Austral de Chile, Chile; 128 Servicio de Endocrinología y Nutrición, Hospital Regional Universitario de Málaga, Spain; 129 IBIMA/plataforma Bionand, Spain; 130 Workplace Health Promotion Research Center, School of Public Health and Safety, Shahid Beheshti University of Medical Sciences, Iran; 131 Department of Sport, Exercise and Health, University of Basel, Switzerland; 132 Tokyo Metropolitan Institute for Geriatrics and Gerontology, Japan; 133 Department of Public Health Medicine, Faculty of Medicine, Universiti Kebangsaan Malaysia, Malaysia; 134 Nutrition & Health Innovation Research Institute, School of Medical and Health Sciences, Edith Cowan University, Australia; 135 Medical School, The University Western Australia, Australia; 136 Department of Physical Health and Ageing, Norwegian Institute of Public Health, Norway; 137 Research Division, Institute of Mental Health, Singapore; 138 Saw Swee Hock School of Public Health, National University of Singapore, Singapore; 139 Department of Geriatric and Palliative Medicine, Copenhagen University Hospital, Bispebjerg and Frederiksberg, Denmark; 140 Department of Clinical Medicine, Faculty of Health, University of Copenhagen, Denmark; 141 Deakin University, Institute for Mental and Physical Health and Clinical Translation (IMPACT), Australia; 142 Centre for Brain Research, Indian Institute of Science, India; 143 Graduate School of Health and Sports Science, Juntendo University, Japan; 144 Institute of Cardiology, Medical Academy, Lithuanian University of Health Sciences, Lithuania; 145 Division of Geriatric Medicine, Department of Medicine, Universiti Malaya, Malaysia; 146 Tokyo Metropolitan Institute of Gerontology, Japan; 147 Institute of Epidemiology, Helmholtz Zentrum München, German Research Center for Environmental Health (GmbH), Germany; 148 Institute for Medical Information Processing, Biometry and Epidemiology (IBE), Faculty of Medicine, LMU Munich, Pettenkofer School of Public Health, Germany; 149 The North-Western State Medical University named after I.I. Mechnikov, Russia; 150 Center for treatment of Rheumatic and Musculoskeletal Diseases (REMEDY), Health Service Research and Innovation Unit, Diakonhjemmet Hospital, Norway; 151 Department of Rehabilitation Science and Health Technology, Institute of Health Sciences, Oslo Metropolitan University, Norway; 152 Department of Sport, Exercise and Health, University of Basel, Switzerland; 153 Physical Fitness Research and Health Guidance Center, Shanghai Research Institute of Sports Science (Shanghai Anti-Doping Agency), China; 154 Department of Physical Therapy, School of Health and Human Sciences, Indiana University Indianapolis, USA; 155 School for Interprofessional Health Care, Cooperative State University Baden-Wuerttemberg, Germany; 156 Health and Social Sciences Cluster, Singapore Institute of Technology, Singapore; 157 Geriatric Education and Research Institute, Singapore; 158 MRC Lifecourse Epidemiology Centre, University of Southampton, UK; 159 Institute of Health Sciences, Medical College of Rzeszow University, Poland; 160 Institute of Sports and Sports Science, Karlsruhe Institute of Technology, Germany; 161 Department of Preventive Medicine for Locomotive Organ Disorders, 22^nd^ Century Medical and Research Center, The University of Tokyo, Japan; 162 Department of Medicine and Therapeutics, Faculty of Medicine, The Chinese University of Hong Kong, Hong Kong, China; 163 CUHK Jockey Club Institute of Ageing, The Chinese University of Hong Kong, Hong Kong, China; a Alliance for Research in Exercise, Nutrition and Activity (ARENA), Allied Health and Human Performance, University of South Australia, Adelaide, SA 5000, Australia; b Centre for Surveillance and Applied Research, Public Health Agency of Canada, Ottawa, ON K1A 0K9, Canada; c School of Epidemiology and Public Health, Faculty of Medicine, University of Ottawa, Ottawa, ON K1H 8M5, Canada; d Department of Physical Education and Sport, Faculty of Science, Humanities and Education, Technical University of Liberec, Liberec 461 17, Czech Republic; e Institute of Active Lifestyle, Faculty of Physical Culture, Palacký University Olomouc, Olomouc 779 00, Czech Republic; f Healthy Aging North Dakota (HAND), North Dakota State University, Fargo, ND 58102, USA; g Department of Health, Nutrition and Exercise Sciences, North Dakota State University, Fargo, ND 58108, USA; h Fargo VA Healthcare System, Fargo, ND 58102, USA; i Department of Geriatrics, University of North Dakota, Grand Forks, ND 58202, USA; j Australian Centre for Precision Health, Allied Health and Human Performance, University of South Australia, Adelaide, SA 5000, Australia; k Department of Population Health, University of North Dakota, Grand Forks, ND 58202, USA; l Department of Health Research Methods, Evidence and Impact, Faculty of Health Sciences, McMaster University, Hamilton, ON L8S 4L8, Canada; m Labarge Centre for Mobility in Aging, McMaster University, Hamilton, ON L8P 0A1, Canada; n McMaster Institute for Research on Aging, McMaster University, Hamilton, ON L8P 0A1, Canada; o Department of Physical Education and Sports, Faculty of Sport Sciences, Sport and Health University Research Institute (iMUDS), University of Granada, Granada, ES 18071, Spain; p Centro de Investigación Biomédica en Red Fisiopatología de la Obesidad y Nutrición (CIBERobn), Instituto de Salud Carlos III, Granada, ES 18071, Spain; q Faculty of Sport and Health Sciences, University of Jyväskylä, Jyväskylä 40014, Finland; r Department of Cardiology, Stanford University, Stanford, CA 94305, USA; s Veterans Affairs Palo Alto Health Care System, Palo Alto, CA 94304, USA; t Baker Heart and Diabetes Institute, Melbourne, VIC 3004, Australia; u Research Centre of Applied and Preventive Cardiovascular Medicine, University of Turku, Turku 20520, Finland; v Centre for Population Health Research, University of Turku and Turku University Hospital, Turku 20520, Finland; w Menzies Institute for Medical Research, University of Tasmania, Hobart, TAS 7000, Australia; x Faculty of Sport Science, Nippon Sport Science University, Tokyo 158-8508, Japan; y School of Physical Education, Shanghai University of Sport, Shanghai 200438, China; z Shanghai Research Center for Physical Fitness and Health of Children and Adolescents, Shanghai 200438, China; aa Department of Public Health, Epidemiology Biostatistics and Biodemography, University of Southern Denmark, Odense 5230, Denmark; bb The Population Health Research Institute, McMaster University and Hamilton Health Sciences, Hamilton, ON L8L 2X2, Canada

**Keywords:** Adult, Reference values, Hand strength, Mass screening, Population health

## Abstract

**Background:**

Muscular strength is a powerful marker of current health status and robust predictor of age-related disease and disability. Handgrip strength (HGS) using isometric dynamometry is a convenient, feasible, and widely used method of assessing muscular strength among people of all ages. While adult HGS norms have been published for many countries, no study has yet synthesized available data to produce international norms. The objective of this study was to generate international sex- and age-specific norms for absolute and body size-normalized HGS across the adult lifespan.

**Methods:**

Systematic searches were conducted in 6 databases/web search engines (MEDLINE, SPORTDiscus, Embase, Web of Science, CINAHL, and Google Scholar) up to December 1, 2023. We included full-text peer-reviewed observational studies that reported normative HGS data for adults aged ≥20 years by sex and age. Pseudo data were generated using Monte Carlo simulation following harmonization for methodological variation. Population-weighted Generalized Additive Models for Location, Scale, and Shape were used to develop sex- and age-specific norms for absolute HGS (kg) and HGS normalized by height (Ht, m) squared (i.e., HGS/Ht^2^ in kg/m^2^). Norms were tabulated as percentile values (5th to 95th) and visualized as smoothed percentile curves.

**Results:**

We included data from 100 unique observational studies representing 2,405,863 adults (51.9% female) aged 20 to 100+ years from 69 countries and regions tested from the year 2000 onward. On average, absolute and normalized HGS values negligibly improved throughout early adulthood, peaked from age 30–39 years (at 49.7 kg (males) and 29.7 kg (females) for absolute HGS or 16.3 kg/m^2^ (males) and 11.3 kg/m^2^ (females) for HGS/Ht^2^), and declined afterwards. The age-related decline in HGS accelerated from middle to late adulthood and was slightly larger for males than for females during middle adulthood.

**Conclusion:**

This study provides the world's largest and most geographically comprehensive international norms for adult HGS by sex and age. These norms have utility for global peer-comparisons, health screening, and surveillance.

## Highlights

• Handgrip strength (HGS)—maximal isometric grip force—is an excellent marker of general strength and health.
• Using a systematic review strategy, this study pooled HGS data from 100 unique observational studies representing 2.4 million adults aged 20 to 100+ years from 69 countries and regions tested from the year 2000 onward.
• This study presents the world's largest and most geographically comprehensive international sex- and age-specific norms for HGS across the adult lifespan. Norms for absolute and body size-normalized HGS were tabulated as percentile values and visualized as smoothed percentile curves.
• These norms have utility for global peer-comparisons, health screening, and surveillance.

## Graphical abstract

**Figure:**
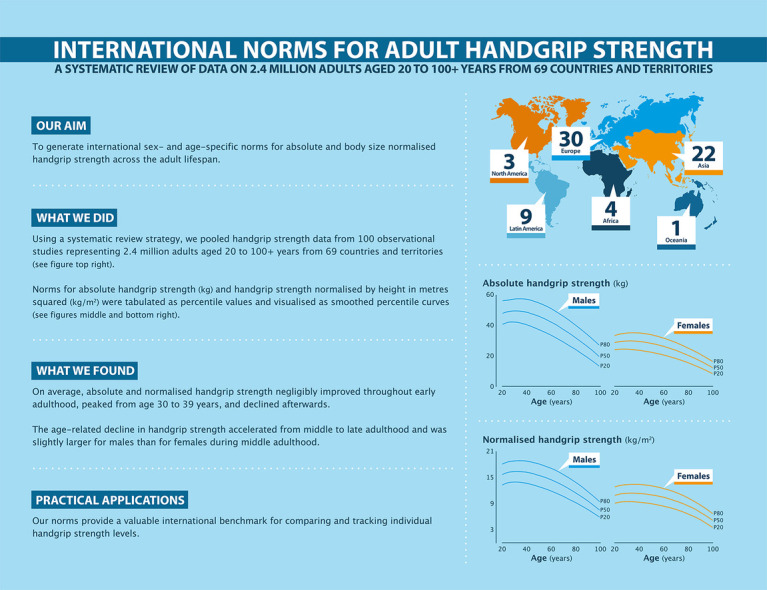

## 1 Introduction

Physical fitness refers to the ability of the bodily systems to work well together to support physical activity and basic self-care. Several components of physical fitness are considered to be health-related because they are strongly and consistently associated with overall health.^1^^,^^2^ One such fitness component is muscular strength, which reflects the ability of a muscle or group of muscles to generate maximal force in a single contraction.^3^ Muscular strength is a powerful marker of current and future health. A recent overview of 8 systematic reviews representing 34 studies and nearly 2 million adults revealed that low muscular strength was significantly linked with early death from all causes and cardiovascular disease as well as a higher incidence of physical disability.^4^ Among adults, low muscular strength better predicts all-cause and cardiovascular mortality than do traditional risk factors like systolic blood pressure.^5^ Low muscular strength is also associated with considerable economic burden on government health care expenditure, with a 10% reduction in the prevalence of adults with low strength at the population level leading to considerable (∼18%) healthcare cost savings.^6^ In their population-based public health guidelines on physical activity and sedentary behavior, the World Health Organization (WHO) recommends muscle-strengthening activities (in addition to aerobic activities) using major muscle groups at a moderate or greater intensity on at least 2 (adults) or 3 (older adults) days a week.^7^ Collectively, this evidence highlights the importance of good muscular strength for mitigating health risks across the lifespan.

Although muscular strength cannot be defined by a single measure, it is widely assessed by handgrip strength (HGS) using handgrip dynamometry, which is easily applicable and recommended for use in clinical, research, and community settings.^8^, ^9^, ^10^ HGS is a convenient, safe, non-invasive, reliable, and feasible method of assessing muscular strength among people of all ages, which can be administered by staff with minimal experience and easily scored and interpreted.^11^^,^^12^ This strength capacity assessment has moderate-to-high construct validity and lower exclusion and dropout rates in epidemiological studies when compared to more complicated assessments of whole-body and major muscle group strength.^13^, ^14^, ^15^ Handgrip dynamometers are also becoming more affordable, with evidence indicating comparable HGS values between lower cost and standard dynamometers.^16^ Measures of HGS have excellent clinical utility^4^^,^^17^^,^^18^ as low HGS is used in decision algorithms and assessment criteria for determining sarcopenia,^19^, ^20^, ^21^ dynapenia,^22^ and frailty.^23^ HGS can also be used as a surveillance tool to monitor temporal trends in population health and to evaluate the effectiveness and monitor the progress of healthy public policies.^24^^,^^25^

A recognized approach to interpret HGS test results is with normative values (herein called norms). Norms allow for comparison to a reference population to determine how well one compares to their peers of the same sex and age. Norms can be used to identify individuals with low muscular strength who may be at risk of poor health and in need of intervention, or individuals with high muscular strength who are likely to perform well in sports or occupational tasks. They can also be used to monitor healthy aging by examining changes in strength capacity over time. Adult HGS norms have been widely published for decades. Such norms generally have been developed using local city or district samples,^26^, ^27^, ^28^, ^29^, ^30^ rather than national samples,^31^, ^32^, ^33^, ^34^, ^35^ and reported for a limited age range (often older adults)^36^, ^37^, ^38^, ^39^, ^40^ rather than across the adult lifespan (i.e., early, middle, and late adulthood).^29^, ^30^, ^31^^,^^33^^,^^35^ Several studies have alternatively pooled HGS data across surveys within^41^, ^42^, ^43^ or among^44^^,^^45^ countries to present norms at the national level^41^, ^42^, ^43^, ^44^ or across diverse geographical regions.^45^ Norms have been reported almost exclusively for absolute HGS, with few studies adjusting for the known influence of body size on strength capacity.^39^, ^40^, ^41^^,^^44^^,^^46^, ^47^, ^48^, ^49^ To our knowledge, no study has systematically pooled published data and reported international norms for both absolute HGS and body size-normalized HGS (herein called normalized HGS) across the adult lifespan.

The primary aim of this study was to develop international sex- and age-specific norms for absolute and normalized HGS across the adult lifespan. We expect these norms to help with the interpretation of HGS test results and to extend the utility of HGS for global peer-comparisons, health screening, and surveillance.

## 2 Methods

### 2.1 Registration and protocol

This systematic review and meta-analysis protocol was prospectively registered with PROSPERO on April 18, 2022 (ID: CRD42022306992). We followed the Preferred Reporting Items for Systematic reviews and Meta-Analyses (PRISMA) 2020 statement (Supplementary Table 1).^50^

### 2.2 Eligibility criteria

Studies were included if they met the following criteria:

a) *Population*: Adults aged ≥20 years at baseline, following the WHO's definition of adult.^51^ We excluded studies on special interest groups (e.g., specific disease, illness, occupation, or athletic groups).
b) *Measure*: Objectively measured HGS in kilograms (kg) (or values from which kg could be calculated) using handgrip dynamometry and a published test protocol. Studies must have tabulated HGS norms in terms of sample size, mean, and SD (or values from which SD could be calculated) stratified by sex, age, and country (for multi-country studies). We included studies that reported closed age groups to a maximum range of 10 years (e.g., 20–29 years). Studies with incomplete information on sampling, test, or normative data reporting protocols were excluded.
c) *Study design*: Unique observational (cross-sectional or cohort) studies reporting HGS data. For cohort studies, data from both baseline (initial) and refreshment (new) samples were included if available, but follow-up data were excluded. We excluded studies that reported duplicate or sub-group data from another included study as well as data from small samples (mean sample size ≤20 across all sex and 5-year age group strata) because mean and SD were less reliable than those from larger studies. Systematic reviews were also excluded.
d) *Publication status*: Full-text peer-reviewed published journal articles. Conference abstracts/papers, commentaries, editorials, and dissertations were excluded.
e) *Timeframe*: To minimize the potential bias of temporal trends and to maintain data recency, only studies reporting HGS data measured from the year 2000 onward (i.e., the midpoint testing year was from the year 2000 onward) were included.

### 2.3 Information sources

We identified studies by searching online databases and other sources, including the reference lists of included studies and topical systematic reviews as recommended by the PRISMA statement.^50^ We followed the recommendations of Bramer and colleagues^52^ regarding the optimal combination of online databases. Searches of databases and web search engines were performed in: MEDLINE (via OVID), SPORTDiscus (via EBSCOhost), Embase (via OVID), Web of Science (Core Collection), CINAHL (via EBSCOhost), and Google Scholar (first 200 results sorted by relevance). All searches were run from database inception to December 1, 2023.

### 2.4 Search strategy

We designed the online search strategy in consultation with an academic librarian experienced in systematic reviews. No language restrictions were applied. The online search strategy is shown in Supplementary Table 2A–2F.

### 2.5 Selection process

Records were imported into EndNote (Clarivate Analytics, Philadelphia, PA, USA), where they were de-duplicated, and then into Covidence (Veritas Health Innovation, Melbourne, VIC, Australia) for further de-duplication and record screening. Titles and abstracts were independently screened against inclusion criteria by 2 authors (GRT and LR) well-experienced in conducting and publishing systematic reviews. Full-text articles were then independently screened against inclusion criteria by the same authors. A third author (BG) resolved conflicts.

### 2.6 Data collection process and data items

Data were independently extracted by one of the following authors (GRT, BG, or HTB) using a pre-designed Excel spreadsheet (Microsoft, Redmond, WA, USA) and were checked for accuracy by another author (LR). The following data were extracted from each study: name of lead author, country or region of participants’ residence, study design (cross-sectional or cohort), sampling strategy (probability (i.e., random selection) or non-probability (i.e., non-random selection)), sample base (national or non-national (i.e., state/provincial or city/district) sampling), cohort/survey name, additional sampling notes, testing year(s), sex/gender, age (range, mean, and SD), standing height (measurement units, sample size (*n*), mean, SD, and median), testing protocol, dynamometer (brand, model, type—electronic (i.e., used electronic load cell), hydraulic (i.e., used hydraulic fluid), or mechanical (i.e., used spring mechanism)), body position (seated or standing), shoulder position (abducted or adducted), elbow position (extended or flexed), radioulnar position (neutral, pronated, or supinated), wrist position (neutral or other (i.e., extended or flexed)), handle position (adjusted to hand size or adjusted to a standard position), time (min) between repetitions (reps) on the same hand (<1 or ≥1), verbal support (yes or no), testing hand (left, right, non-dominant, dominant, or both), reps per hand (1, 2, or 3), summary statistic for normative data reporting (average, average of maxima, or maximum), HGS (measurement units, absolute HGS (*n*, mean, SD, and median for each sex and age stratum), body size-normalized HGS (*n*, mean, SD, and median for each sex and age stratum) if reported as absolute HGS in kg divided by height (Ht) in meters squared (m^2^)), additional study-specific notes, and the full citation.

### 2.7 Quality assessment

Study quality was assessed using the standard quality assessment criteria for evaluating primary research papers from a variety of fields tool.^53^ This tool includes a 14-item checklist for assessing quantitative studies by asking about study design, methods, sample size, analytical approach, confounding factors, study conclusions, *etc*. A score of “Yes” = 2, “Partial” = 1, or “No” = 0 was given to each item depending on the degree to which the criteria were met. Items considered not applicable were scored “NA” and excluded from the total score. The overall quality was calculated by summing the scores across relevant items and dividing by the total possible score (i.e., 28 – (number of NA items × 2)). The quality of all studies was assessed by a single author (BG), with 10% of randomly selected studies independently assessed by a second author (LR).

### 2.8 Synthesis methods and data analyses

We emailed the corresponding authors of each study to ask whether they could help develop international sex- and age-specific norms for adult HGS. To reduce data heterogeneity among studies, we asked study authors to clarify study details (e.g., sampling, test, and reporting protocol) and to either share deidentified raw data or to recalculate descriptive HGS data (weighted according to study-specific protocols) as per our “reference” test and reporting protocol where possible. Public-use raw data were also sought. Ultimately, we had access to descriptive HGS data that were either extracted from published studies or were recalculated from raw data. We reported the descriptive characteristics of included studies as frequencies (percentage) for categorical variables and medians (interquartile range [IQR]) for continuous variables.

To combine data across studies, we harmonized for methodological variation by adjusting HGS values to a common metric, test, and reporting protocol (see Supplementary Harmonization Methods and Supplementary Table 3A–3E for a detailed description). First, we expressed age (years at baseline) in closed 5-year age groups (e.g., 20–24 years, 25–29 years). Second, we expressed absolute HGS values in kg. According to published studies,^46^^,^^54^^,^^55^ we normalized HGS in kg by dividing by height in meters squared (i.e., HGS/Ht^2^ in kg/m^2^) because this is the most appropriate single body size dimension associated with HGS as identified by allometry. Third, we adjusted HGS values to a reference test protocol using Poisson regression models generated from available raw data (*n* = 366,367) to estimate the relative difference between the reference handgrip dynamometer and other dynamometer types, and between reference testing positions and other positions. Lastly, HGS values were adjusted to a reference reporting protocol using within-participant raw data (*n* = 69,528) to estimate the adjustment factors for testing hand, the number of reps per hand, and summary statistic.

We used Monte Carlo simulation to generate pseudo data to develop the international norms for adult HGS. Monte Carlo simulation uses a random number generator to produce approximate normal distributions based on reported mean and SD. Visual analysis of available raw data indicated that both absolute and normalized HGS were normally distributed (Supplementary Fig. 1A–1D). We then cleaned the pseudo datasets with 2 approaches. First, we excluded improbable values based on available raw data (0–100 kg for absolute HGS and 0–35 kg/m^2^ for normalized HGS). Second, we excluded outliers, which we identified as ±3 SD away from the sex- and age-specific mean values. This cleaning process resulted in the exclusion of *n* = 10,368 test results for absolute HGS and *n* = 11,424 test results for normalized HGS.

Norms were developed in *R* (R Foundation for Statistical Computing, Vienna, Austria) using the Generalized Additive Model for Location, Scale, and Shape (GAMLSS) package.^56^^,^^57^ We fitted 50 response distributions and 3 nonparametric smoothing functions (i.e., cubic splines, polynomial splines, and fractional polynomials). All models were stratified by sex. Each GAMLSS model was weighted using United Nations sex- and age-specific population estimates for 2021^58^ to adjust for underlying country-sex-age demographics. Detrended Q-Q (worm) plots were used for visual analysis. We selected the model that provided the best balance between fit and model complexity (i.e., degree of smoothing) using the Akaike Information Criterion. The sinh-arcsinh SHASH (*μ, σ, ν, τ*) distribution with fractional polynomials produced the best balance between fit and smoothness for most models. This distribution is a 4-parameter distribution that includes *μ* (approximately the median, which controls the location), *σ* (approximately the coefficient of variation, which controls the scale), *ν* (approximately the skewness, which controls the asymmetry), and *τ* (approximately the kurtosis). The 5th, 10th, 20th, 30th, 40th, 50th, 60th, 70th, 80th, 90th, and 95th percentiles were calculated, with mean percentile values used to summarize 16 closed age groups, each with a range of 5 years, between the ages of 20 and 99 years (20–24 years…95–99 years) as well as 1 open-ended age group (100+ years). Norms were tabulated as percentile values and visualized as smoothed percentile curves.

### 2.9 Deviation from registered protocol

We planned to use an alternate quality assessment tool but found the standard quality assessment criteria for evaluating primary research papers from a variety of fields tool^53^ more suitable for use with both cross-sectional and cohort studies. While no date restriction was initially planned for our search, we only included studies reporting HGS data measured from the year 2000 onward to minimize the potential bias of temporal trends and to maintain data recency.

## 3 Results

### 3.1 Study selection

**Fig. 1:**
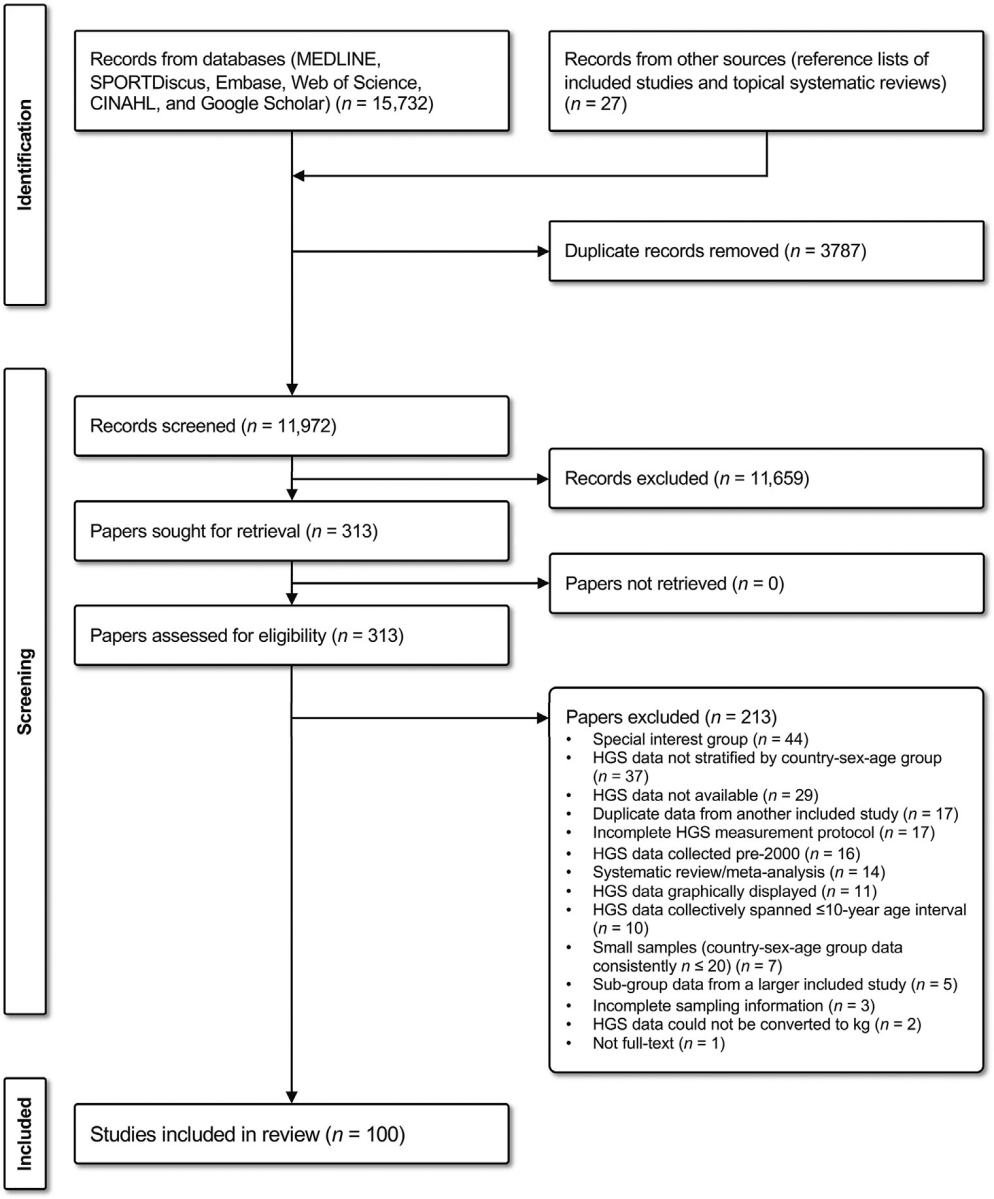
PRISMA flow diagram showing the flow of studies through different phases of the systematic review. HGS = handgrip strength; PRISMA = Preferred Reporting Items for Systematic reviews and Meta-Analyses.

Fig. 1 provides a detailed flow diagram of the literature search and screening process, including reasons for full-text exclusion. A total of 15,732 records were identified from the online database search and 27 additional records were identified from other sources (e.g., reference lists of included studies and topical systematic reviews). After removing 3787 duplicates, 11,972 records were screened at the title/abstract level, which was reduced to 313 papers for full-text screening. Of these, 213 papers were excluded for not meeting inclusion criteria, with 100 unique observational studies included in this review.

### 3.2 Study characteristics

A summary of the descriptive characteristics and HGS test protocols of the included studies is shown in Table 1, with the full list of included studies provided in Supplementary Table 4A and 4B. Following data cleaning, absolute HGS (*n* = 2,405,863) and normalized HGS (*n* = 2,328,890) data were available for adults (51.9% female) aged 20 to 100+ years from 69 countries and regions tested since the year 2000. Because the normalized HGS dataset (generated from 89 studies and 4362 study-country-sex-age groups) was a large subset of the absolute HGS dataset (generated from 100 studies and 4621 study-country-sex-age groups), only the absolute HGS dataset is described herein.

**Table 1:** Summary of the descriptive characteristics and handgrip strength test protocols of the included studies.

Characteristic	Absolute handgrip strength (*n* = 100)	Normalized handgrip strength (*n* = 89)
**Year of testing**		
2000–2009	31 (31.0)	29 (32.6)
2010–2021	69 (69.0)	60 (67.4)
**Stage of adult lifespan**		
Early (20–39 years)	55 (55.0)	46 (51.7)
Middle (40–64 years)	85 (85.0)	74 (83.1)
Late (65+ years)	92 (92.0)	83 (93.3)
All adult stages	47 (47.0)	40 (44.9)
**Sample size**	1690 (618–8357)	1780 (818–8889)
**Study design**		
Cross-sectional	62 (62.0)	52 (58.4)
Cohort	38 (38.0)	37 (41.6)
**Sampling strategy**		
Probability	61 (61.0)	60 (67.4)
Non-probability	37 (37.0)	27 (30.3)
Both	2 (2.0)	2 (2.2)
**Sample base**		
National	36 (36.0)	33 (37.1)
Non-national	64 (64.0)	56 (62.9)
**Dynamometer**		
Hydraulic	38 (38.0)	30 (33.7)
Electronic	32 (32.0)	29 (32.6)
Mechanical	30 (30.0)	30 (33.7)
**Body position**		
Seated	51 (51.0)	41 (46.1)
Standing	49 (49.0)	48 (53.9)
**Shoulder position**		
Adducted	97 (97.0)	86 (96.6)
Abducted (slight, <45°)	3 (3.0)	3 (3.4)
**Elbow position**		
Flexed	68 (68.0)	58 (65.2)
Extended	32 (32.0)	31 (34.8)
**Radioulnar position**		
Neutral	98 (98.0)	87 (97.8)
Supinated	2 (2.0)	2 (2.2)
**Wrist position**		
Neutral	100 (100)	89 (100)
**Handle position**		
Adjusted to hand size	69 (69.0)	66 (74.2)
Adjusted to standard position	31 (31.0)	23 (25.8)
**Time (min) between reps**		
<1	38 (38.0)	34 (38.2)
≥1	33 (33.0)	28 (31.5)
Not specified	29 (29.0)	27 (30.3)
**Verbal support**		
Yes	67 (67.0)	59 (66.3)
No	15 (15.0)	14 (15.7)
Not specified	18 (18.0)	16 (18.0)
**Testing hand**		
Both	73 (73.0)	63 (70.8)
Dominant	26 (26.0)	25 (28.1)
Non-dominant	1 (1.0)	1 (1.1)
**Reps per hand**		
1	6 (6.0)	4 (4.5)
2	42 (42.0)	40 (44.9)
3	52 (52.0)	45 (50.6)
**Summary statistic**		
Maximum	90 (90.0)	83 (93.3)
Average	9 (9.0)	5 (5.6)
Average of maxima	1 (1.0)	1 (1.1)

Notes*:* Data are presented as *n* (%) or median (IQR). Year of testing was calculated as the mid-year of the testing period. At least 5-years’ worth of data had to be available to count for the corresponding stage of adult lifespan. Sample sizes were extracted from published reports or were recalculated from raw data. Additional information can be found in Supplementary Notes and Abbreviations.

Abbreviations: IQR = interquartile range; reps = repetitions.

Normative data were from 47 very high, 13 high, 7 medium, and 2 low human development index countries and regions,^59^ which represented 6 of 7 continents, 17 of 22 United Nations’ geographical subregions,^60^ 71% of the world's population,^58^ and 67% of the world's land area.^61^ Studies presented data for a broad range of ages spanning a median of 34 years (IQR: 30–55), including early (20–39 years), middle (40–64 years), and late adulthood (65+ years). About half (47%) of the studies, represented all stages of the adult lifespan, and nearly all (92%) studies represented late adulthood. The median study-specific sample size was 1690 (IQR: 618–8357), and participant age was 51 years (IQR: 37–62). Most studies collected normative HGS data after the year 2010 (69%), using a cross-sectional study design (62%), probability sampling (61%), and a non-national sample (64%).

In terms of the test protocol, most studies used a hand size-adjusted (69%) hydraulic dynamometer (38%) and measured participants’ HGS while seated (51%) with their testing arm adducted (97%), elbow flexed (68%), and their forearm (98%) and wrist (100%) in a neutral position. Studies generally had participants maximally squeeze the dynamometer 3 times (52%) for each hand (73%), allowed less than 1 min of rest between reps (38%), provided verbal support (67%), and statistically summarized HGS as the maximum value (90%).

### 3.3 Quality assessment

The quality assessments are summarized in Supplementary Table 5. The 2 reviewers (BG and LR) agreed on 94% of item scores, demonstrating nearly perfect inter-rater agreement (*κ* (95% confidence interval): 0.93 (0.88–0.98)). Four Items (#5 (random allocation), #6 (interventional and investigator blinding), #7 (interventional and participant blinding), and #12 (controlled for confounding)) were considered not applicable and excluded from the total score. The median study score was 18 (IQR: 17–19) out of a total possible score of 20, which indicated high quality overall. Common deficiencies were related to partial description of subject selection (Item #3) and an incomplete description of the HGS test protocol (Item #8).

### 3.4 Synthesis of results

**Fig. 2:**
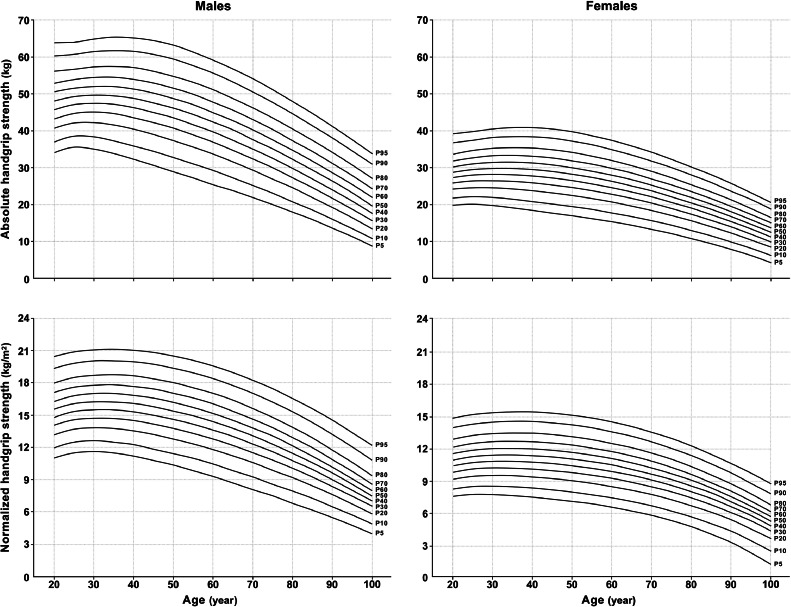
Smoothed percentile curves (P_5_ to P_95_) for absolute handgrip strength in kilograms (kg) and normalized handgrip strength (handgrip strength in kg divided by height in meters squared (kg/m^2^)) for adults aged 20 to 100+ years. Smoothed percentile curves for absolute handgrip strength (top panels) and normalized handgrip strength (bottom panels) are shown separately for males (left panels) and females (right panels). Population-weighted smoothed percentiles were calculated using the Generalized Additive Model for Location, Scale, and Shape method. Percentiles were adjusted to the reference test and reporting protocol (i.e., dynamometer type = hydraulic, body position = seated, elbow position = flexed, radioulnar position = neutral, handle position = adjusted to hand size, testing hand = both, repetitions per hand = 3, and summary statistic = maximum). No statistical adjustment was made for shoulder or wrist position (see Supplementary Harmonization Methods for details). Large, high-resolution images for each panel are provided as Supplementary Fig. 2A–2D.

Tables 2 and 3 show the norms with adjustments for test and reporting protocols as tabulated percentiles (5th to 95th) for absolute and normalized HGS, respectively. Smoothed percentile curves are presented in Fig. 2.

**Table 2:** Normative values (percentiles) for absolute handgrip strength in kilograms by sex and age based on data from 2,405,863 adults aged 20 to 100+ years representing 69 countries and regions.

Age (year)	P_5_	P_10_	P_20_	P_30_	P_40_	P_50_	P_60_	P_70_	P_80_	P_90_	P_95_
**Male**											
20–24	33.9	36.8	40.5	43.2	45.7	48.0	50.4	52.9	56.0	60.1	63.6
25–29	35.5	38.5	42.1	44.8	47.1	49.3	51.5	53.9	56.7	60.7	64.0
30–34	35.0	38.3	42.2	45.0	47.4	49.7	52.0	54.4	57.4	61.5	64.9
35–39	33.8	37.3	41.5	44.5	47.1	49.5	51.9	54.4	57.5	61.8	65.3
40–44	32.3	36.0	40.4	43.6	46.3	48.8	51.2	53.9	57.1	61.5	65.1
45–49	30.6	34.4	39.0	42.3	45.1	47.6	50.2	52.9	56.2	60.7	64.4
50–54	28.9	32.8	37.4	40.7	43.5	46.2	48.8	51.6	54.8	59.4	63.1
55–59	27.2	31.0	35.6	38.9	41.7	44.4	47.0	49.8	53.1	57.7	61.4
60–64	25.5	29.1	33.6	36.9	39.7	42.4	45.0	47.8	51.1	55.6	59.3
65–69	23.7	27.2	31.5	34.7	37.5	40.1	42.8	45.6	48.8	53.2	56.8
70–74	21.9	25.2	29.3	32.4	35.1	37.7	40.3	43.1	46.3	50.6	54.1
75–79	20.0	23.1	27.0	29.9	32.5	35.1	37.6	40.3	43.5	47.7	51.1
80–84	18.0	20.8	24.5	27.3	29.8	32.3	34.8	37.5	40.5	44.7	48.0
85–89	15.9	18.5	21.9	24.6	27.0	29.4	31.8	34.4	37.4	41.5	44.6
90–94	13.7	16.1	19.2	21.7	24.0	26.3	28.7	31.2	34.2	38.1	41.2
95–99	11.3	13.5	16.4	18.8	20.9	23.1	25.4	27.9	30.8	34.6	37.5
100+	8.8	10.8	13.5	15.7	17.8	19.8	22.0	24.5	27.2	30.9	33.8
**Female**											
20–24	19.7	21.7	24.0	25.7	27.2	28.6	30.0	31.6	33.6	36.6	39.1
25–29	20.0	22.0	24.5	26.3	27.9	29.4	30.9	32.6	34.6	37.4	39.7
30–34	19.6	21.8	24.4	26.4	28.1	29.7	31.3	33.1	35.2	38.0	40.4
35–39	19.0	21.3	24.1	26.2	28.0	29.7	31.4	33.2	35.4	38.4	40.8
40–44	18.3	20.7	23.7	25.8	27.6	29.4	31.1	33.0	35.2	38.3	40.8
45–49	17.6	20.1	23.1	25.2	27.1	28.9	30.6	32.5	34.8	37.9	40.4
50–54	16.9	19.4	22.4	24.5	26.4	28.2	29.9	31.8	34.0	37.1	39.7
55–59	16.1	18.5	21.5	23.7	25.5	27.3	29.0	30.9	33.0	36.1	38.6
60–64	15.2	17.6	20.6	22.7	24.5	26.2	27.9	29.7	31.8	34.9	37.4
65–69	14.3	16.6	19.5	21.6	23.3	25.0	26.6	28.4	30.5	33.4	35.8
70–74	13.2	15.5	18.3	20.3	22.0	23.6	25.2	26.9	28.9	31.8	34.1
75–79	12.0	14.3	17.0	18.9	20.5	22.1	23.6	25.2	27.2	29.9	32.2
80–84	10.7	12.9	15.5	17.4	18.9	20.4	21.9	23.5	25.3	28.0	30.2
85–89	9.3	11.4	13.9	15.7	17.2	18.6	20.0	21.5	23.3	25.9	28.0
90–94	7.8	9.8	12.2	13.9	15.3	16.7	18.0	19.5	21.2	23.6	25.7
95–99	6.1	8.0	10.3	11.9	13.3	14.6	15.9	17.3	18.9	21.2	23.2
100+	4.2	6.1	8.3	9.8	11.2	12.4	13.6	14.9	16.5	18.7	20.6

Notes: Population-weighted smoothed percentiles were calculated using the Generalized Additive Model for Location, Scale, and Shape method. Percentiles were adjusted to the reference test and reporting protocol (i.e., dynamometer type = hydraulic, body position = seated, elbow position = flexed, radioulnar position = neutral, handle position = adjusted to hand size, testing hand = both, repetitions per hand = 3, and summary statistic = maximum). No statistical adjustment was made for shoulder or wrist position (see Supplementary Harmonization Methods for details). The ages shown represent 5-year age groups (e.g., 20–24 = 20.00–24.99).

Abbreviation: P = percentile (e.g., P_5_ = 5th percentile).

**Table 3:** Normative values (percentiles) for normalized handgrip strength (handgrip strength in kilograms divided by height in meters squared) by sex and age based on data from 2,328,890 adults aged 20 to 100+ years representing 69 countries and regions.

Age (year)	P_5_	P_10_	P_20_	P_30_	P_40_	P_50_	P_60_	P_70_	P_80_	P_90_	P_95_
**Male**											
20–24	11.0	12.0	13.2	14.1	14.8	15.6	16.3	17.1	18.0	19.3	20.4
25–29	11.5	12.5	13.7	14.6	15.3	16.1	16.8	17.6	18.5	19.8	20.9
30–34	11.6	12.6	13.8	14.7	15.5	16.3	17.0	17.8	18.7	20.0	21.1
35–39	11.5	12.5	13.8	14.7	15.5	16.2	17.0	17.8	18.8	20.1	21.1
40–44	11.2	12.3	13.6	14.5	15.3	16.1	16.9	17.7	18.6	20.0	21.0
45–49	10.8	11.9	13.2	14.2	15.0	15.8	16.6	17.4	18.4	19.7	20.8
50–54	10.4	11.5	12.8	13.8	14.6	15.4	16.2	17.1	18.0	19.4	20.5
55–59	9.9	11.0	12.4	13.3	14.2	15.0	15.8	16.6	17.6	19.0	20.1
60–64	9.3	10.5	11.8	12.8	13.6	14.4	15.2	16.0	17.0	18.4	19.6
65–69	8.7	9.9	11.2	12.2	13.0	13.8	14.6	15.4	16.4	17.8	19.0
70–74	8.1	9.3	10.6	11.6	12.4	13.1	13.8	14.7	15.6	17.1	18.3
75–79	7.5	8.6	9.9	10.8	11.6	12.3	13.1	13.8	14.8	16.2	17.5
80–84	6.9	8.0	9.2	10.1	10.8	11.5	12.2	13.0	13.9	15.3	16.6
85–89	6.2	7.3	8.5	9.3	10.0	10.6	11.2	12.0	12.9	14.3	15.6
90–94	5.5	6.5	7.7	8.4	9.1	9.6	10.2	10.9	11.8	13.2	14.6
95–99	4.8	5.7	6.8	7.5	8.1	8.6	9.2	9.8	10.7	12.1	13.4
100+	4.0	4.9	5.9	6.5	7.1	7.5	8.0	8.6	9.4	10.8	12.2
**Female**											
20–24	7.4	8.1	9.0	9.7	10.3	10.8	11.4	12.0	12.7	13.8	14.7
25–29	7.6	8.3	9.3	10.0	10.6	11.1	11.7	12.4	13.1	14.2	15.0
30–34	7.6	8.4	9.3	10.1	10.7	11.3	11.9	12.5	13.3	14.4	15.2
35–39	7.5	8.3	9.3	10.0	10.7	11.3	11.9	12.6	13.4	14.4	15.3
40–44	7.3	8.2	9.2	10.0	10.6	11.2	11.9	12.5	13.3	14.4	15.3
45–49	7.2	8.0	9.1	9.8	10.5	11.1	11.7	12.4	13.2	14.3	15.2
50–54	7.0	7.8	8.9	9.6	10.3	10.9	11.5	12.2	13.0	14.1	15.0
55–59	6.7	7.6	8.6	9.4	10.1	10.7	11.3	12.0	12.7	13.8	14.7
60–64	6.4	7.3	8.3	9.1	9.7	10.4	11.0	11.6	12.4	13.5	14.4
65–69	6.1	6.9	8.0	8.7	9.4	10.0	10.6	11.2	12.0	13.0	14.0
70–74	5.7	6.5	7.6	8.3	8.9	9.5	10.1	10.7	11.5	12.5	13.4
75–79	5.2	6.1	7.1	7.8	8.4	9.0	9.5	10.1	10.9	11.9	12.8
80–84	4.6	5.5	6.6	7.3	7.9	8.4	8.9	9.5	10.2	11.2	12.1
85–89	3.9	4.9	5.9	6.6	7.2	7.7	8.2	8.8	9.4	10.5	11.4
90–94	3.1	4.1	5.2	5.9	6.5	6.9	7.4	7.9	8.6	9.6	10.5
95–99	2.2	3.3	4.4	5.1	5.6	6.1	6.5	7.0	7.7	8.7	9.6
100+	1.1	2.3	3.5	4.2	4.7	5.1	5.5	6.0	6.6	7.6	8.6

Notes: Population-weighted smoothed percentiles were calculated using the Generalized Additive Model for Location, Scale, and Shape method. Percentiles were adjusted to the reference test and reporting protocol (i.e., dynamometer type = hydraulic, body position = seated, elbow position = flexed, radioulnar position = neutral, handle position = adjusted to hand size, testing hand = both, repetitions per hand = 3, and summary statistic = maximum). No statistical adjustment was made for shoulder or wrist position (see Supplementary Harmonization Methods for details). The ages shown represent 5-year age groups (e.g., 20–24 = 20.00–24.99).

Abbreviation: P = percentile (e.g., P_5_ = 5th percentile).

**Fig. 3:**
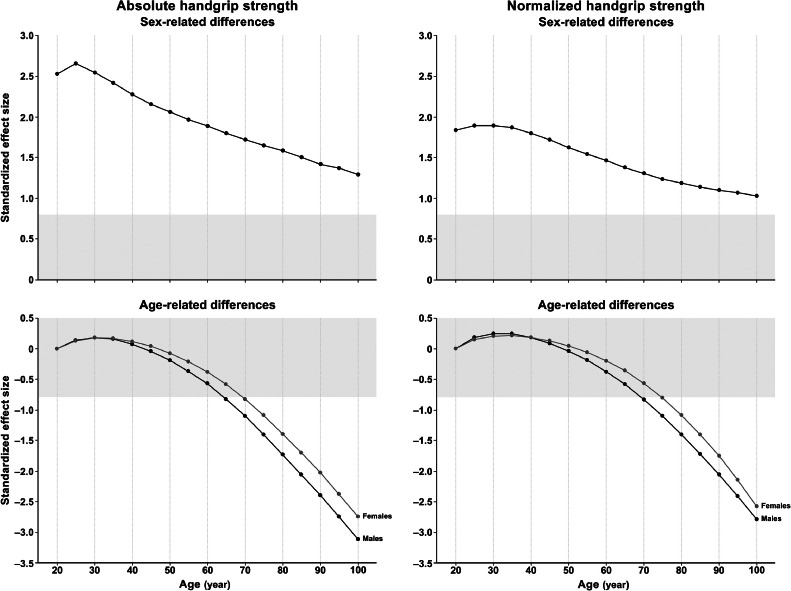
Sex- and age-related differences in mean absolute handgrip strength and normalized handgrip strength for adults aged 20 to 100+ years. Sex-related (i.e., the standardized difference in absolute handgrip strength (top left panel) and normalized handgrip strength (top right panel) between males and females in 5-year age intervals) and age-related differences (i.e., the standardized difference in absolute handgrip strength (bottom left panel) and normalized handgrip strength (bottom right panel) with each 5-year age interval relative to age 20 years) with adjustment for test and reporting protocols are shown as standardized (Cohen's) effect sizes for absolute handgrip strength (left panels) and normalized handgrip strength (right panels). The limits of the gray zone represent a large, standardized effect size (i.e., 0.8 or –0.8). Positive sex-related differences indicate higher handgrip strength for males than for females (top panels). Age-related differences are standardized to age 20 years = 0. Positive age-related differences indicate higher handgrip strength for adults aged >20 years than for adults aged 20 years, and negative age-related differences indicate lower handgrip strength for adults aged >20 years than for adults aged 20 years (bottom panels).

On average, absolute and normalized HGS negligibly improved per decade throughout early adulthood (standardized (Cohen's) effect size (ES) < 0.20, equivalent to 1.0 kg (males) and 0.7 kg (females) for absolute HGS or 0.5 kg/m^2^ (males) and 0.3 kg/m^2^ (females) for HGS/Ht^2^) and peaked from age 30 to 39 years (at 49.7 kg (males) and 29.7 kg (females) or 16.3 kg/m^2^ (males) and 11.3 kg/m^2^ (females)) (Tables 2 and 3, Figs. 2 and 3). HGS declined every decade thereafter, with a negligible-to-small per decade decline in middle adulthood (ES < 0.49, equivalent to 2.8 kg (males) and 1.4 kg (females) or 0.7 kg/m^2^ (males) and 0.4 kg/m^2^ (females)) and a moderate per decade decline in late adulthood (ES: 0.50–0.79, equivalent to 5.6 kg (males) and 3.5 kg (females) or 1.7 kg/m^2^ (males) and 1.3 kg/m^2^ (females)). The age-related decline in HGS was slightly smaller for females than for males during middle adulthood, with age-related changes similar for males and females in early and late adulthood. The sex-related difference in HGS was large (ES ≥ 0.80), though it was 0.77-fold smaller for normalized HGS than for absolute HGS and reduced in magnitude with each decade of adult life.

The variability in HGS was greatest in later life, as evidenced by an age-related increase in relative distributional variability (i.e., the coefficient of variation) especially in late adulthood (data not shown). HGS also varied more for females than for males.

## 4 Discussion

This study presents the most comprehensive international norms for HGS—an important marker of general strength and health—by sex and age across the adult lifespan. Using a systematic review strategy, we pooled HGS data from 100 unique observational studies representing 2.4 million adults aged 20 to 100+ years from 69 countries and regions to present norms for both absolute and normalized HGS. We anticipate that our findings will help support global peer-comparisons, health screening, and surveillance.

These international norms can assist future clinical, research, and surveillance efforts by providing a global benchmark to contextualize adults’ HGS test results relative to their peers of the same sex and age. For instance, our norms can be used to identify individuals who may be at an increased health risk for conditions related to low muscular strength. Cut-points have commonly been used to identify individuals considered weak, as determined by low HGS. One approach is to establish criterion-referenced cut-points, where an individual's HGS level is compared to an absolute (e.g., health- or performance-related) criterion. Criterion-referenced cut-points are often established using receiver-operating characteristic curve or classification regression tree analyses.^19^^,^^62^, ^63^, ^64^, ^65^, ^66^, ^67^ Another approach is to establish norm-referenced cut-points. For example, sarcopenia cut-points for low HGS have been defined as 2.5 SDs below the mean sex-specific value for apparently healthy young adults,^20^ or the lowest sex-specific quintile (i.e., the 20th percentile) value for older adults.^21^ While these cut-points are sex-specific and use younger or older adults as reference groups, they do not integrate age-specific norms into their definition. Alternatively, some authors have proposed a sex- and age-specific quintile framework for use with large datasets to interpret and analyze the distribution of data across the entire adult lifespan.^46^^,^^68^^,^^69^ Such an approach may improve the health- and performance-related predictive utility of HGS for young- and middle-aged adults by better informing screening decisions.^70^

Monitoring age-related changes in strength levels from repeated measurements over time, in addition to estimates of strength levels from a single measurement, may improve the prognostic utility of HGS. For example, several studies^71^^,^^72^ have reported a higher risk of early mortality among older adults who have experienced an accelerated loss of HGS in combination with a low level of HGS. Our norms may be useful for promoting and monitoring healthy aging by allowing age-related changes in strength levels to be tracked against percentile (or quintile) bands to identify expected, better than expected, or worse than expected changes. Consistent with other studies that have examined HGS across the adult lifespan,^29^, ^30^, ^31^^,^^33^^,^^35^ we found that HGS negligibly improved throughout early adulthood, peaked from age 30 to 39 years, and declined thereafter at an increasing rate. However, caution must be taken when using our norms to estimate age-related changes in adult HGS because they may not reflect true within-individual age-related changes.^15^^,^^41^^,^^73^, ^74^, ^75^, ^76^ Furthermore, age-related changes estimated using our norms may be influenced by cohort effects (i.e., generational differences influencing HGS levels).^47^ For example, if younger generations are healthier than older generations,^77^ then the age-related decline in later life experienced by today's young adults may be less than that estimated by our norms. Alternatively, because our inclusion criteria resulted in samples that were likely healthier than the general population, the true age-related decline in later life may be larger than that estimated by our norms.

Our international norms can similarly be used to standardize HGS test results (i.e., to develop *z*-scores) for facilitating comparisons between countries or regions (i.e., comparisons of sex- and age-matched cohorts collected at a similar time), similar to approaches that have been performed elsewhere.^78^ For comparisons within countries (e.g., to identify at-risk populations), scores standardized using our norms may be used as primary or complementary to scores standardized using national norms, such as those presented by Leong and colleagues.^45^ Scores standardized using norms can also be used to estimate temporal trends (i.e., comparisons of sex- and age-matched cohorts collected at different times). Trends in HGS at the population level may correspond to trends in general and functional health and can be used to monitor the progress and evaluate the effectiveness of implemented public health policies.^24^^,^^79^ Web-based monitoring and surveillance systems could likewise be used by investigators to share their data to help update these norms; to provide individuals, clinicians, or sports medicine professionals with a tool to interpret test results and receive informative feedback; and to help public health policy makers with evaluation and decision making by identifying outlying subpopulations or tracking temporal trends. For example, the FitBack platform,^80^ which is a web-based, open-access, multilanguage fitness platform that automatically and interactively interprets fitness test results based on sex- and age-specific norms of children and adolescents and provides advice for improvement, could potentially be expanded to include adults. Such web-based surveillance efforts could complement surveillance efforts that use objective measures. Having robust data infrastructures that enable long-term data pooling and sharing to facilitate regular and comprehensive studies are clearly needed.^81^ We recommend reporting standardized scores using our international norms to facilitate comparisons within and between countries and to monitor temporal trends.

Different HGS test and reporting protocols have been used over time. We found considerable heterogeneity in the protocols of included studies, which differed by dynamometer type and handle positioning, participant positioning, testing hand(s), number of reps and rest, sensory stimuli (e.g., verbal support), and reporting method. Such differences have been highlighted previously^8^^,^^78^ and have made data pooling across studies challenging. For example, our investigation attempted to account for the potential bias introduced by test and reporting differences across studies by calculating and applying adjustment factors (i.e., the risk relative to the reference protocol) ranging from <1% to 10% for different dynamometer types and participant positions, up to 17% for different reporting variants. Given the widespread use of HGS, there is a need to raise awareness of the extant test and reporting inconsistencies and to make recommendations for improving methodological consistency. Such methodological consistency will also have implications for HGS testing in clinical settings as far as helping to reduce misclassification of weakness, specify risk for age-related disease and disability, and guide interventions seeking to improve muscular strength. In support of calls for standardization,^8^^,^^82^ we recommend the following to facilitate future pooling and to improve the utility (and eventual update) of our norms:

a) Ideally, a single and established HGS test protocol should be used (e.g., the Southampton protocol^8^—the reference protocol used to adjust test results in the present study) and an online multilingual operations and procedures manual, including instructional videos, should be made available. At the very least, the test protocol should be accurately described in the main text (or in an online supplement if space is limited due to journal word counts) using the minimum protocol reporting framework provided by McGrath and colleagues;^82^
b) Where possible, 3 reps on each hand should be performed,^83^ with absolute HGS calculated as the maximum value, irrespective of hand, because it better aligns with overall strength capacity;^8^
c) While it might be challenging to adopt a standardized test and reporting protocol, in lieu of that, our adjustment factors should be applied to correct HGS test results to minimize biases when comparing with our norms;
d) Sex- and age-specific descriptive statistics (sample sizes, means, SD, and medians) for HGS should be reported in the main text or Supplementary materials. Where possible, closed age groups should be presented to a range of 5 years. The year(s) of testing should also be reported;
e) To remove the influence of body size and enable the best comparison with our norms, HGS should be normalized to a cross-sectional or surface area measure of body size, such as height-squared, when relevant.^46^^,^^54^^,^^55^

### 4.1 Strengths and limitations

The study pooled data from 100 unique, high-quality observational studies to present international norms for HGS based on data from 2.4 million adults aged 20 to 100+ years from 69 countries and regions. We applied rigorous data treatment procedures to combine datasets and harmonize for methodological variation, and we recreated unavailable raw data to generate absolute and normalized HGS norms using population-weighted GAMLSS that provided the best balance between fit and model complexity.

Despite these strengths, this study is not without limitations. First, included studies used different sampling methods (both probability and non-probability selection) and bases (across national, state/provincial, and city/district levels) and mostly included adults from countries with high to very high human development, who are known to exhibit higher levels of HGS than their peers from countries with low to medium human development.^78^ This obviously raises the issue of representativeness. However, while more adult HGS data are needed from geographical regions where data are few (e.g., Africa (Northern, Eastern, Southern, Western, and Middle Africa), the Caribbean, Central America, and Oceania (Melanesia, Micronesia, and Polynesia)) to improve global representativeness, we included the best available data and applied a poststratification population weighting procedure to adjust for underlying country-sex-age demographics and to better estimate internationally representative population parameters. Second, differences in test and reporting protocols among studies, which are inherent to any large data synthesis, may have biased our results. While our norms were adjusted for test and reporting differences, it is acknowledged that we could not adjust for all test variants. Third, we examined sex as a biological variable by sex-stratifying our analyses, which is consistent with certain scientific guidelines^84^ and most included studies, although some studies reported gender instead of sex. Fourth, HGS testing may have been contraindicated for adults with chronic conditions (disease, injury, or illness) or those presenting with pain, resulting in them being excluded or opting out. While such exclusions differed among studies and were not always reported, it is likely that the included samples were healthier than the general population. The absence of data from these individuals may have meant that our lower percentiles overestimated true general population values. In lieu of a global study examining adult HGS levels across a representative sample of countries using a standardized sampling, test, and reporting protocol, our international norms represent the best estimate of current global adult HGS levels.

### 4.2 Practical applications

Our norms provide a valuable international benchmark with which to compare and track individual HGS test results. We propose a quintile framework to interpret our international sex- and age-specific norms in clinical settings. For example, adults below the 20th percentile can be considered as having “low” strength; between the 20th and 39th percentiles as having “somewhat low” strength; between the 40th and 59th percentiles as having “moderate” strength; between the 60th and 79th percentiles as having “somewhat high” strength; and at or above the 80th percentile as having “high” strength. Comparison to our norms could help clinicians or sports medicine professionals identify adults who may need to improve their strength capacity or who may benefit from further assessment. In prospective cohort studies,^23^^,^^85^, ^86^, ^87^, ^88^, ^89^ the lowest quintile has been used as a threshold for defining low fitness and has been significantly linked with increased risk of poor health or early death in later life.

While there is heterogeneity in the definition of low HGS,^90^ in the absence of universal sex- and age-specific criterion-referenced cut-points for low HGS, interim cut-points corresponding to our lowest quintile could be used to identify at-risk adults until better evidence for criterion-referenced health-related cut-points is established by future research.^91^ Adults classified as having low HGS may “need improvement” and are potentially at increased future health risk if they continue to track at this level. Clinicians or sports medicine professionals can then provide feedback, advice, and intervention referral for improving overall strength capacity. For example, advice may include strategies for helping persons classified as needing improvement in their strength meet the minimum weekly threshold for muscle-strengthening activities as recommended in the WHO's global physical activity guidelines.^7^ Provided that the prescribed muscle-strengthening activities are multimodal,^92^ value-added follow-up HGS assessments could be informative by evaluating the effectiveness of physical activity programming through monitoring of progress against our percentile bands. Future research is needed to empirically validate the health-related predictive utility of our interim cut-points or to provide evidence for universal criterion-referenced health-related cut-points.

We provided sex- and age-specific norms for both absolute and normalized HGS across the adult lifespan. Because absolute HGS is significantly associated with future health outcomes^4^^,^^5^ and can be easily measured and interpreted with minimal data post-processing, clinicians and sports medicine professionals may prefer to simply compare or track individual HGS test results against our absolute HGS norms to provide more timely feedback to individuals. However, in addition to sex and age, body size is known to influence HGS, with body size strongly and positively associated with HGS.^46^^,^^54^ Therefore, when comparing or tracking test results against our absolute HGS norms, larger individuals may be unfairly advantaged (i.e., appear better than they are) and smaller individuals unfairly disadvantaged (i.e., appear worse than they are). To overcome this limitation and remove the confounding influence of body size, HGS should be normalized to a cross-sectional or surface area measure of body size, such as height-squared.^46^ Normalizing HGS to height-squared creates a “level playing field” (i.e., no systematic advantage or disadvantage), providing a fairer way of comparing the HGS of adults who differ in body size. While normalized HGS test results require additional data post-processing and may be more challenging to interpret than absolute HGS test results, we recommend that both absolute and normalized HGS be assessed and compared against our norms for extra insight into how body size influences strength capacity.

## 5 Conclusion

HGS is an excellent marker of general strength and health that is widely used in clinical, research, and community settings. This study pooled, harmonized, and analyzed the best available data to present the world's largest and most geographically comprehensive international norms for HGS by sex and age across the adult lifespan. Our international norms can be used to identify adults with low or high strength relative to their peers of the same sex and age and to monitor healthy aging. These norms should be updated in the future to better reflect the HGS of subsequent generations of adults.

## Authors’ contributions

GRT conceptualized and designed the study, conducted record screening, extracted/acquired and interpreted the data, had full access to the data, led the statistical analysis, and wrote the original draft; JJL conceptualized and designed the study, led the statistical analysis, and edited the manuscript; LR conceptualized and designed the study, conducted record screening, assessed study quality, and edited the manuscript; RM conceptualized and designed the study, and edited the manuscript; BG conducted record screening, extracted/acquired and interpreted the data, assessed study quality, and edited the manuscript; TB and MGK interpreted the data, supported the statistical analysis, and edited the manuscript; AJM extracted/acquired and interpreted the data, supported the statistical analysis, and edited the manuscript; HTB extracted/acquired and interpreted the data, and edited the manuscript; FBO, CC-S, CGM and BJF interpreted the data and edited the manuscript; TK, YL, KC and DPL extracted/acquired and interpreted the data, and edited the manuscript; and iGRIPS group authors extracted/acquired and interpreted the data. All authors have read and approved the final version of the manuscript, and agree with the order of presentation of the authors.

## Competing interests

The authors declare that they have no competing interests.

## Ethical approval information

This study was reviewed by the Human Research Ethics Committee of the University of South Australia and received ethics exemption (HREC ID: 205086).

## Disclaimer

The content and views expressed in this article are those of the authors and do not necessarily reflect those of the Government of Canada.

## Data availability statement

The descriptive aggregate data used in this study are available upon reasonable request and agreement from the first author and relevant data custodians. Although we had access to some de-identified raw data, we are unable to share these datasets without permission from their data custodians.

## Appendix A The names and affiliations of the iGRIPS (international handGRIP Strength) group

Mette Aadahl, Centre for Clinical Research and Prevention, Bispebjerg and Frederiksberg Hospital, Denmark; Department of Clinical Medicine, Faculty of Health and Medical Sciences, University of Copenhagen, Denmark. Edimansyah Abdin, Research Division, Institute of Mental Health, Singapore. Julian Alcazar, GENUD Toledo Research Group, Faculty of Sports Sciences, University of Castilla-La Mancha, Spain; Department of Geriatric and Palliative Medicine, Copenhagen University Hospital, Bispebjerg and Frederiksberg, Denmark; Centro de Investigación Biomédica en Red Fragilidad y Envejecimiento Saludable (CIBERFES), Instituto de Salud Carlos III, Spain; Grupo Mixto de Fragilidad y Envejecimiento Exitoso UCLM-SESCAM, Universidad de Castilla-La Mancha-Servicio de Salud de Castilla-La Mancha, IDISCAM, Spain. Aqeel Alenazi, Department of Health and Rehabilitation Sciences, College of Applied Medical Sciences, Prince Sattam Bin Abdulaziz University, Saudi Arabia. Bader Alqahtani, Department of Health and Rehabilitation Sciences, College of Applied Medical Sciences, Prince Sattam Bin Abdulaziz University, Saudi Arabia. Cledir de A. Amaral, Federal Institute of Acre, Brazil. Thatiana L. M. Amaral, Federal University of Acre, Brazil. Alex Andrade Fernandes, Instituto Federal de Educação, Ciência e Tecnologia de Minas Gerais—Campus Ipatinga, Brazil. Peter Axelsson, Department of Hand Surgery, Sahlgrenska University Hospital, Sweden; Department of Clinical Sciences, Sahlgrenska Academy, University of Gothenburg, Sweden. Jennifer N. Baldwin, Sydney School of Health Sciences, Faculty of Medicine and Health, The University of Sydney, Australia. Karin Bammann, Institute for Public Health and Nursing Sciences, University of Bremen, Germany. Aline R. Barbosa, Centro de Desportos, Universidade Federal de Santa Catarina, Brazil. Ameline Bardo, UMR 7194-HNHP, CNRS-MNHN, Département Homme et Environnement, Musée de l'Homme, France; Department of Human Origins, Max Planck Institute for Evolutionary Anthropology, Germany. Inosha Bimali, Department of Physiotherapy, Kathmandu University School of Medical Sciences, Nepal. Peter Bjerregaard, National Institute of Public Health, University of Southern Denmark, Denmark. Martin Bobak, International Institute for Health and Society, Department of Epidemiology and Public Health, University College London, UK. Colin A. Boreham, Institute for Sport and Health, University College Dublin, Ireland. Klaus Bös, Institute of Sports and Sports Science, Karlsruhe Institute of Technology, Germany. João Carlos Bouzas Marins, Physical Education Department, Federal University of Viçosa, Brazil. Joshua Burns, Disability Prevention Program, St. Jude Children's Research Hospital, USA. Nadezda Capkova, Environmental and Population Health Monitoring Centre, National Institute of Public Health, Czech Republic. Lilia Castillo-Martínez, Clinical Nutrition Department, Instituto Nacional de Ciencias Médicas y Nutrición Salvador Zubirán, Mexico. Liang-Kung Chen, Center for Geriatrics and Gerontology, Taipei Veterans General Hospital; Center for Healthy Longevity and Aging Sciences, National Yang Ming Chiao Tung University; Taipei Municipal Gan-Dau Hospital. Siu Ming Choi, Faculty of Education, University of Macau, Macao, China. Rebecca K. J. Choong, Department of Medicine, Universiti Malaya Medical Centre, Malaysia. Susana C. Confortin, Postgraduate Program in Public Health (PPGSCol), University of the Extreme South of Santa Catarina (UNESC), Brazil. Cyrus Cooper, MRC Lifecourse Epidemiology Centre, University of Southampton, UK; NIHR Southampton Biomedical Research Centre, University of Southampton and University Hospital Southampton NHS Foundation Trust, UK; NIHR Oxford Biomedical Research Centre, University of Oxford, UK. Jorge E. Correa-Bautista, Facultad de Ciencias del Deporte y la Educación Física, Universidad de Cundinamarca, Colombia. Amandine Cournil, Mission pour la Science Ouverte, Institut de Recherche pour le Développement, France. Grace Cruz, Population Institute, University of the Philippines, Philippines. Eling D. de Bruin, Institute of Human Movement Sciences and Sport (IBWS), Department of Health Sciences and Technology, ETH Zurich, Switzerland; OST—Eastern Swiss University of Applied Sciences, Department of Health, Switzerland; Division of Physiotherapy, Department of Neurobiology, Care Sciences and Society, Karolinska Institute, Sweden. José Antonio De Paz, Institute of Biomedicine, University of León, Spain; Division of Biological Sciences and Health, University of Sonora, Mexico. Bruno De Souza Moreira, Center for Studies in Public Health and Aging—Federal University of Minas Gerais and Oswaldo Cruz Foundation-Minas Gerais, Brazil. Luiz Antonio Dos Anjos, Departamento de Nutrição Social, Faculdade de Nutrição Emilia de Jesus Ferreiro, Universidade Federal Fluminense (UFF), Brazil. María Cristina Enríquez Reyna, Universidad Autónoma de Nuevo León, Facultad de Organización Deportiva Monterrey, Mexico. Eduardo Ferriolli, Department of Internal Medicine, Ribeirao Preto Medical School, University of São Paulo, Brazil. Gillian Forrester, School of Psychology, University of Sussex, UK. Elena Frolova, The North-Western State Medical University named after I.I. Mechnikov, Russia. Abadi K. Gebre, Nutrition & Health Innovation Research Institute, School of Medical and Health Sciences, Edith Cowan University, Australia; School of Pharmacy, College of Health Sciences, Mekelle University, Ethiopia. Atef M. Ghaleb, Department of Industrial Engineering, College of Engineering, Alfaisal University, Saudi Arabia. Tiffany K. Gill, Adelaide Medical School, The University of Adelaide, Australia; Alliance for Research in Exercise, Nutrition and Activity (ARENA), Allied Health and Human Performance, University of South Australia, Australia. Yasuyuki Gondo, Graduate School of Human Sciences, Osaka University, Japan. M. Cristina Gonzalez, Postgraduate Program in Nutrition and Food, Federal University of Pelotas, Brazil; Pennington Biomedical Research Center, USA. Citlali Gonzalez Alvarez, Escuela Nacional de Antropologia e Historia, Instituto Nacional de Antropologia e Historia, Mexico. Mary K. Hannah, MRC/CSO Social and Public Health Sciences Unit, School of Health and Wellbeing, University of Glasgow, UK. Nicholas C. Harvey, MRC Lifecourse Epidemiology Centre, University of Southampton, UK; NIHR Southampton Biomedical Research Centre, University of Southampton and University Hospital Southampton NHS Foundation Trust, UK. Jean-Yves Hogrel, Neuromuscular Investigation Center, Institute of Myology, France. Marie-Theres Huemer, Institute of Epidemiology, Helmholtz Zentrum München, German Research Center for Environmental Health (GmbH), Germany. Toshiko Iidaka, Department of Preventive Medicine for Locomotive Organ Disorders, 22^nd^ Century Medical and Research Center, The University of Tokyo, Japan. Lewis A. Ingram, Alliance for Research in Exercise, Nutrition and Activity (ARENA), Allied Health and Human Performance, University of South Australia, Australia. Dmitri A. Jdanov, Max Planck Institute for Demographic Research, Germany; National Research University Higher School of Economics, Russia. Victoria L. Keevil, Department of Medicine, University of Cambridge, UK. Wolfgang Kemmler, Institute of Radiology, University-Hospital Erlangen, Germany; Institute of Medical Physics, University of Erlangen-Nürnberg, Germany. Rose Anne Kenny, The Irish Longitudinal Study on Ageing (TILDA), School of Medicine, Trinity College Dublin, Ireland. Dae-Yeon Kim, Measurement and Evaluation in Physical Education and Sport Science, Korea National Sport University, Republic of Korea. Tracy L. Kivell, Department of Human Origins, Max Planck Institute for Evolutionary Anthropology, Germany. Ingirid G. H. Kjær, Department of Sport Science and Physical Education, The University of Agder, Norway. Alexander Kluttig, Institute of Medical Epidemiology, Biometrics and Informatics, Interdisciplinary Center for Health Sciences, Medical Faculty of the Martin-Luther-University Halle-Wittenberg, Germany. Rumi Kozakai, Department of Health and Welfare Science, School of Lifelong Sport, Hokusho University, Japan; Department of Epidemiology of Aging, Research Institute, National Center for Geriatrics and Gerontology, Japan. Danit Langer, School of Occupational Therapy, Faculty of Medicine, Hebrew University, Israel. Lisbeth A. Larsen, Department of Public Health, Epidemiology, Biostatistics and Biodemography, University of Southern Denmark, Denmark. Wei-Ju Lee, Center for Healthy Longevity and Aging Sciences, National Yang Ming Chiao Tung University; Department of Family Medicine, Taipei Veterans General Hospital Yuanshan Branch. David A. Leon, Faculty of Epidemiology and Population Health, London School of Hygiene & Tropical Medicine, UK. Eric Lichtenstein, Department of Sport, Exercise and Health, University of Basel, Switzerland. Bertis B. Little, School of Public Health and Information Sciences, University of Louisville, USA. Roberto Alves Lourenço, Research Laboratory on Human Aging—GeronLab, Internal Medicine Department, Faculty of Medical Sciences, State University of Rio de Janeiro, Brazil; Department of Medicine, Pontifical Catholic University, Brazil. Rahul Malhotra, Centre for Ageing Research and Education, Duke-National University of Singapore Medical School, Singapore; Health Services and Systems Research, Duke-National University of Singapore Medical School, Singapore. Robert M. Malina, Department of Kinesiology and Health Education, University of Texas, USA; School of Public Health and Information Sciences, University of Louisville, USA. Kiyoaki Matsumoto, Graduate School of Human Sciences, Osaka University, Japan. Tal Mazor-Karsenty, School of Occupational Therapy, Faculty of Medicine, Hebrew University, Israel. Marnee J. McKay, Sydney School of Health Sciences, Faculty of Medicine and Health, The University of Sydney, Australia. Sinéad McLoughlin, The Irish Longitudinal Study on Ageing (TILDA), Trinity Central, Trinity College Dublin, Ireland. Abhishek L. Mensegere, Centre for Brain Research, Indian Institute of Science, India. Mostafa Mohammadian, Health Foresight and Innovation Research Center, Institute for Futures Studies in Health, Kerman University of Medical Sciences, Iran. Virgilio Garcia Moreira, Research Laboratory on Human Aging—GeronLab, Internal Medicine Department, Faculty of Medical Sciences, State University of Rio de Janeiro, Brazil. Hiroshi Murayama, Tokyo Metropolitan Institute for Geriatrics and Gerontology (TMIG), Japan. Anne Murray, Berman Centre for Outcomes and Clinical Research, Hennepin Healthcare Research Institute, USA; University of Minnesota, USA. Anita Liberalesso Neri, Department of Educational Psychology, Faculty of Education, State University of Campinas, Brazil. Claudia Niessner, Institute of Sports and Sports Science, Karlsruhe Institute of Technology, Germany. Gabriel Núñez Othón, Division of Biological Sciences and Health, University of Sonora, Mexico. Gabriel Olveira, Servicio de Endocrinología y Nutrición, Hospital Regional Universitario de Málaga, Spain; IBIMA/plataforma Bionand, Spain; Departamento de Medicina y Dermatología, Universidad de Málaga, Spain; CIBER de Diabetes y Enfermedades Metabólicas Asociadas, Instituto de Salud Carlos III, Spain. Suzanne G. Orchard, School of Public Health and Preventive Medicine, Monash University, Australia. Andrezj Pajak, Department of Epidemiology and Population Studies, Jagellonian University Collegium Medicum, Poland. Chan Woong Park, Department of Kinesiology, College of Health & Human Services, Sacramento State University, USA. Julie A. Pasco, Deakin University, Institute for Mental and Physical Health and Clinical Translation (IMPACT), Australia; Department of Medicine—Western Health, The University of Melbourne, Australia. Maria E. Peña Reyes, Escuela Nacional de Antropologia e Historia, Instituto Nacional de Antropologia e Historia, Mexico. Leani Souza Máximo Pereira, Postgraduate program in Health Sciences at the Faculty of Medical Sciences of Minas Gerais, Brazil. Annette Peters, Institute of Epidemiology, Helmholtz Zentrum München, German Research Center for Environmental Health (GmbH), Germany; Institute for Medical Information Processing, Biometry and Epidemiology (IBE), Faculty of Medicine, LMU Munich, Pettenkofer School of Public Health, Germany. Eric Tsz-Chun Poon, Department of Sports Science and Physical Education, The Chinese University of Hong Kong, Hong Kong, China. Margareth C. Portela, Sergio Arouca National School of Public Health, Oswaldo Cruz Foundation, Brazil. Jedd Pratt, Institute for Sport and Health, University College Dublin, Ireland; Department of Sport and Exercise Sciences, Manchester Metropolitan University Institute of Sport, UK. Robinson Ramírez-Vélez, Navarrabiomed, Hospital Universitario de Navarra (HUN)-Universidad Pública de Navarra (UPNA), IdiSNA, Spain; CIBER of Frailty and Healthy Aging (CIBERFES), Instituto de Salud Carlos III, Spain. Wendy Rodríguez-García, Licenciatura en Nutriología, Facultad de Estudios Superiores Zaragoza, Universidad Nacional Autónoma de México, Mexico. Joanne Ryan, School of Public Health and Preventive Medicine, Monash University, Australia. Mauricio A. San-Martín, Instituto de Ciencias del Movimiento y la Ocupación Humana, Facultad de Medicina, Universidad Austral de Chile, Chile. Francisco José Sánchez-Torralvo, Servicio de Endocrinología y Nutrición, Hospital Regional Universitario de Málaga, Spain; IBIMA/plataforma Bionand, Spain. Mahnaz Saremi, Workplace Health Promotion Research Center, School of Public Health and Safety, Shahid Beheshti University of Medical Sciences, Iran. Arno Schmidt-Trucksäss, Department of Sport, Exercise and Health, University of Basel, Switzerland. Satoshi Seino, Tokyo Metropolitan Institute for Geriatrics and Gerontology, Japan. Shamsul Azhar Shah, Department of Public Health Medicine, Faculty of Medicine, Universiti Kebangsaan Malaysia, Malaysia. Marc Sim, Nutrition & Health Innovation Research Institute, School of Medical and Health Sciences, Edith Cowan University, Australia; Medical School, The University Western Australia, Australia. Bjørn Heine Strand, Department of Physical Health and Ageing, Norwegian Institute of Public Health, Norway. Mythily Subramaniam, Research Division, Institute of Mental Health, Singapore; Saw Swee Hock School of Public Health, National University of Singapore, Singapore. Charlotte Suetta, Department of Geriatric and Palliative Medicine, Copenhagen University Hospital, Bispebjerg and Frederiksberg, Denmark; Department of Clinical Medicine, Faculty of Health, University of Copenhagen, Denmark. Sophia X. Sui, Deakin University, Institute for Mental and Physical Health and Clinical Translation (IMPACT), Australia. Jonas S. Sundarakumar, Centre for Brain Research, Indian Institute of Science, India. Koya Suzuki, Graduate School of Health and Sports Science, Juntendo University, Japan. Abdonas Tamosiunas, Institute of Cardiology, Medical Academy, Lithuanian University of Health Sciences, Lithuania. Maw Pin Tan, Division of Geriatric Medicine, Department of Medicine, Universiti Malaya, Malaysia. Yu Taniguchi, Tokyo Metropolitan Institute of Gerontology, Japan. Barbara Thorand, Institute of Epidemiology, Helmholtz Zentrum München, German Research Center for Environmental Health (GmbH), Germany; Institute for Medical Information Processing, Biometry and Epidemiology (IBE), Faculty of Medicine, LMU Munich, Pettenkofer School of Public Health, Germany. Anna Turusheva, The North-Western State Medical University named after I.I. Mechnikov, Russia. Anne Therese Tveter, Center for treatment of Rheumatic and Musculoskeletal Diseases (REMEDY), Health Service Research and Innovation Unit, Diakonhjemmet Hospital, Norway; Department of Rehabilitation Science and Health Technology, Institute of Health Sciences, Oslo Metropolitan University, Norway. Jonathan Wagner, Department of Sport, Exercise and Health, University of Basel, Switzerland. Dao Wang, Physical Fitness Research and Health Guidance Center, Shanghai Research Institute of Sports Science (Shanghai Anti-Doping Agency), China. Stuart J. Warden, Department of Physical Therapy, School of Health and Human Sciences, Indiana University Indianapolis, USA. Julia Wearing, School for Interprofessional Health Care, Cooperative State University Baden-Wuerttemberg, Germany. Shiou Liang Wee, Health and Social Sciences Cluster, Singapore Institute of Technology, Singapore; Geriatric Education and Research Institute, Singapore. Leo D. Westbury, MRC Lifecourse Epidemiology Centre, University of Southampton, UK. Agnieszka Wiśniowska-Szurlej, Institute of Health Sciences, Medical College of Rzeszow University, Poland. Alexander Woll, Institute of Sports and Sports Science, Karlsruhe Institute of Technology, Germany. Noriko Yoshimura, Department of Preventive Medicine for Locomotive Organ Disorders, 22^nd^ Century Medical and Research Center, The University of Tokyo, Japan. Ruby Yu, Department of Medicine and Therapeutics, Faculty of Medicine, The Chinese University of Hong Kong, Hong Kong, China; CUHK Jockey Club Institute of Ageing, The Chinese University of Hong Kong, Hong Kong, China.

## Supplementary materials

Image, application 1

Image, video 2

## Footnotes

Supplementary materials associated with this article can be found in the online version at doi:10.1016/j.jshs.2024.101014.
