# Supplementary material for: International norms for adult handgrip strength: A systematic review of data on 2.4 million adults aged 20 to 100+ years from 69 countries and regions
Source: J Sport Health Sci. 2024 Dec 6;14:101014. doi: 10.1016/j.jshs.2024.101014 (PMC11863340; doi:10.1016/j.jshs.2024.101014)
Supplement: Supplementary file 1 [file mmc1.pdf]

Electronic Supplementary Material

Article title: International norms for adult handgrip strength: A systematic review of data on 2.4 million adults aged 20 to 100+ years from 69 countries and regions

Journal name: Journal of Sport and Health Science.

Authors' names

Grant R. Tomkinson[a,*], Justin J. Lang[a, b, c], Lukáš Rubín[d, e], Ryan McGrath[a, f, g, h, i], Bethany Gower[a], Terry Boyle[j], Marilyn G. Klug[k], Alexandra J. Mayhew[l, m, n], Henry T. Blake[a], Francisco B. Ortega[o, p, q], Cristina Cadenas-Sanchez[o, p, r, s], Costan G. Magnussen[a, t, u, v], Brooklyn J. Fraser[a, w], Tetsuhiro Kidokoro[a, x], Yang Liu[y,z], Kaare Christensen[aa], Darryl P. Leong[bb] & the iGRIPS (international handGRIP Strength) group[†]

Institutional affiliations

[a] Alliance for Research in Exercise, Nutrition and Activity (ARENA), Allied Health and Human Performance, University of South Australia, Adelaide, SA 5000, Australia

[b] Centre for Surveillance and Applied Research, Public Health Agency of Canada, Ottawa, ON K1A 0K9, Canada

[c] School of Epidemiology and Public Health, Faculty of Medicine, University of Ottawa, Ottawa, ON K1H 8M5, Canada

[d] Department of Physical Education and Sport, Faculty of Science, Humanities and Education, Technical University of Liberec, Liberec 461 17, Czech Republic

[e] Institute of Active Lifestyle, Faculty of Physical Culture, Palacký University Olomouc, Olomouc, 779 00, Czech Republic

[f] Healthy Aging North Dakota (HAND), North Dakota State University, Fargo, ND 58102, USA

[g] Department of Health, Nutrition, and Exercise Sciences, North Dakota State University, Fargo, ND 58108, USA

[h] Fargo VA Healthcare System, Fargo, ND 58102, USA

[i] Department of Geriatrics, University of North Dakota, Grand Forks, ND 58202, USA

[j] Australian Centre for Precision Health, Allied Health and Human Performance, University of South Australia, Adelaide, SA 5000, Australia

[k] Department of Population Health, University of North Dakota, Grand Forks, ND 58202, USA

[l] Department of Health Research Methods, Evidence, and Impact, Faculty of Health Sciences, McMaster University, Hamilton, ON L8S 4L8, Canada

[m] Labarge Centre for Mobility in Aging, McMaster University, Hamilton, ON L8P 0A1, Canada

[n] McMaster Institute for Research on Aging, McMaster University, Hamilton, ON L8P 0A1, Canada

[o] Department of Physical Education and Sports, Faculty of Sport Sciences, Sport and Health University Research Institute (iMUDS), University of Granada, Granada, ES 18071, Spain

[p] Centro de Investigación Biomédica en Red Fisiopatología de la Obesidad y Nutrición (CIBERobn), Instituto de Salud Carlos III, Granada, ES 18071, Spain

[q] Faculty of Sport and Health Sciences, University of Jyväskylä, Jyväskylä 40014, Finland
[r] Department of Cardiology, Stanford University, Stanford, CA 94305, USA
[s] Veterans Affairs Palo Alto Health Care System, Palo Alto, CA 94304, USA
[t] Baker Heart and Diabetes Institute, Melbourne, VIC 3004, Australia
[u] Research Centre of Applied and Preventive Cardiovascular Medicine, University of Turku, Turku 20520, Finland
[v] Centre for Population Health Research, University of Turku and Turku University Hospital, Turku 20520, Finland
[w] Menzies Institute for Medical Research, University of Tasmania, Hobart, TAS 7000, Australia
[x] Faculty of Sport Science, Nippon Sport Science University, Tokyo 158-8508, Japan
[y] School of Physical Education, Shanghai University of Sport, Shanghai 200438, China
[z] Shanghai Research Center for Physical Fitness and Health of Children and Adolescents, Shanghai 200438, China
[aa] Department of Public Health, Epidemiology, Biostatistics and Biodemography, University of Southern Denmark, Odense 5230, Denmark
[bb] The Population Health Research Institute, McMaster University and Hamilton Health Sciences, Hamilton, ON L8L 2X2, Canada

* **Corresponding author**
Prof. Grant R. TOMKINSON
🖂 Alliance for Research in Exercise Nutrition and Activity (ARENA)
Allied Health & Human Performance
University of South Australia
GPO Box 2471
Adelaide SA 5001
Australia
🖰 grant.tomkinson@unisa.edu.au

[†] The names and affiliations of the iGRIPS (international handGRIP Strength) group.

Mette Aadahl, Centre for Clinical Research and Prevention, Bispebjerg and Frederiksberg Hospital, Denmark; Department of Clinical Medicine, Faculty of Health and Medical Sciences, University of Copenhagen, Denmark. Edimansyah Abdin, Research Division, Institute of Mental Health, Singapore. Julian Alcazar, GENUD Toledo Research Group, Faculty of Sports Sciences, University of Castilla-La Mancha, Spain; Department of Geriatric and Palliative Medicine, Copenhagen University Hospital, Bispebjerg and Frederiksberg, Denmark; Centro de Investigación Biomédica en Red Fragilidad y Envejecimiento Saludable (CIBERFES), Instituto de Salud Carlos III, Spain; Grupo Mixto de Fragilidad y Envejecimiento Exitoso UCLM-SESCAM, Universidad de Castilla-La Mancha-Servicio de Salud de Castilla-La Mancha, IDISCAM, Spain. Aqeel Alenazi, Department of Health and Rehabilitation Sciences, College of Applied Medical Sciences, Prince Sattam Bin Abdulaziz University, Saudi Arabia. Bader Alqahtani, Department of Health and Rehabilitation Sciences, College of Applied Medical Sciences, Prince Sattam Bin Abdulaziz University, Saudi Arabia. Cledir De A. Amaral, Federal Institute of Acre, Brazil. Thatiana L. M. Amaral, Federal University of Acre, Brazil. Alex Andrade Fernandes, Instituto Federal de Educação, Ciência e Tecnologia de Minas Gerais—Campus Ipatinga, Brazil. Peter Axelsson, Department of Hand Surgery, Sahlgrenska University Hospital, Sweden; Department of Clinical Sciences, Sahlgrenska Academy, University of Gothenburg, Sweden. Jennifer N. Baldwin, Sydney School of Health Sciences, Faculty of Medicine and Health, The University of Sydney, Australia. Karin Bammann, Institute for Public Health and Nursing Sciences, University of Bremen, Germany. Aline R. Barbosa, Centro de Desportos, Universidade Federal de Santa Catarina, Brazil. Ameline Bardo, UMR 7194-HNHP, CNRS-MNHN, Département Homme et Environnement, Musée de l'Homme, Paris, France; Department of Human Origins, Max Planck Institute for Evolutionary Anthropology, Germany. Inosha Bimali, Department of Physiotherapy, Kathmandu University School of Medical Sciences, Nepal. Peter Bjerregaard, National Institute of Public Health, University of Southern Denmark, Denmark. Martin Bobak, International Institute for Health and Society, Department of Epidemiology and Public Health, University College London, UK. Colin A. Boreham, Institute for Sport and Health, University College Dublin, Ireland. Klaus Bös, Institute of Sports and Sports Science, Karlsruhe Institute of Technology, Germany. João Carlos Bouzas Marins, Physical Education Department, Federal University of Viçosa, Brazil. Joshua Burns, Disability Prevention Program, St. Jude Children's Research Hospital, USA. Nadezda Capkova, Environmental and Population Health Monitoring Centre, National Institute of Public Health, Czech Republic. Lilia Castillo-Martínez, Clinical Nutrition Department, Instituto Nacional de Ciencias Médicas y Nutrición Salvador Zubirán, Mexico. Liang-Kung Chen, Center for Geriatrics and Gerontology, Taipei Veterans General Hospital; Center for Healthy Longevity and Aging Sciences, National Yang Ming Chiao Tung University; Taipei Municipal Gan-Dau Hospital. Siu Ming Choi, Faculty of Education, University of Macau, Macao, China. Rebecca K. J. Choong, Department of Medicine, Universiti Malaya Medical Centre, Malaysia. Susana C. Confortin, Postgraduate Program in Public Health (PPGSCol), University of the Extreme South of Santa Catarina (UNESC), Brazil. Cyrus Cooper, MRC Lifecourse Epidemiology Centre, University of Southampton,

UK; NIHR Southampton Biomedical Research Centre, University of Southampton and University Hospital Southampton NHS Foundation Trust, UK; NIHR Oxford Biomedical Research Centre, University of Oxford, UK. Jorge E. Correa-Bautista, Facultad de Ciencias del Deporte y la Educación Física, Universidad de Cundinamarca, Colombia. Amandine Cournil, Mission pour la Science Ouverte, Institut de Recherche pour le Développement, France. Grace Cruz, Population Institute, University of the Philippines, Philippines. Eling D. de Bruin, Institute of Human Movement Sciences and Sport (IBWS), Department of Health Sciences and Technology, ETH Zurich, Switzerland; OST—Eastern Swiss University of Applied Sciences, Department of Health, Switzerland; Division of Physiotherapy, Department of Neurobiology, Care Sciences and Society, Karolinska Institute, Sweden. José Antonio De Paz, Institute of Biomedicine, University of León, Spain; Division of Biological Sciences and Health, University of Sonora, Mexico. Bruno De Souza Moreira, Center for Studies in Public Health and Aging—Federal University of Minas Gerais and Oswaldo Cruz Foundation-Minas Gerais, Brazil. Luiz Antonio Dos Anjos, Departamento de Nutrição Social, Faculdade de Nutrição Emilia de Jesus Ferreiro, Universidade Federal Fluminense (UFF), Brazil. María Cristina Enríquez Reyna, Universidad Autónoma de Nuevo León, Facultad de Organización Deportiva Monterrey, Mexico. Eduardo Ferriolli, Department of Internal Medicine, Ribeirao Preto Medical School, University of São Paulo, Brazil. Gillian Forrester, School of Psychology, University of Sussex, UK. Elena Frolova, The North-Western State Medical University named after I.I. Mechnikov, Russia. Abadi K. Gebre, Nutrition & Health Innovation Research Institute, School of Medical and Health Sciences, Edith Cowan University, Australia; School of Pharmacy, College of Health Sciences, Mekelle University, Mekelle, Ethiopia. Atef M. Ghaleb, Department of Industrial Engineering, College of Engineering, Alfaisal University, Saudi Arabia. Tiffany K. Gill, Adelaide Medical School, The University of Adelaide, Australia; Alliance for Research in Exercise, Nutrition and Activity (ARENA), Allied Health and Human Performance, University of South Australia, Australia. Yasuyuki Gondo, Graduate School of Human Sciences, Osaka University, Japan. M. Cristina Gonzalez, Postgraduate Program in Nutrition and Food, Federal University of Pelotas, Brazil; Pennington Biomedical Research Center, USA. Citlali Gonzalez Alvarez, Escuela Nacional de Antropologia e Historia, Instituto Nacional de Antropologia e Historia, Mexico. Mary K. Hannah, MRC/CSO Social and Public Health Sciences Unit, School of Health and Wellbeing, University of Glasgow, UK. Nicholas C. Harvey, MRC Lifecourse Epidemiology Centre, University of Southampton, UK; NIHR Southampton Biomedical Research Centre, University of Southampton and University Hospital Southampton NHS Foundation Trust, UK. Jean-Yves Hogrel, Neuromuscular Investigation Center, Institute of Myology, France. Marie-Theres Huemer, Institute of Epidemiology, Helmholtz Zentrum München, German Research Center for Environmental Health (GmbH), Germany. Toshiko Iidaka, Department of Preventive Medicine for Locomotive Organ Disorders, 22nd Century Medical and Research Center, The University of Tokyo, Japan. Lewis A. Ingram, Alliance for Research in Exercise, Nutrition and Activity (ARENA), Allied Health and Human Performance, University of South Australia, Australia. Dmitri A. Jdanov, Max Planck Institute for Demographic Research, Germany; National Research University Higher School of Economics, Russia. Victoria L. Keevil, Department of Medicine, University of Cambridge, UK. Wolfgang Kemmler, Institute of Radiology,

University-Hospital Erlangen, Germany; Institute of Medical Physics, University of Erlangen-Nürnberg, Germany. Rose Anne Kenny, The Irish Longitudinal Study on Ageing (TILDA), School of Medicine, Trinity College Dublin, Ireland. Dae-Yeon Kim, Measurement and Evaluation in Physical Education and Sport Science, Korea National Sport University, Republic of Korea. Tracy L. Kivell, Department of Human Origins, Max Planck Institute for Evolutionary Anthropology, Germany. Ingirid G. H. Kjær, Department of Sport Science and Physical Education, The University of Agder, Norway. Alexander Kluttig, Institute of Medical Epidemiology, Biometrics and Informatics, Interdisciplinary Center for Health Sciences, Medical Faculty of the Martin-Luther-University Halle-Wittenberg, Germany. Rumi Kozakai, Department of Health and Welfare Science, School of Lifelong Sport, Hokusho University, Japan; Department of Epidemiology of Aging, Research Institute, National Center for Geriatrics and Gerontology, Japan. Danit Langer, School of Occupational Therapy, Faculty of Medicine, Hebrew University, Israel. Lisbeth A. Larsen, Department of Public Health, Epidemiology, Biostatistics and Biodemography, University of Southern Denmark, Denmark. Wei-Ju Lee, Center for Healthy Longevity and Aging Sciences, National Yang Ming Chiao Tung University; Department of Family Medicine, Taipei Veterans General Hospital Yuanshan Branch. David A. Leon, Faculty of Epidemiology and Population Health, London School of Hygiene & Tropical Medicine, UK. Eric Lichtenstein, Department of Sport, Exercise and Health, University of Basel, Switzerland. Bertis B. Little, School of Public Health and Information Sciences, University of Louisville, USA. Roberto Alves Lourenço, Research Laboratory on Human Aging—GeronLab, Internal Medicine Department, Faculty of Medical Sciences, State University of Rio de Janeiro, Brazil; Department of Medicine, Pontifical Catholic University, Brazil. Rahul Malhotra, Centre for Ageing Research and Education, Duke-National University of Singapore Medical School, Singapore; Health Services and Systems Research, Duke-National University of Singapore Medical School, Singapore. Robert M. Malina, Department of Kinesiology and Health Education, University of Texas, USA; School of Public Health and Information Sciences, University of Louisville, USA. Kiyoaki Matsumoto, Graduate School of Human Sciences, Osaka University, Japan. Tal Mazor-Karsenty, School of Occupational Therapy, Faculty of Medicine, Hebrew University, Israel. Marnee J. McKay, Sydney School of Health Sciences, Faculty of Medicine and Health, The University of Sydney, Australia. Sinéad McLoughlin, The Irish Longitudinal Study on Ageing (TILDA), Trinity Central, Trinity College Dublin, Ireland. Abhishek L. Mensegere, Centre for Brain Research, Indian Institute of Science, India. Mostafa Mohammadian, Health Foresight and Innovation Research Center, Institute for Futures Studies in Health, Kerman University of Medical Sciences, Iran. Virgilio Garcia Moreira, Research Laboratory on Human Aging—GeronLab, Internal Medicine Department, Faculty of Medical Sciences, State University of Rio de Janeiro, Brazil. Hiroshi Murayama, Tokyo Metropolitan Institute for Geriatrics and Gerontology (TMIG), Japan. Anne Murray, Berman Centre for Outcomes and Clinical Research, Hennepin Healthcare Research Institute, USA; University of Minnesota, USA. Anita Liberalesso Neri, Department of Educational Psychology, Faculty of Education, State University of Campinas, Brazil. Claudia Niessner, Institute of Sports and Sports Science, Karlsruhe Institute of Technology, Germany. Gabriel Núñez Othón, Division of Biological Sciences and Health, University of Sonora, Mexico. Gabriel Olveira, Servicio de Endocrinología y Nutrición, Hospital Regional Universitario de

Málaga, Spain; IBIMA/plataforma Bionand, Spain; Departamento de Medicina y Dermatología, Universidad de Málaga, Spain; CIBER de Diabetes y Enfermedades Metabólicas Asociadas, Instituto de Salud Carlos III, Spain. Suzanne G. Orchard, School of Public Health and Preventive Medicine, Monash University, Australia. Andrezj Pajak, Department of Epidemiology and Population Studies, Jagellonian University Collegium Medicum, Poland. Chan Woong Park, Department of Kinesiology, College of Health & Human Services, Sacramento State University, USA. Julie A. Pasco, Deakin University, Institute for Mental and Physical Health and Clinical Translation (IMPACT), Australia; Department of Medicine—Western Health, The University of Melbourne, Australia. Maria E. Peña Reyes, Escuela Nacional de Antropologia e Historia, Instituto Nacional de Antropologia e Historia, Mexico. Leani Souza Máximo Pereira, Postgraduate program in Health Sciences at the Faculty of Medical Sciences of Minas Gerais, Brazil. Annette Peters, Institute of Epidemiology, Helmholtz Zentrum München, German Research Center for Environmental Health (GmbH), Germany; Institute for Medical Information Processing, Biometry and Epidemiology (IBE), Faculty of Medicine, LMU Munich, Pettenkofer School of Public Health, Germany. Eric Tsz-Chun Poon, Department of Sports Science and Physical Education, The Chinese University of Hong Kong, Hong Kong, China. Margareth C. Portela, Sergio Arouca National School of Public Health, Oswaldo Cruz Foundation, Brazil. Jedd Pratt, Institute for Sport and Health, University College Dublin, Ireland; Department of Sport and Exercise Sciences, Manchester Metropolitan University Institute of Sport, UK. Robinson Ramírez-Vélez, Navarrabiomed, Hospital Universitario de Navarra (HUN)-Universidad Pública de Navarra (UPNA), IdiSNA, Spain; CIBER of Frailty and Healthy Aging (CIBERFES), Instituto de Salud Carlos III, Spain. Wendy Rodríguez-García, Licenciatura en Nutriología, Facultad de Estudios Superiores Zaragoza, Universidad Nacional Autónoma de México, Mexico. Joanne Ryan, School of Public Health and Preventive Medicine, Monash University, Australia. Mauricio A. San-Martín, Locomotor Apparatus and Rehabilitation Institute, Faculty of Medicine, Universidad Austral de Chile, Chile. Francisco José Sánchez-Torralvo, Servicio de Endocrinología y Nutrición, Hospital Regional Universitario de Málaga, Spain; IBIMA/plataforma Bionand, Spain. Mahnaz Saremi, Workplace Health Promotion Research Center, School of Public Health and Safety, Shahid Beheshti University of Medical Sciences, Iran. Arno Schmidt-Trucksäss, Department of Sport, Exercise and Health, University of Basel, Switzerland. Satoshi Seino, Tokyo Metropolitan Institute for Geriatrics and Gerontology, Japan. Shamsul Azhar Shah, Department of Public Health Medicine, Faculty of Medicine, Universiti Kebangsaan Malaysia, Malaysia. Marc Sim, Nutrition & Health Innovation Research Institute, School of Medical and Health Sciences, Edith Cowan University, Australia; Medical School, The University Western Australia, Australia. Bjørn Heine Strand, Department of Physical Health and Ageing, Norwegian Institute of Public Health, Norway. Mythily Subramaniam, Research Division, Institute of Mental Health, Singapore; Saw Swee Hock School of Public Health, National University of Singapore, Singapore. Charlotte Suetta, Department of Geriatric and Palliative Medicine, Copenhagen University Hospital, Bispebjerg and Frederiksberg, Denmark; Department of Clinical Medicine, Faculty of Health, University of Copenhagen, Denmark. Sophia X. Sui, Deakin University, Institute for Mental and Physical Health and Clinical Translation (IMPACT), Australia. Jonas S. Sundarakumar, Centre for Brain Research, Indian Institute of

Science, India. Koya Suzuki, Graduate School of Health and Sports Science, Juntendo University, Japan. Abdonas Tamosiunas, Institute of Cardiology, Medical Academy, Lithuanian University of Health Sciences, Lithuania. Maw Pin Tan, Division of Geriatric Medicine, Department of Medicine, Universiti Malaya, Malaysia. Yu Taniguchi, Tokyo Metropolitan Institute of Gerontology, Japan. Barbara Thorand, Institute of Epidemiology, Helmholtz Zentrum München, German Research Center for Environmental Health (GmbH), Germany; Institute for Medical Information Processing, Biometry and Epidemiology (IBE), Faculty of Medicine, LMU Munich, Pettenkofer School of Public Health, Germany. Anna Turusheva, The North-Western State Medical University named after I.I. Mechnikov, Russia. Anne Therese Tveter, Center for treatment of Rheumatic and Musculoskeletal Diseases (REMEDY), Health Service Research and Innovation Unit, Diakonhjemmet Hospital, Norway; Department of Rehabilitation Science and Health Technology, Institute of Health Sciences, Oslo Metropolitan University, Norway. Jonathan Wagner, Department of Sport, Exercise and Health, University of Basel, Switzerland. Dao Wang, Physical Fitness Research and Health Guidance Center, Shanghai Research Institute of Sports Science (Shanghai Anti-Doping Agency), China. Stuart J. Warden, Department of Physical Therapy, School of Health and Human Sciences, Indiana University Indianapolis, USA. Julia Wearing, School for Interprofessional Health Care, Cooperative State University Baden-Wuerttemberg, Germany. Shiou Liang Wee, Health and Social Sciences Cluster, Singapore Institute of Technology, Singapore; Geriatric Education and Research Institute, Singapore. Leo D. Westbury, MRC Lifecourse Epidemiology Centre, University of Southampton, UK. Agnieszka Wiśniowska-Szurlej, Institute of Health Sciences, Medical College of Rzeszow University, Poland. Alexander Woll, Institute of Sports and Sports Science, Karlsruhe Institute of Technology, Germany. Noriko Yoshimura, Department of Preventive Medicine for Locomotive Organ Disorders, 22nd Century Medical and Research Center, The University of Tokyo, Japan. Ruby Yu, Department of Medicine and Therapeutics, Faculty of Medicine, The Chinese University of Hong Kong, Hong Kong, China; CUHK Jockey Club Institute of Ageing, The Chinese University of Hong Kong, Hong Kong, China.

Supplementary Table 1. Preferred Reporting Items for Systematic reviews and Meta-Analyses (PRISMA) checklist.

Supplementary Table 2A. Search strategy terms applied in MEDLINE (via OVID, 1946 to 1 December 2023).

Supplementary Table 2B. Search strategy terms applied in SPORTDiscus (via EBSCOhost, 1930 to 1 December 2023).

Supplementary Table 2C. Search strategy terms applied in Embase (via OVID, 1947 to 1 December 2023).

Supplementary Table 2D. Search strategy terms applied in Web of Science (Core Collection, 1975 to 1 December 2023).

Supplementary Table 2E. Search strategy terms applied in CINAHL (via EBSCOhost, 1937 to 1 December 2023).

Supplementary Table 2F. Search strategy terms applied in Google Scholar (first 200 results sorted by relevance on 1 December 2023).

Supplementary Harmonization Methods. Methods for adjusting handgrip strength values to a common metric, test, and reporting protocol.

Supplementary Table 3A. Adjustment of absolute handgrip strength (in kilograms (kg)) for test protocol.

Supplementary Table 3B. Adjustment of normalized handgrip strength (handgrip strength in kilograms divided by height in meters squared (kg/m^2)) for test protocol.

Supplementary Table 3C. Adjustment of absolute handgrip strength (in kilograms (kg)) for reporting protocol.

Supplementary Table 3D. Adjustment of normalized handgrip strength (handgrip strength in kilograms divided by height in meters squared (kg/m^2)) for reporting protocol.

Supplementary Table 3E. Summary of absolute (in kilograms) and normalized handgrip strength (handgrip strength in kilograms divided by height in meters squared) adjustments for test and reporting protocol for the included studies by country or region.

Supplementary Fig. 1A. Sex- and age-specific box plots for absolute handgrip strength (in kilograms (kg)) generated from raw data (n = 366,367).

Supplementary Fig. 1B. Sex- and age-specific box plots for normalized handgrip strength (handgrip strength in kilograms divided by height in meters squared (kg/m^2)) generated from raw data (n = 357,063).

Supplementary Fig. 1C. Sex- and age-specific probability plots for absolute handgrip strength (in kilograms (kg)) generated from raw data (n = 366,367).

Supplementary Fig. 1D. Sex- and age-specific probability plots for normalized handgrip strength (handgrip strength in kilograms divided by height in meters squared (kg/m^2)) generated from raw data (*n* = 357,063).

Supplementary Notes and Abbreviations. Table 1 notes and abbreviations.

Supplementary Table 4A. Descriptive characteristics of the included studies by country or region.

Supplementary Table 4B. Handgrip strength test protocols for the included studies by country or region.

Supplementary Table 5. Study quality scores based on the *standard quality assessment criteria for evaluating primary research papers from a variety of fields* tool.

Supplementary Fig. 2A. Smoothed percentile curves (P_5 to P_{95}) for absolute handgrip strength in kilograms (kg) for males aged 20 to 100+ years.

Supplementary Fig. 2B. Smoothed percentile curves (P_5 to P_{95}) for absolute handgrip strength in kilograms (kg) for females aged 20 to 100+ years.

Supplementary Fig. 2C. Smoothed percentile curves (P_5 to P_{95}) for normalized handgrip strength (handgrip strength in kilograms divided by height in meters squared (kg/m^2)) for males aged 20 to 100+ years.

Supplementary Fig. 2D. Smoothed percentile curves (P_5 to P_{95}) for normalized handgrip strength (handgrip strength in kilograms divided by height in meters squared (kg/m^2)) for females aged 20 to 100+ years.

Supplementary Funding.

Supplementary References.

1 **Supplementary Table 1.** Preferred Reporting Items for Systematic reviews and Meta-Analyses (PRISMA) checklist.

Section and Topic	Item #	Checklist item	Location where item is reported
**TITLE**			
Title	1	Identify the report as a systematic review.	Title page
**ABSTRACT**			
Abstract	2	See the PRISMA 2020 for Abstracts checklist.	Abstract
**INTRODUCTION**			
Rationale	3	Describe the rationale for the review in the context of existing knowledge.	Section 1
Objectives	4	Provide an explicit statement of the objective(s) or question(s) the review addresses.	Section 1
**METHODS**			
Eligibility criteria	5	Specify the inclusion and exclusion criteria for the review and how studies were grouped for the syntheses.	Section 2.2
Information sources	6	Specify all databases, registers, websites, organizations, reference lists and other sources searched or consulted to identify studies. Specify the date when each source was last searched or consulted.	Section 2.3
Search strategy	7	Present the full search strategies for all databases, registers and websites, including any filters and limits used.	Section 2.4 + Supplementary Table 2A–2F
Selection process	8	Specify the methods used to decide whether a study met the inclusion criteria of the review, including how many reviewers screened each record and each report retrieved, whether they worked independently, and if applicable, details of automation tools used in the process.	Section 2.5
Data collection process	9	Specify the methods used to collect data from reports, including how many reviewers collected data from each report, whether they worked independently, any processes for obtaining or confirming data from study investigators, and if applicable, details of automation tools used in the process.	Section 2.6
Data items	10a	List and define all outcomes for which data were sought. Specify whether all results that were compatible with each outcome domain in each study were sought (e.g. for all measures, time points, analyses), and if not, the methods used to decide which results to collect.	Section 2.6
	10b	List and define all other variables for which data were sought (e.g. participant and intervention characteristics, funding sources). Describe any assumptions made about any missing or unclear information.	Section 2.6
Study risk of bias assessment	11	Specify the methods used to assess risk of bias in the included studies, including details of the tool(s) used, how many reviewers assessed each study and whether they worked independently, and if applicable, details of automation tools used in the process.	Section 2.7
Effect measures	12	Specify for each outcome the effect measure(s) (e.g. risk ratio, mean difference) used in the synthesis or presentation of results.	Section 2.8 + Supplementary Harmonization Methods
Synthesis methods	13a	Describe the processes used to decide which studies were eligible for each synthesis (e.g. tabulating the study intervention characteristics and comparing against the planned groups for each synthesis (Item #5)).	Section 2.8 + Supplementary Harmonization Methods
	13b	Describe any methods required to prepare the data for presentation or synthesis, such as handling of missing summary statistics, or data conversions.	Section 2.8 + Supplementary Harmonization

Section and Topic	Item #	Checklist item	Location where item is reported
			Methods + Supplementary Table 3A–3E
	13c	Describe any methods used to tabulate or visually display results of individual studies and syntheses.	Section 2.8 + Supplementary Harmonization Methods + Supplementary Table 3A–3E
	13d	Describe any methods used to synthesize results and provide a rationale for the choice(s). If meta-analysis was performed, describe the model(s), method(s) to identify the presence and extent of statistical heterogeneity, and software package(s) used.	Section 2.8 + Supplementary Harmonization Methods + Supplementary Table 3A–3E
	13e	Describe any methods used to explore possible causes of heterogeneity among study results (e.g. subgroup analysis, meta-regression).	Not applicable
	13f	Describe any sensitivity analyses conducted to assess robustness of the synthesized results.	Not applicable
Reporting bias assessment	14	Describe any methods used to assess risk of bias due to missing results in a synthesis (arising from reporting biases).	Not applicable
Certainty assessment	15	Describe any methods used to assess certainty (or confidence) in the body of evidence for an outcome.	Not applicable
**RESULTS**			
Study selection	16a	Describe the results of the search and selection process, from the number of records identified in the search to the number of studies included in the review, ideally using a flow diagram.	Section 3.1
	16b	Cite studies that might appear to meet the inclusion criteria, but which were excluded, and explain why they were excluded.	Fig. 1
Study characteristics	17	Cite each included study and present its characteristics.	Section 3.2 + Table 1+ Supplementary Table 4A and 4B
Risk of bias in studies	18	Present assessments of risk of bias for each included study.	Section 3.3 + Supplementary Table 5
Results of individual studies	19	For all outcomes, present, for each study: (a) summary statistics for each group (where appropriate) and (b) an effect estimate and its precision (e.g. confidence/credible interval), ideally using structured tables or plots.	Supplementary Table 4A and 4B
Results of syntheses	20a	For each synthesis, briefly summarize the characteristics and risk of bias among contributing studies.	Section 3.3
	20b	Present results of all statistical syntheses conducted. If meta-analysis was done, present for each the summary estimate and its precision (e.g.	Section 3.4 +

Section and Topic	Item #	Checklist item	Location where item is reported
		confidence/credible interval) and measures of statistical heterogeneity. If comparing groups, describe the direction of the effect.	Tables 2 and 3 + Figs 3 and 4 + Supplementary Table 6A and 6B + Supplementary Fig. 2
	20c	Present results of all investigations of possible causes of heterogeneity among study results.	Not applicable
	20d	Present results of all sensitivity analyses conducted to assess the robustness of the synthesized results.	Not applicable
Reporting biases	21	Present assessments of risk of bias due to missing results (arising from reporting biases) for each synthesis assessed.	Not applicable
Certainty of evidence	22	Present assessments of certainty (or confidence) in the body of evidence for each outcome assessed.	Not applicable
**DISCUSSION**			
Discussion	23a	Provide a general interpretation of the results in the context of other evidence.	Section 4
	23b	Discuss any limitations of the evidence included in the review.	Section 4.1
	23c	Discuss any limitations of the review processes used.	Section 4.1
	23d	Discuss implications of the results for practice, policy, and future research.	Section 4
**OTHER INFORMATION**			
Registration and protocol	24a	Provide registration information for the review, including register name and registration number, or state that the review was not registered.	Section 2.1
	24b	Indicate where the review protocol can be accessed, or state that a protocol was not prepared.	Section 2.1
	24c	Describe and explain any amendments to information provided at registration or in the protocol.	Section 2.9
Support	25	Describe sources of financial or non-financial support for the review, and the role of the funders or sponsors in the review.	Declarations
Competing interests	26	Declare any competing interests of review authors.	Declarations
Availability of data, code and other materials	27	Report which of the following are publicly available and where they can be found: template data collection forms; data extracted from included studies; data used for all analyses; analytic code; any other materials used in the review.	Declarations

Supplementary Table 2A. Search strategy terms applied in MEDLINE (via OVID, 1946 to 1 December 2023).

#	Query
1	(hand strength or handgrip strength or hand-grip strength or grip strength or grip-strength or muscle strength or strength dynamomet* or grip dynamomet*).mp. [mp = title, book title, abstract, original title, name of substance word, subject heading word, floating sub-heading word, keyword heading word, organism supplementary concept word, protocol supplementary concept word, rare disease supplementary concept word, unique identifier, synonyms, population supplementary concept word, anatomy supplementary concept word]
2	limit 1 to humans
3	(reference value* or normative or norms or age related or age-related).mp. [mp = title, book title, abstract, original title, name of substance word, subject heading word, floating sub-heading word, keyword heading word, organism supplementary concept word, protocol supplementary concept word, rare disease supplementary concept word, unique identifier, synonyms, population supplementary concept word, anatomy supplementary concept word]
4	limit 3 to humans
5	(adult* or men or women or male* or female*).mp. [mp = title, book title, abstract, original title, name of substance word, subject heading word, floating sub-heading word, keyword heading word, organism supplementary concept word, protocol supplementary concept word, rare disease supplementary concept word, unique identifier, synonyms, population supplementary concept word, anatomy supplementary concept word]
6	limit 5 to humans
7	2 and 4 and 6
8	limit 7 to (full text and humans and “all adult (19 plus years)” and journal article)

Supplementary Table 2B. Search strategy terms applied in SPORTDiscus (via EBSCOhost, 1930 to 1 December 2023).

#	Query
S1	hand strength OR handgrip strength OR hand-grip strength OR grip strength OR grip-strength OR muscle strength OR strength dynamomet* OR grip dynamomet*
S2	reference value* OR normative OR norms OR age related OR age-related
S3	adult* OR men OR women OR male* OR female*
S4	S1 AND S2 AND S3

Notes: Limiters: Full Text; Peer Reviewed; Publication Type: Academic Journal; Document Type: Article.

Expanders: Apply related words; Apply equivalent subjects.

Search modes: Boolean/Phrase.

Supplementary Table 2C. Search strategy terms applied in Embase (via OVID, 1947 to 1 December 2023).

#	**Query**
1	(hand strength or handgrip strength or hand-grip strength or grip strength or grip-strength or muscle strength or strength dynamomet* or grip dynamomet*).mp. [mp = title, abstract, heading word, drug trade name, original title, device manufacturer, drug manufacturer, device trade name, keyword heading word, floating subheading word, candidate term word]
2	limit 1 to human
3	(reference value* or normative or norms or age related or age-related).mp. [mp = title, abstract, heading word, drug trade name, original title, device manufacturer, drug manufacturer, device trade name, keyword heading word, floating subheading word, candidate term word]
4	limit 3 to human
5	(adult* or men or women or male* or female*).mp. [mp = title, abstract, heading word, drug trade name, original title, device manufacturer, drug manufacturer, device trade name, keyword heading word, floating subheading word, candidate term word]
6	limit 5 to human
7	2 and 4 and 6
8	limit 7 to (full text and article and (adult <18 to 64 years> or aged <65+ years>))

Supplementary Table 2D. Search strategy terms applied in Web of Science (Core Collection, 1975 to 1 December 2023).

#	Query
1	((TS = ((hand strength OR handgrip strength OR hand-grip strength OR grip strength OR grip-strength OR muscle strength OR strength dynamomet* OR grip dynamomet*))) AND TS = ((reference value* OR normative OR norms OR age related OR age-related))) AND TS = ((adult* OR men OR women OR male* OR female*))

Notes: NOT Document Types: Proceedings Papers or Editorial Materials or Data Papers or Book Chapters or Letters or Meeting Abstracts or Book Reviews or Corrections or Notes or Reprints.

Supplementary Table 2E. Search strategy terms applied in CINAHL (via EBSCOhost, 1937 to 1 December 2023).

#	Query
S1	hand strength OR handgrip strength OR hand-grip strength OR grip strength OR grip-strength OR muscle strength OR strength dynamomet* OR grip dynamomet*
S2	reference value* OR normative OR norms OR age related OR age-related
S3	adult* OR men OR women OR male* OR female*
S4	S1 AND S2 AND S3

Notes: Limiters: Full Text; Peer Reviewed; Research Article; Exclude MEDLINE records; Human; Age Groups: All Adult.

Expanders: Apply related words; Apply equivalent subjects.

Search modes: Boolean/Phrase.

Supplementary Table 2F. Search strategy terms applied in Google Scholar (first 200 results sorted by relevance on 1 December 2023).

#	**Query**
1	(hand strength OR handgrip strength OR hand-grip strength OR grip strength OR grip-strength OR muscle strength OR strength dynamomet* OR grip dynamomet*) AND (reference value* OR normative OR norms OR age related OR age-related) AND (adult* OR men OR women OR male* OR female*)

Supplementary Harmonization Methods. Methods for adjusting handgrip strength values to a common metric, test, and reporting protocol.

First, we expressed age (in years at baseline) in closed 5-year age groups (e.g., 20–24 years, 25–29 years). For each closed age group, we used published or calculated mean and SD age values as measures of centrality and variability, respectively. When only age ranges were available, missing means were estimated as the mean of the lower and upper age values, and missing SDs were estimated as the mean SD from all studies that reported SDs for closed 5-year age groups. For studies with open-ended age groups (e.g., 85+ years), we used the published or calculated mean and SD age values, or estimated the age range using the adjacent age group (e.g., 80–84 years) and then the means and SDs as above.

Second, we adjusted handgrip strength (HGS) values to a common test metric. We expressed absolute HGS values in kilograms (kg), which we occasionally converted from Newtons by dividing by 9.81 and from pounds by dividing by 2.21. While mean absolute HGS values were always available, and SDs were nearly always available, we estimated SDs when missing by multiplying the corresponding standard errors (SEs) by the square root of sample size. We normalized HGS by height (Ht) in meters (m) squared (i.e., HGS/Ht^2 in kg/m^2) because this is the most appropriate single body size dimension associated with HGS identified by allometry [1]. We calculated mean and SD normalized HGS values in most instances because such data were rarely reported. When missing, we estimated mean normalized HGS by dividing mean absolute HGS in kilograms by mean height in meters squared, the latter of which was occasionally converted from centimeters by dividing by 100 or from inches by dividing by 39.37. To verify the accuracy of this procedure, we calculated country-sex-age-specific mean normalized HGS values from available raw data (n = 366,367) and compared them to estimated mean normalized HGS values from mean absolute HGS divided by mean height-squared, and found a perfect correlation (ICC = 1.00) and no systematic difference (standardized (Cohen's) effect size (ES) = 0.00). Missing SDs for normalized HGS were estimated by: (a) calculating the sample-weighted mean coefficients of variation (CV) from all calculated mean and SD normalized HGS values for males (CV (95%CI) = 0.176 (0.170, 0.182)) and females (CV (95%CI) = 0.203 (0.196, 0.209)), and (b) multiplying the estimated mean normalized HGS values by the corresponding sex-specific estimated mean CV value.

Third, we adjusted HGS values to a reference test protocol. For both absolute and normalized HGS, we used Poisson regression models generated from available raw data (*n* = 366,367) to estimate the relative difference between the reference hand dynamometer and other dynamometer types, and between reference testing positions and other positions (see Supplementary Tables 3A, 3B, and 3E). The dependent variables in these models were either absolute or normalized HGS, and the independent variables were dynamometer type (hydraulic [reference], electronic, or mechanical), body position (seated [reference] or standing), elbow position (flexed [reference] or extended), radioulnar position (neutral [reference] or supinated), and handle position (adjusted to hand size [reference] or adjusted to standard position), as well as age, sex, country, testing year, sample base (national or non-national), sampling strategy (probability or non-probability), testing hand (both or single), number of reps per hand (1, 2, or 3), and the summary statistic for normative data reporting (average or maximum). We neither statistically adjusted for wrist position (because all studies reported using a neutral wrist position) nor shoulder position (because the few studies that used an abducted position reported it as ‘slight’ [i.e., <45°], which we considered to be practically adducted). We used the inverse of the relative risks from the Poisson regression models as our adjustment factors.

Lastly, we adjusted HGS values to a reference reporting protocol. For both absolute and normalized HGS, we used within-participant raw data (*n* = 69,528) to estimate the adjustment factors for testing hand, the number of reps per hand, and summary statistic (see Supplementary Tables 3C, 3D, and 3E). The reference values in these models were: (a) for absolute HGS, the maximum HGS value obtained from three trials on each hand, and (b) for normalized HGS, the maximum HGS value obtained from three trials on each hand divided by height-squared. We calculated the percentage of the HGS values from all other combinations of testing hand (left, right, non-dominant, dominant, or both [right and left]), number of reps per hand (1, 2, or 3), and summary statistic (maximum, average, or average of maxima) relative to the reference value (i.e., divided the value by the reference value) for each participant. We then calculated the mean of each calculated value (representing the relative risk), with the inverse of those means used as the adjustment factors.

Supplementary Table 3A. Adjustment of absolute handgrip strength (in kilograms (kg)) for test protocol.

Protocol variants	Mean absolute HGS (kg)	Percent mean difference from reference (RR)	Adjustment (multiplication) factor (1/RR)
**Dynamometer type**			
Hydraulic (*Reference*)	31.1	**Ref (1.000)**	**Ref (1.000)**
Electronic	31.7	0.976	1.024
Mechanical	32.8	0.952	1.050
**Body position**			
Seated (*Reference*)	28.5	**Ref (1.000)**	**Ref (1.000)**
Standing	33.7	1.107	0.903
**Elbow position**			
Flexed (*Reference*)	32.3	**Ref (1.000)**	**Ref (1.000)**
Extended	32.6	0.936	1.069
**Radio-ulnar position**			
Neutral (*Reference*)	32.4	**Ref (1.000)**	**Ref (1.000)**
Supinated	23.4	1.005	0.995
**Handle position**			
Adjusted to hand size (*Reference*)	32.4	**Ref (1.000)**	**Ref (1.000)**
Adjusted to standard position	34.8	0.977	1.023

Notes*:* Risk ratios were determined using Poisson regression models adjusted for age, sex, country, testing year, sample base (national or non-national), sampling strategy (probability or non-probability), testing hand (both or single), reps per hand (1, 2, or 3), summary statistic for normative data reporting (average or maximum), and all other tabulated variables.
Abbreviations: HGS = handgrip strength; Ref = reference; RR = risk ratio.

Supplementary Table 3B. Adjustment of normalized handgrip strength (handgrip strength in kilograms (kg) divided by height in meters squared (kg/m^2)) for test protocol.

Protocol variants	Mean normalized HGS (kg/m^2)	Percent mean difference from reference (RR)	Adjustment (multiplication) factor (1/RR)
**Dynamometer type**			
Hydraulic (*Reference*)	11.0	**Ref (1.000)**	**Ref (1.000)**
Electronic	11.6	0.977	1.024
Mechanical	11.8	0.956	1.046
**Body position**			
Seated (*Reference*)	10.8	**Ref (1.000)**	**Ref (1.000)**
Standing	11.9	1.107	0.903
**Elbow position**			
Flexed (*Reference*)	11.7	**Ref (1.000)**	**Ref (1.000)**
Extended	11.8	0.972	1.029
**Radio-ulnar position**			
Neutral (*Reference*)	11.7	**Ref (1.000)**	**Ref (1.000)**
Supinated	9.4	1.026	0.974
**Handle position**			
Adjusted to hand size (*Reference*)	11.7	**Ref (1.000)**	**Ref (1.000)**
Adjusted to standard position	12.3	0.984	1.016

Notes: Risk ratios were determined using Poisson regression models adjusted for age, sex, country, testing year, sample base (national or non-national), sampling strategy (probability or non-probability), testing hand (both or single), reps per hand (1, 2, or 3), summary statistic for normative data reporting (average or maximum), and all other tabulated variables.

Abbreviations: HGS = handgrip strength; m = meters; Ref = reference; RR = risk ratio.

Supplementary Table 3C. Adjustment of absolute handgrip strength (in kilograms (kg)) for reporting protocol.

	Number of reps per hand							
	1		2			3		
	Descriptive statistic							
Testing hand	Max	Ave	Max	Ave	Ave of maxima	Max	Ave	Ave of maxima
**Left**								
Mean absolute HGS (kg)	28.5		30.0	28.8		30.7	29.0	
Percent mean difference from reference (RR)	0.858		0.904	0.866		0.924	0.871	
Adjustment (multiplication) factor (1/RR)	1.166		1.106	1.155		1.082	1.148	
**Right**								
Mean absolute HGS (kg)	30.1		31.8	30.5		32.5	30.7	
Percent mean difference from reference (RR)	0.903		0.955	0.915		0.977	0.921	
Adjustment (multiplication) factor (1/RR)	1.107		1.047	1.093		1.024	1.086	
**Non-dominant**								
Mean absolute HGS (kg)	28.4		30.0	28.8		30.6	28.9	
Percent mean difference from reference (RR)	0.855		0.901	0.863		0.922	0.868	
Adjustment (multiplication) factor (1/RR)	1.170		1.110	1.159		1.085	1.152	
**Dominant**								
Mean absolute HGS (kg)	30.2		31.9	30.6		32.6	30.8	
Percent mean difference from reference (RR)	0.906		0.957	0.918		0.979	0.924	
Adjustment (multiplication) factor (1/RR)	1.104		1.045	1.089		1.021	1.082	
**Both**								
Mean absolute HGS (kg)	31.1	29.3	32.5	29.7	30.9	33.2	29.8	31.6
Percent mean difference from reference (RR)	0.937	0.880	0.980	0.891	0.929	**Ref (1.000)**	0.896	0.951
Adjustment (multiplication) factor (1/RR)	1.067	1.136	1.020	1.122	1.076	**Ref (1.000)**	1.116	1.052

Abbreviations: Ave = average value; Ave of maxima = average of the maximum values for each hand; HGS = handgrip strength; Max = maximum value; Ref = reference; RR = risk ratio.

Supplementary Table 3D. Adjustment of normalized handgrip strength (handgrip strength in kilograms (kg) divided by height in meters squared (kg/m^2)) for reporting protocol.

	Number of reps per hand							
	1		2			3		
	Descriptive statistic							
Testing hand	Max	Ave	Max	Ave	Ave of maxima	Max	Ave	Ave of maxima
**Left**								
Mean normalized HGS (kg/m^2)	10.3		10.8	10.4		11.1	10.5	
Percent mean difference from reference (RR)	0.858		0.904	0.867		0.925	0.871	
Adjustment (multiplication) factor (1/RR)	1.166		1.106	1.153		1.081	1.148	
**Right**								
Mean normalized HGS (kg/m^2)	10.9		11.5	11.0		11.7	11.1	
Percent mean difference from reference (RR)	0.903		0.955	0.915		0.977	0.921	
Adjustment (multiplication) factor (1/RR)	1.107		1.047	1.093		1.024	1.086	
**Non-dominant**								
Mean normalized HGS (kg/m^2)	10.3		10.8	10.4		11.1	10.4	
Percent mean difference from reference (RR)	0.855		0.901	0.864		0.922	0.868	
Adjustment (multiplication) factor (1/RR)	1.170		1.110	1.157		1.085	1.152	
**Dominant**								
Mean normalized HGS (kg/m^2)	10.9		11.5	11.0		11.7	11.1	
Percent mean difference from reference (RR)	0.906		0.958	0.918		0.980	0.924	
Adjustment (multiplication) factor (1/RR)	1.104		1.044	1.089		1.020	1.082	
**Both**								
Mean normalized HGS (kg/m^2)	11.2	10.6	11.7	10.7	11.1	12.0	10.8	11.4
Percent mean difference from reference (RR)	0.937	0.881	0.980	0.891	0.930	**Ref (1.000)**	0.896	0.951

Adjustment (multiplication) factor (1/RR)	1.067	1.135	1.020	1.122	1.075	**Ref (1.000)**	1.116	1.052

Abbreviations*:* Ave = average value; Ave of maxima = average of the maximum values for each hand; HGS = handgrip strength; m = meters; Max = maximum value; Ref = reference; RR = risk ratio.

Supplementary Table 3E. Summary of absolute (in kilograms (kg)) and normalized handgrip strength (handgrip strength in kilograms divided by height in meters squared (kg/m^2)) adjustments for test and reporting protocol for the included studies by country or region.

		Absolute handgrip strength							Normalized handgrip strength						
Country or region	Study	Dyna-mometer	Body position	Elbow position	RU position	Handle position	Hand + Reps/hand + Summary statistic	Overall	Dyna-mometer	Body position	Elbow position	RU position	Handle position	Hand + Reps/hand + Summary statistic	Overall
Argentina	PURE [2]	1.000	0.903	1.000	1.000	1.000	1.000	**0.903**	1.000	0.903	1.000	1.000	1.000	1.000	**0.903**
Australia	1000 Norms Project [3]	1.024	1.000	1.000	1.000	1.023	1.021	**1.070**	1.024	1.000	1.000	1.000	1.016	1.020	**1.061**
	ASPREE [4]	1.000	1.000	1.000	1.000	1.000	1.000	**1.000**	1.000	1.000	1.000	1.000	1.000	1.000	**1.000**
	GOS [5,6][b]	1.000	1.000	1.000	1.000	1.023	1.020	**1.043**	1.000	1.000	1.000	1.000	1.016	1.020	**1.036**
		1.000	1.000	1.000	1.000	1.023	1.000	**1.023**	1.000	1.000	1.000	1.000	1.016	1.000	**1.016**
	Ingram et al. [7]	1.024	1.000	1.000	1.000	1.023	1.021	**1.070**	1.024	1.000	1.000	1.000	1.016	1.020	**1.061**
	NWAHS [8]	1.000	1.000	1.000	1.000	1.023	1.000	**1.023**	1.000	1.000	1.000	1.000	1.016	1.000	**1.016**
	PLSAW [9]	1.000	0.903	1.069	1.000	1.000	1.021	**0.986**	1.000	0.903	1.029	1.000	1.000	1.020	**0.948**
Austria	SHARE [10–16]	1.050	0.903	1.000	1.000	1.000	1.020	**0.967**	1.046	0.903	1.000	1.000	1.000	1.020	**0.963**
Bangladesh	PURE [2]	1.000	0.903	1.000	1.000	1.000	1.000	**0.903**	1.000	0.903	1.000	1.000	1.000	1.000	**0.903**
Barbados	SABE [17,18]	1.050	1.000	1.000	0.995	1.000	1.045	**1.092**	1.046	1.000	1.000	0.974	1.000	1.044	**1.064**
Belgium	SHARE [10–16,19–25]	1.050	0.903	1.000	1.000	1.000	1.020	**0.967**	1.046	0.903	1.000	1.000	1.000	1.020	**0.963**
Brazil	Budziareck et al. [26]	1.000	1.000	1.000	1.000	1.000	1.116	**1.116**	1.000	1.000	1.000	1.000	1.000	1.116	**1.116**
	EDOC [27]	1.000	1.000	1.000	1.000	1.023	1.000	**1.023**	1.000	1.000	1.000	1.000	1.016	1.000	**1.016**
	ELSI-Brazil [28]	1.050	1.000	1.000	1.000	1.000	1.021	**1.072**	1.046	1.000	1.000	1.000	1.000	1.020	**1.067**
	Fernandes et al. [29]	1.000	1.000	1.000	1.000	1.023	1.000	**1.023**	1.000	1.000	1.000	1.000	1.016	1.000	**1.016**
	Fibra-BR [30]	1.000	1.000	1.000	1.000	1.000	1.045	**1.045**	1.000	1.000	1.000	1.000	1.000	1.044	**1.044**
	PNAFS [31]	1.050	0.903	1.069	1.000	1.000	1.000	**1.014**	1.046	0.903	1.029	1.000	1.000	1.000	**0.972**
	PURE [2]	1.000	0.903	1.000	1.000	1.000	1.000	**0.903**	1.000	0.903	1.000	1.000	1.000	1.000	**0.903**

	SABE [17,32]	1.050	1.000	1.000	0.995	1.000	1.045	**1.092**	1.046	1.000	1.000	0.974	1.000	1.044	**1.064**
	Saúde-AC [33]	1.050	1.000	1.000	0.995	1.000	1.045	**1.092**	1.046	1.000	1.000	0.974	1.000	1.044	**1.064**
Bulgaria	SHARE [10,34,35]	1.050	0.903	1.000	1.000	1.000	1.020	**0.967**	1.046	0.903	1.000	1.000	1.000	1.020	**0.963**
Canada	CHMS [36–39]	1.050	0.903	1.069	1.000	1.000	1.020	**1.034**	1.046	0.903	1.029	1.000	1.000	1.020	**0.991**
	CLSA [40]	1.024	1.000	1.000	1.000	1.023	1.021	**1.070**							
	PURE [2]	1.000	0.903	1.000	1.000	1.000	1.000	**0.903**	1.000	0.903	1.000	1.000	1.000	1.000	**0.903**
Chile	Gómez-Campos et al. [41]	1.000	1.000	1.000	1.000	1.000	1.047	**1.047**	1.000	1.000	1.000	1.000	1.000	1.047	**1.047**
	Leal Cárcamo et al. [42]	1.000	1.000	1.000	1.000	1.023	1.116	**1.142**	1.000	1.000	1.000	1.000	1.016	1.116	**1.134**
	PURE [2]	1.000	0.903	1.000	1.000	1.000	1.000	**0.903**	1.000	0.903	1.000	1.000	1.000	1.000	**0.903**
	SABE [17]	1.050	1.000	1.000	0.995	1.000	1.045	**1.092**	1.046	1.000	1.000	0.974	1.000	1.044	**1.064**
China[a]	CHARLS [43,44]	1.050	0.903	1.000	1.000	1.000	1.020	**0.967**	1.046	0.903	1.000	1.000	1.000	1.020	**0.963**
	CNSAF [45]	1.024	0.903	1.069	1.000	1.000	1.045	**1.033**	1.024	0.903	1.029	1.000	1.000	1.044	**0.993**
	PURE [2]	1.000	0.903	1.000	1.000	1.000	1.000	**0.903**	1.000	0.903	1.000	1.000	1.000	1.000	**0.903**
	SAGE [46]	1.050	1.000	1.000	1.000	1.000	1.020	**1.071**	1.046	1.000	1.000	1.000	1.000	1.020	**1.067**
	Shanghai Administration of Sports [47]	1.024	0.903	1.069	1.000	1.000	1.045	**1.033**	1.024	0.903	1.029	1.000	1.000	1.044	**0.993**
Colombia	ENSIN [48]	1.024	0.903	1.069	1.000	1.000	1.020	**1.008**	1.024	0.903	1.029	1.000	1.000	1.020	**0.971**
	PURE [2]	1.000	0.903	1.000	1.000	1.000	1.000	**0.903**	1.000	0.903	1.000	1.000	1.000	1.000	**0.903**
	SABE [49]	1.024	0.903	1.069	1.000	1.000	1.020	**1.008**	1.024	0.903	1.029	1.000	1.000	1.020	**0.971**
Croatia	SHARE [10,24,25,34,35]	1.050	0.903	1.000	1.000	1.000	1.020	**0.967**	1.046	0.903	1.000	1.000	1.000	1.020	**0.963**
Cuba	SABE [17,18]	1.050	1.000	1.000	0.995	1.000	1.045	**1.092**	1.046	1.000	1.000	0.974	1.000	1.044	**1.064**
Cyprus	SHARE [10,34,35]	1.050	0.903	1.000	1.000	1.000	1.020	**0.967**	1.046	0.903	1.000	1.000	1.000	1.020	**0.963**
Czechia	HAPIEE [50]	1.050	0.903	1.000	1.000	1.000	1.020	**0.967**	1.046	0.903	1.000	1.000	1.000	1.020	**0.963**
	SHARE [10,14–16,19–23]	1.050	0.903	1.000	1.000	1.000	1.020	**0.967**	1.046	0.903	1.000	1.000	1.000	1.020	**0.963**
Denmark	DBCS (1905, 1910, 1911, 1915) [51–53][c]	1.050	1.000	1.000	1.000	1.000	1.020	**1.071**	1.046	1.000	1.000	1.000	1.000	1.020	**1.067**
		1.050	0.903	1.000	1.000	1.000	1.020	**0.967**	1.046	0.903	1.000	1.000	1.000	1.020	**0.963**

		1.050	1.000	1.000	1.000	1.000	1.021	**1.072**	1.046	1.000	1.000	1.000	1.000	1.020	**1.067**
		1.050	1.000	1.000	1.000	1.000	1.045	**1.097**	1.046	1.000	1.000	1.000	1.000	1.044	**1.092**
		1.050	0.903	1.000	1.000	1.000	1.021	**0.968**	1.046	0.903	1.000	1.000	1.000	1.020	**0.963**
		1.050	0.903	1.000	1.000	1.000	1.045	**0.991**	1.046	0.903	1.000	1.000	1.000	1.044	**0.986**
	DTR (LSADT, MADT, MIDT) [51,54][c]	1.050	1.000	1.000	1.000	1.000	1.000	**1.050**	1.046	1.000	1.000	1.000	1.000	1.000	**1.046**
		1.050	0.903	1.000	1.000	1.000	1.000	**0.948**	1.046	0.903	1.000	1.000	1.000	1.000	**0.945**
	ECHA [55]	1.050	1.000	1.000	1.000	1.000	1.021	**1.072**	1.046	1.000	1.000	1.000	1.000	1.020	**1.067**
	Health2006 [56]	1.000	1.000	1.000	1.000	1.000	1.021	**1.021**	1.000	1.000	1.000	1.000	1.000	1.020	**1.020**
	SHARE [10–16,19–25]	1.050	0.903	1.000	1.000	1.000	1.020	**0.967**	1.046	0.903	1.000	1.000	1.000	1.020	**0.963**
	Suetta et al. [57]	1.024	1.000	1.000	1.000	1.000	1.021	**1.046**	1.024	1.000	1.000	1.000	1.000	1.020	**1.044**
Ecuador	PURE [2]	1.000	0.903	1.000	1.000	1.000	1.000	**0.903**	1.000	0.903	1.000	1.000	1.000	1.000	**0.903**
Estonia	SHARE [10,14–16,24,25]	1.050	0.903	1.000	1.000	1.000	1.020	**0.967**	1.046	0.903	1.000	1.000	1.000	1.020	**0.963**
Finland	SHARE [10,34,35]	1.050	0.903	1.000	1.000	1.000	1.020	**0.967**	1.046	0.903	1.000	1.000	1.000	1.020	**0.963**
France	ECHA [55]	1.050	1.000	1.000	1.000	1.000	1.021	**1.072**	1.046	1.000	1.000	1.000	1.000	1.020	**1.067**
	Hogrel [58]	1.000	1.000	1.069	1.000	1.000	1.020	**1.090**	1.000	1.000	1.029	1.000	1.000	1.020	**1.050**
	SHARE [10–16,19,20,24,25]	1.050	0.903	1.000	1.000	1.000	1.020	**0.967**	1.046	0.903	1.000	1.000	1.000	1.020	**0.963**
Germany	FORMoSA [59]	1.000	0.903	1.069	1.000	1.000	1.020	**0.985**	1.000	0.903	1.029	1.000	1.000	1.020	**0.948**
	KORA-Age [60]	1.000	0.903	1.000	1.000	1.000	1.021	**0.922**	1.000	0.903	1.000	1.000	1.000	1.020	**0.921**
	NAKO [60]	1.024	1.000	1.000	1.000	1.023	1.000	**1.048**	1.024	1.000	1.000	1.000	1.016	1.000	**1.040**
	OA [61]	1.024	0.903	1.000	1.000	1.000	1.020	**0.943**	1.024	0.903	1.000	1.000	1.000	1.020	**0.943**
	Schilling et al. [62]	1.024	1.000	1.000	1.000	1.023	1.020	**1.069**	1.024	1.000	1.000	1.000	1.016	1.020	**1.061**
	SHARE [10–13,19–23]	1.050	0.903	1.000	1.000	1.000	1.020	**0.967**	1.046	0.903	1.000	1.000	1.000	1.020	**0.963**
	SOEP [63–65]	1.050	0.903	1.000	1.000	1.000	1.020	**0.967**	1.046	0.903	1.000	1.000	1.000	1.020	**0.963**
Ghana	SAGE [46]	1.050	1.000	1.000	1.000	1.000	1.020	**1.071**	1.046	1.000	1.000	1.000	1.000	1.020	**1.067**
Greece	SHARE [10–13,19,20,24,25]	1.050	0.903	1.000	1.000	1.000	1.020	**0.967**	1.046	0.903	1.000	1.000	1.000	1.020	**0.963**

Greenland	Bjerregaard et al. [66]	1.024	1.000	1.000	1.000	1.000	1.000	**1.024**	1.024	1.000	1.000	1.000	1.000	1.000	**1.024**
Hong Kong	Yu et al. [67]	1.024	0.903	1.069	1.000	1.023	1.122	**1.135**							
Hungary	SHARE [10,14–16]	1.050	0.903	1.000	1.000	1.000	1.020	**0.967**	1.046	0.903	1.000	1.000	1.000	1.020	**0.963**
India	LASI [68]	1.050	0.903	1.000	1.000	1.000	1.020	**0.967**	1.046	0.903	1.000	1.000	1.000	1.020	**0.963**
	PURE [2]	1.000	0.903	1.000	1.000	1.000	1.000	**0.903**	1.000	0.903	1.000	1.000	1.000	1.000	**0.903**
	SAGE [46]	1.050	1.000	1.000	1.000	1.000	1.020	**1.071**	1.046	1.000	1.000	1.000	1.000	1.020	**1.067**
	SANSCOG [69]	1.024	1.000	1.000	1.000	1.000	1.020	**1.044**	1.024	1.000	1.000	1.000	1.016	1.020	**1.061**
Iran (Islamic Republic of)	PURE [2]	1.000	0.903	1.000	1.000	1.000	1.000	**0.903**	1.000	0.903	1.000	1.000	1.000	1.000	**0.903**
	Mohammadian et al. [70]	1.000	1.000	1.000	1.000	1.023	1.000	**1.023**	1.000	1.000	1.000	1.000	1.016	1.000	**1.016**
	Rostamzadeh et al. [71]	1.000	1.000	1.000	1.000	1.023	1.000	**1.023**							
Ireland	GenoFit [72]	1.000	0.903	1.069	1.000	1.000	1.020	**0.985**	1.000	0.903	1.029	1.000	1.000	1.020	**0.948**
	SHARE [10,19,20]	1.050	0.903	1.000	1.000	1.000	1.020	**0.967**	1.046	0.903	1.000	1.000	1.000	1.020	**0.963**
	TILDA [73]	1.000	0.903	1.000	1.000	1.000	1.020	**0.921**	1.000	0.903	1.000	1.000	1.000	1.020	**0.921**
Israel	Langer et al. [74]	1.000	1.000	1.000	1.000	1.023	1.000	**1.023**							
	SHARE [10–13,19–23,34,35]	1.050	0.903	1.000	1.000	1.000	1.020	**0.967**	1.046	0.903	1.000	1.000	1.000	1.020	**0.963**
Italy	ECHA [55]	1.050	1.000	1.000	1.000	1.000	1.021	**1.072**	1.046	1.000	1.000	1.000	1.000	1.020	**1.067**
	Lookup 7+ [75]	1.000	1.000	1.000	1.000	1.000	1.067	**1.067**							
	SHARE [10–16,19–25]	1.050	0.903	1.000	1.000	1.000	1.020	**0.967**	1.046	0.903	1.000	1.000	1.000	1.020	**0.963**
Japan	MEXT SPFMA [76–96][d]	1.050	0.903	1.069	1.000	1.000	1.020	**1.034**	1.046	0.903	1.029	1.000	1.000	1.020	**0.991**
		1.050	0.903	1.069	1.000	1.000	1.076	**1.091**	1.046	0.903	1.029	1.000	1.000	1.075	**1.045**
	NILS-LSA [97]	1.024	0.903	1.069	1.000	1.000	1.020	**1.008**	1.024	0.903	1.029	1.000	1.000	1.020	**0.971**
	ROAD [98]	1.050	0.903	1.069	1.000	1.000	1.067	**1.081**	1.046	0.903	1.029	1.000	1.000	1.067	**1.037**
	SONIC [99]	1.050	1.000	1.000	1.000	1.000	1.045	**1.097**	1.046	1.000	1.000	1.000	1.000	1.044	**1.092**
	Taniguchi et al. [100]	1.000	0.903	1.069	1.000	1.000	1.045	**1.009**	1.000	0.903	1.029	1.000	1.000	1.044	**0.970**
	TMIG-LISA (HaCS, ICS, KLS, NCS, YLS) [101]	1.050	0.903	1.069	1.000	1.000	1.104	**1.119**	1.046	0.903	1.029	1.000	1.000	1.104	**1.073**

Kazakhstan	PURE [2]	1.000	0.903	1.000	1.000	1.000	1.000	**0.903**	1.000	0.903	1.000	1.000	1.000	1.000	**0.903**
Kyrgyzstan	PURE [2]	1.000	0.903	1.000	1.000	1.000	1.000	**0.903**	1.000	0.903	1.000	1.000	1.000	1.000	**0.903**
Latvia	SHARE [10,34,35]	1.050	0.903	1.000	1.000	1.000	1.020	**0.967**	1.046	0.903	1.000	1.000	1.000	1.020	**0.963**
Lithuania	HAPIEE [50]	1.050	0.903	1.000	1.000	1.000	1.020	**0.967**	1.046	0.903	1.000	1.000	1.000	1.020	**0.963**
	SHARE [10,34,35]	1.050	0.903	1.000	1.000	1.000	1.020	**0.967**	1.046	0.903	1.000	1.000	1.000	1.020	**0.963**
Luxembourg	SHARE [10,21–25]	1.050	0.903	1.000	1.000	1.000	1.020	**0.967**	1.046	0.903	1.000	1.000	1.000	1.020	**0.963**
Macao	PFSMR [102]	1.024	0.903	1.069	1.000	1.000	1.000	**0.988**	1.024	0.903	1.029	1.000	1.000	1.000	**0.951**
	PFSMR [103–106]	1.024	0.903	1.069	1.000	1.000	1.020	**1.008**	1.024	0.903	1.029	1.000	1.000	1.020	**0.971**
Malaysia	Lam et al. [107]	1.000	1.000	1.000	1.000	1.000	1.086	**1.086**							
	MELoR [108]	1.024	1.000	1.000	1.000	1.000	1.000	**1.024**	1.024	1.000	1.000	1.000	1.000	1.000	**1.024**
	PURE [2]	1.000	0.903	1.000	1.000	1.000	1.000	**0.903**	1.000	0.903	1.000	1.000	1.000	1.000	**0.903**
	Shah et al. [109]	1.050	0.903	1.069	1.000	1.000	1.089	**1.104**	1.046	0.903	1.029	1.000	1.000	1.089	**1.058**
Malta	SHARE [10,34,35]	1.050	0.903	1.000	1.000	1.000	1.020	**0.967**	1.046	0.903	1.000	1.000	1.000	1.020	**0.963**
Mexico	Enríquez-Reyna et al. [110]	1.024	0.903	1.069	1.000	1.000	1.020	**1.008**	1.024	0.903	1.029	1.000	1.000	1.020	**0.971**
	Malina et al. [111]	1.050	0.903	1.069	1.000	1.000	1.000	**1.014**	1.046	0.903	1.029	1.000	1.000	1.000	**0.972**
	Núñez-Othón et al. [112]	1.000	0.903	1.069	1.000	1.000	1.020	**0.985**	1.000	0.903	1.029	1.000	1.000	1.020	**0.948**
	Rodríguez-García et al. [113,114]	1.050	0.903	1.069	1.000	1.000	1.000	**1.014**	1.046	0.903	1.029	1.000	1.000	1.000	**0.972**
	SABE [17]	1.050	1.000	1.000	0.995	1.000	1.045	**1.092**	1.046	1.000	1.000	0.974	1.000	1.044	**1.064**
	SAGE [46]	1.050	1.000	1.000	1.000	1.000	1.020	**1.071**	1.046	1.000	1.000	1.000	1.000	1.020	**1.067**
Nepal	Bimali et al. [115]	1.000	1.000	1.000	1.000	1.023	1.000	**1.023**							
	Kim et al. [116]	1.024	0.903	1.069	1.000	1.023	1.089	**1.101**	1.024	0.903	1.029	1.000	1.016	1.089	**1.053**
Netherlands	SHARE [10–16,19–23]	1.050	0.903	1.000	1.000	1.000	1.020	**0.967**	1.046	0.903	1.000	1.000	1.000	1.020	**0.963**
Norway	FYSIOPRIM [117]	1.000	1.000	1.000	1.000	1.023	1.020	**1.043**	1.000	1.000	1.000	1.000	1.016	1.020	**1.036**
	Kjær et al. [118]	1.000	0.903	1.069	1.000	1.000	1.021	**0.986**	1.000	0.903	1.029	1.000	1.000	1.020	**0.948**
	Nilsen et al. [119]	1.024	1.000	1.000	1.000	1.023	1.107	**1.160**							

	Tromsø [120]	1.024	1.000	1.000	1.000	1.023	1.000	**1.048**	1.024	1.000	1.000	1.000	1.016	1.000	**1.040**
Pakistan	PURE [2]	1.000	0.903	1.000	1.000	1.000	1.000	**0.903**	1.000	0.903	1.000	1.000	1.000	1.000	**0.903**
Philippines	Afable et al. [121]	1.050	0.903	1.069	1.000	1.000	1.000	**1.014**	1.046	0.903	1.029	1.000	1.000	1.000	**0.972**
	PURE [2]	1.000	0.903	1.000	1.000	1.000	1.000	**0.903**	1.000	0.903	1.000	1.000	1.000	1.000	**0.903**
Poland	HAPIEE [50]	1.050	0.903	1.000	1.000	1.000	1.020	**0.967**	1.046	0.903	1.000	1.000	1.000	1.020	**0.963**
	PURE [2]	1.000	0.903	1.000	1.000	1.000	1.000	**0.903**	1.000	0.903	1.000	1.000	1.000	1.000	**0.903**
	SHARE [10,19,20,24,25,34,35]	1.050	0.903	1.000	1.000	1.000	1.020	**0.967**	1.046	0.903	1.000	1.000	1.000	1.020	**0.963**
	Wiśniowska-Szurlej et al. [122]	1.024	1.000	1.000	1.000	1.023	1.082	**1.133**	1.024	1.000	1.000	1.000	1.016	1.082	**1.126**
Portugal	NUP65 [123]	1.024	1.000	1.000	1.000	1.023	1.085	**1.137**	1.024	1.000	1.000	1.000	1.016	1.085	**1.129**
	SHARE [10,14–16]	1.050	0.903	1.000	1.000	1.000	1.020	**0.967**	1.046	0.903	1.000	1.000	1.000	1.020	**0.963**
Republic of Korea	KNHANES [124,125]	1.024	0.903	1.069	1.000	1.000	1.000	**0.988**	1.024	0.903	1.029	1.000	1.000	1.000	**0.951**
	MCST NSPF [126–130]	1.050	0.903	1.069	1.000	1.000	1.020	**1.034**	1.046	0.903	1.029	1.000	1.000	1.020	**0.991**
Romania	SHARE [10,34,35]	1.050	0.903	1.000	1.000	1.000	1.020	**0.967**	1.046	0.903	1.000	1.000	1.000	1.020	**0.963**
Russian Federation	KYH [131]	1.024	1.000	1.000	1.000	1.023	1.000	**1.048**	1.024	1.000	1.000	1.000	1.016	1.000	**1.040**
	PURE [2]	1.000	0.903	1.000	1.000	1.000	1.000	**0.903**	1.000	0.903	1.000	1.000	1.000	1.000	**0.903**
	SAGE [46]	1.050	1.000	1.000	1.000	1.000	1.020	**1.071**	1.046	1.000	1.000	1.000	1.000	1.020	**1.067**
	SAHR [131]	1.050	0.903	1.000	1.000	1.000	1.000	**0.948**	1.046	0.903	1.000	1.000	1.000	1.000	**0.945**
	Turusheva et al. [133]	1.024	1.000	1.000	1.000	1.000	1.000	**1.024**	1.024	1.000	1.000	1.000	1.000	1.000	**1.024**
Saudi Arabia	Alqahtani et al. [134]	1.024	0.903	1.069	1.000	1.023	1.000	**1.011**	1.024	0.903	1.029	1.000	1.016	1.000	**0.967**
	Alrashdan et al. [135]	1.000	1.000	1.000	1.000	1.023	1.000	**1.023**	1.000	1.000	1.000	1.000	1.016	1.000	**1.016**
	PURE [2]	1.000	0.903	1.000	1.000	1.000	1.000	**0.903**	1.000	0.903	1.000	1.000	1.000	1.000	**0.903**
Singapore	SIHLS [136]	1.050	0.903	1.069	1.000	1.000	1.020	**1.034**	1.046	0.903	1.029	1.000	1.000	1.020	**0.991**
	WiSE [137]	1.024	1.000	1.000	1.000	1.023	1.045	**1.095**	1.024	1.000	1.000	1.000	1.016	1.044	**1.086**
	Yishun [138]	1.024	1.000	1.000	1.000	1.000	1.020	**1.044**	1.024	1.000	1.000	1.000	1.000	1.020	**1.044**
Slovakia	SHARE [10,34,35]	1.050	0.903	1.000	1.000	1.000	1.020	**0.967**	1.046	0.903	1.000	1.000	1.000	1.020	**0.963**

Slovenia	SHARE [10,14–16,21–25]	1.050	0.903	1.000	1.000	1.000	1.020	**0.967**	1.046	0.903	1.000	1.000	1.000	1.020	**0.963**
South Africa	PURE [2]	1.000	0.903	1.000	1.000	1.000	1.000	**0.903**	1.000	0.903	1.000	1.000	1.000	1.000	**0.903**
	SAGE [46]	1.050	1.000	1.000	1.000	1.000	1.020	**1.071**	1.046	1.000	1.000	1.000	1.000	1.020	**1.067**
Spain	Pizzara [139]	1.000	1.000	1.000	1.000	1.000	1.021	**1.021**	1.000	1.000	1.000	1.000	1.000	1.020	**1.020**
	SHARE [10–16,19–23]	1.050	0.903	1.000	1.000	1.000	1.020	**0.967**	1.046	0.903	1.000	1.000	1.000	1.020	**0.963**
State of Palestine	PURE [2]	1.000	0.903	1.000	1.000	1.000	1.000	**0.903**	1.000	0.903	1.000	1.000	1.000	1.000	**0.903**
Sweden	Axelsson et al. [140]	1.000	0.903	1.000	1.000	1.023	1.067	**0.986**	1.000	0.903	1.000	1.000	1.016	1.067	**0.979**
	MrOS [141]	1.000	1.000	1.000	1.000	1.000	1.047	**1.047**	1.000	1.000	1.000	1.000	1.000	1.047	**1.047**
	PURE [2]	1.000	0.903	1.000	1.000	1.000	1.000	**0.903**	1.000	0.903	1.000	1.000	1.000	1.000	**0.903**
	SHARE [10–13,19–23]	1.050	0.903	1.000	1.000	1.000	1.020	**0.967**	1.046	0.903	1.000	1.000	1.000	1.020	**0.963**
Switzerland	COmPLETE [142]	1.024	0.903	1.069	1.000	1.000	1.021	**1.009**	1.024	0.903	1.029	1.000	1.000	1.020	**0.971**
	SHARE [10–16,19,20]	1.050	0.903	1.000	1.000	1.000	1.020	**0.967**	1.046	0.903	1.000	1.000	1.000	1.020	**0.963**
	Wearing et al. [143]	1.000	1.000	1.000	1.000	1.023	1.000	**1.023**	1.000	1.000	1.000	1.000	1.016	1.000	**1.016**
	Werle et al. [144]	1.000	1.000	1.000	1.000	1.023	1.082	**1.107**							
Taiwan	ILAS [145]	1.050	0.903	1.069	1.000	1.000	1.021	**1.035**	1.046	0.903	1.029	1.000	1.000	1.020	**0.991**
Türkiye	PURE [2]	1.000	0.903	1.000	1.000	1.000	1.000	**0.903**	1.000	0.903	1.000	1.000	1.000	1.000	**0.903**
United Arab Emirates	PURE [2]	1.000	0.903	1.000	1.000	1.000	1.000	**0.903**	1.000	0.903	1.000	1.000	1.000	1.000	**0.903**
United Kingdom	ELSA [146,147][c]	1.050	1.000	1.000	1.000	1.000	1.000	**1.050**	1.046	1.000	1.000	1.000	1.000	1.000	**1.046**
		1.050	0.903	1.000	1.000	1.000	1.000	**0.948**	1.046	0.903	1.000	1.000	1.000	1.000	**0.945**
	EPIC-Norfolk [148]	1.050	0.903	1.000	1.000	1.000	1.020	**0.967**	1.046	0.903	1.000	1.000	1.000	1.020	**0.963**
	HCS [149]	1.000	1.000	1.000	1.000	1.000	1.000	**1.000**	1.000	1.000	1.000	1.000	1.000	1.000	**1.000**
	Me, Human [150]	1.000	1.000	1.000	1.000	1.000	1.020	**1.020**							
	Twenty-07 [151]	1.000	0.903	1.000	1.000	1.000	1.000	**0.903**	1.000	0.903	1.000	1.000	1.000	1.000	**0.903**
	UKHLS [152][c]	1.050	1.000	1.000	1.000	1.000	1.000	**1.050**	1.046	1.000	1.000	1.000	1.000	1.000	**1.046**
		1.050	0.903	1.000	1.000	1.000	1.000	**0.948**	1.046	0.903	1.000	1.000	1.000	1.000	**0.945**

	Wozny et al. [153]	1.000	1.000	1.000	1.000	1.023	1.086	**1.111**							
United Republic of Tanzania	PURE [2]	1.000	0.903	1.000	1.000	1.000	1.000	**0.903**	1.000	0.903	1.000	1.000	1.000	1.000	**0.903**
United States of America	ASPREE [4]	1.000	1.000	1.000	1.000	1.000	1.000	**1.000**	1.000	1.000	1.000	1.000	1.000	1.000	**1.000**
	HRS [154,155]	1.050	0.903	1.000	1.000	1.000	1.020	**0.967**	1.046	0.903	1.000	1.000	1.000	1.020	**0.963**
	MSK-FIT [156]	1.024	1.000	1.000	1.000	1.023	1.021	**1.070**	1.024	1.000	1.000	1.000	1.016	1.020	**1.061**
	NHANES [157]	1.024	0.903	1.069	1.000	1.000	1.000	**0.988**	1.024	0.903	1.029	1.000	1.000	1.000	**0.951**
	NIH Toolbox [158,159]	1.000	1.000	1.000	1.000	1.023	1.067	**1.092**	1.000	1.000	1.000	1.000	1.016	1.067	**1.084**
Uruguay	SABE [17]	1.050	1.000	1.000	0.995	1.000	1.045	**1.092**	1.046	1.000	1.000	0.974	1.000	1.044	**1.064**
Zimbabwe	PURE [2]	1.000	0.903	1.000	1.000	1.000	1.000	**0.903**	1.000	0.903	1.000	1.000	1.000	1.000	**0.903**

Notes: Adjustments correspond to the test protocols in Table 4B. HGS data (means and SDs) were adjusted to the common metric by multiplying these values by the overall adjustment factor.

[a] China refers to the mainland of China;

[b] Raw HGS data were pre-adjusted for dynamometer by the authors before the descriptive data were shared, hence why no additional adjustment was made for dynamometer;

[c] Because raw HGS data were available and body position, testing hand, and reps per hand were coded at the individual-level, combinations of adjustment factors were separated for completeness (see Supplementary Table 4B);

[d] Separate adjustments for HGS calculated as the maximum value irrespective of hand (first row, for raw data from 2013–2019) and as the maximum values for each hand (second row, for descriptive data from 2000–12) are shown (see Supplementary Table 4B).

Abbreviations: HGS = handgrip strength; RU = Radioulnar. Studies: ASPREE = ASPirin in Reducing Events in the Elderly study; CHARLS = China Health and Retirement Longitudinal Study; CHMS = Canadian Health Measures Survey; CLSA = Canadian Longitudinal Study on Ageing; CNFAS = Chinese National Survey on Adults' Fitness; COmPLETE = CardiO-PuLmonary Exercise Testing health study; DBCS = Danish Birth Cohort Studies of 1905, 1910, 1911, and 1915; DTR (LSADT, MADT, MIDT) = Danish Twin Registry (Longitudinal Study of Aging Danish Twins, Middle Age Danish Twins, MIddle age Danish Twins); ECHA = European Challenge for Healthy Aging study; EDOC = Estudo das Doenças Crônicas (English translation: Study of Chronic Diseases); ELSA = English Longitudinal Study of Ageing; ELSI-Brazil = Brazilian Longitudinal Study of Aging; ENSIN = Encuesta Nacional de la Situacion Nutricional en Colombia (English translation: 2015 National Nutritional Survey); EPIC-Norfolk = European Prospective Investigation into Cancer-Norfolk study; Fibra-BR = Frailty in Brazilian Older People study; FORMoSA = Bavarian Research Foundation-Sarcopenia and Osteoporosis study; FYSIOPRIM = Research Program for Physiotherapy in Primary Health Care study; GenoFit = GenoFit study; GOS = Geelong Osteoporosis Study; HAPIEE = Health, Alcohol and Psychosocial factors In Eastern Europe study; HCS = Hertfordshire Cohort Study; Health2006 = Health2006 study; HRS = Health and Retirement Study; ILAS = I-Lan Longitudinal Aging Study; KNHANES = Korea National Health and Nutrition Examination Survey; KORA-Age = Kooperative Gesundheitsforschung in der Region Augsburg (English translation: Cooperative Health Research in the Region of Augsburg study); KYH = Know Your Heart study; LASI = Longitudinal Ageing Study in India; Lookup 7+ = Longevity check-up 7+ study; MCST NSPF = Ministry of Culture, Sports and Tourism's National Survey of Physical Fitness; Me, Human = Me, Human project; MELoR = Malaysian Elders Longitudinal Research study; MEXT SPFMA = Ministry of Education, Culture, Sports, Science and Technology's Survey on Physical Fitness and Motor Abilities; MrOS = Osteoporotic Fractures in Men (Sweden cohort) study; MSK-FIT = Musculoskeletal, Function, Imaging, and Tissue Resource Core; NAKO = NAKO Gesundheitsstudie (English translation: German National Cohort); NHANES = National Health and Nutrition Examination Survey; NIH Toolbox = U.S. National Institutes of Health Toolbox study; NILS-LSA = National Institute for Longevity Sciences-Longitudinal Study of Aging; NUP65 = Nutrition UP 65 study; NWAHS = North West Adelaide Health Study; OA = Outdoor Active study; Pizzara = Pizzara study; PFSMR = Physical Fitness Study of Macao SAR Residents; PLSAW = Perth Longitudinal Study of Ageing Women; PNAFS = Nutrition, Physical Activity and Health Survey; PURE = Prospective Urban Rural Epidemiology study; ROAD = Research on Osteoarthritis/Osteoporosis Against Disability study; SABE = SAlud, Bienestar, y Envejecimiento (English

translation: Survey on Health, Well-Being, and Aging in Latin America and the Caribbean); SAGE = World Health Organization Study on global AGEing and adult health; SAHR = Study of Stress, Aging, and Health in Russia; SANSCOG = Srinivaspura NeuoSenescence, and COGnition study; Saúde-AC = English translation: Health-Antônio Carlos (Santa Catarina) study; SHARE = Survey of Health, Ageing and Retirement in Europe; SIHLS = Social Isolation, Health, and Lifestyles Survey; SONIC = Septuagenarians, Octogenarians, Nonagenarians Investigation with Centenarians study; SOEP = German Socio-Economic Panel survey; TILDA = The Irish Longitudinal Study on Ageing; TMIG-LISA (HaCS, ICS, KLS, NCS, YLS) = Tokyo Metropolitan Institute of Gerontology-Longitudinal Interdisciplinary Study on Aging (Hatoyama Cohort Study, Itabashi Cohort Studies of 2002 and 2011, Kusatsu Longitudinal Study, Nangai Cohort Study, Yoita Longitudinal Study); Tromsø = Norwegian Tromsø study; Twenty-07 = West of Scotland Twenty-07 study; UKHLS = Understanding Society: The UK Household Longitudinal Study; WiSE = Well-being of Singapore Elderly study; Yishun = Yishun study.

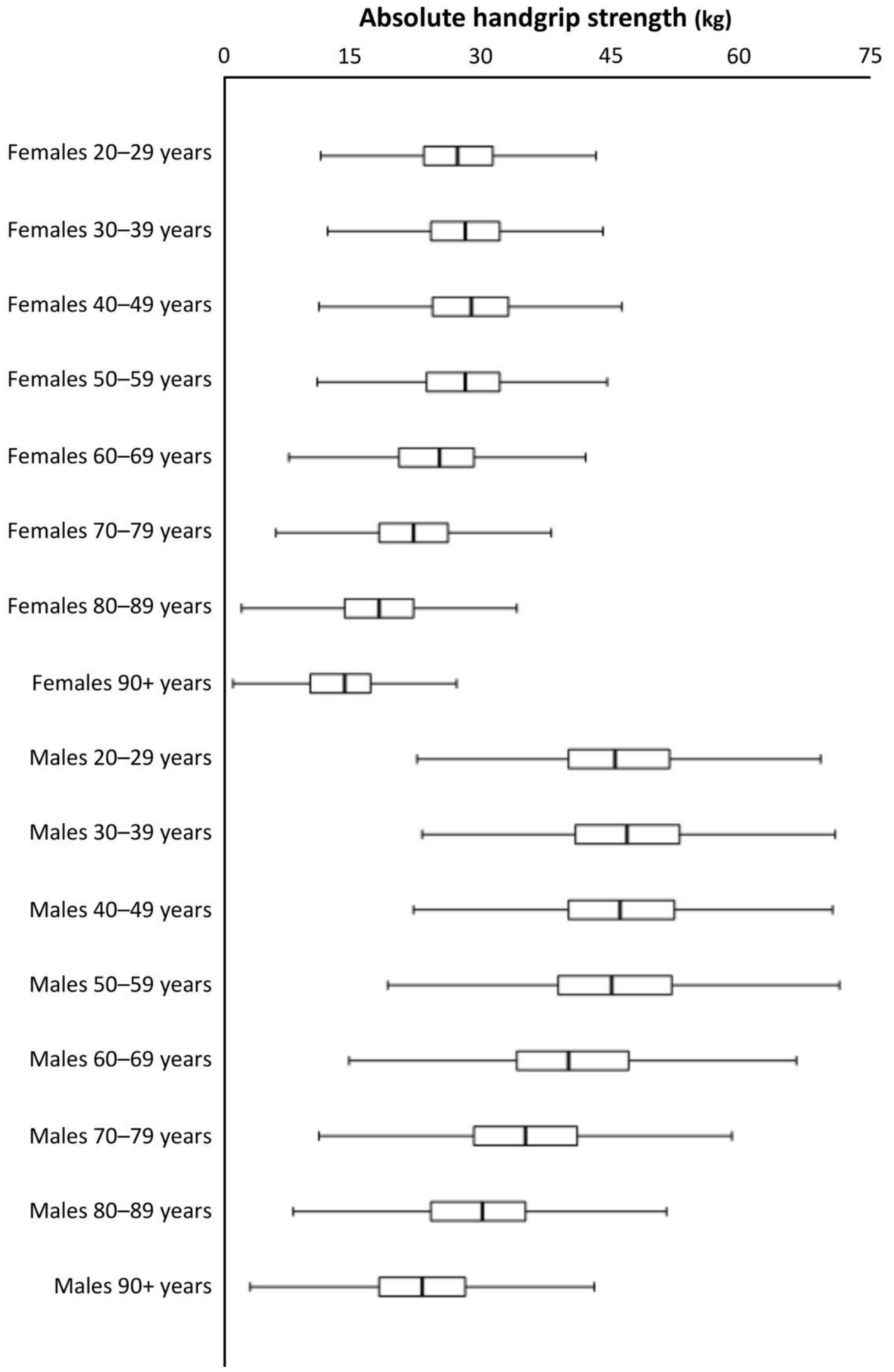

Supplementary Fig 1A. Sex- and age-specific box plots for absolute handgrip strength (in kilograms (kg)) generated from raw data (*n* = 366,367).

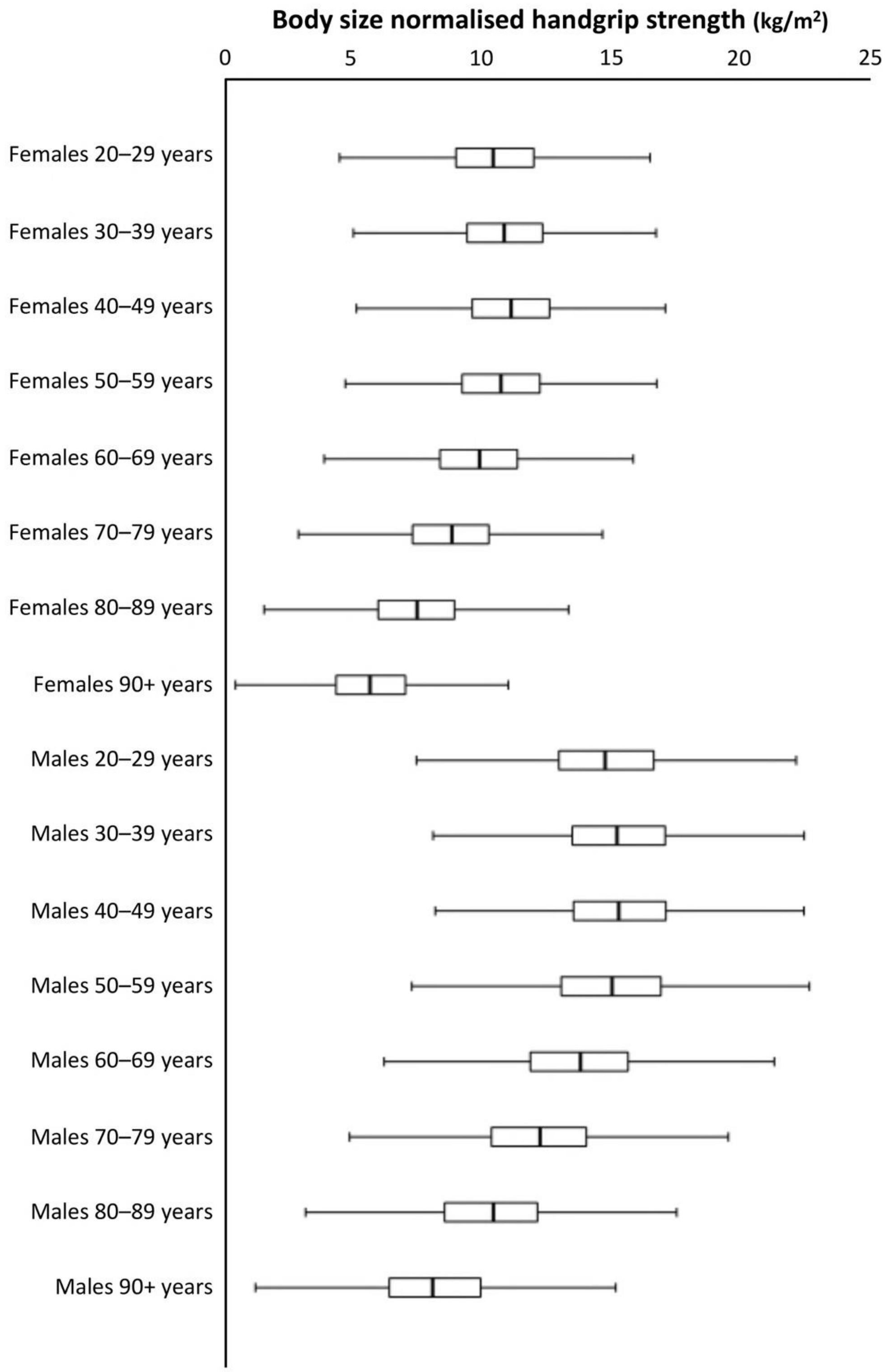

Supplementary Fig. 1B. Sex- and age-specific box plots for normalized handgrip strength (handgrip strength in kilograms divided by height in meters squared (kg/m^2)) generated from raw data (*n* = 357,063).

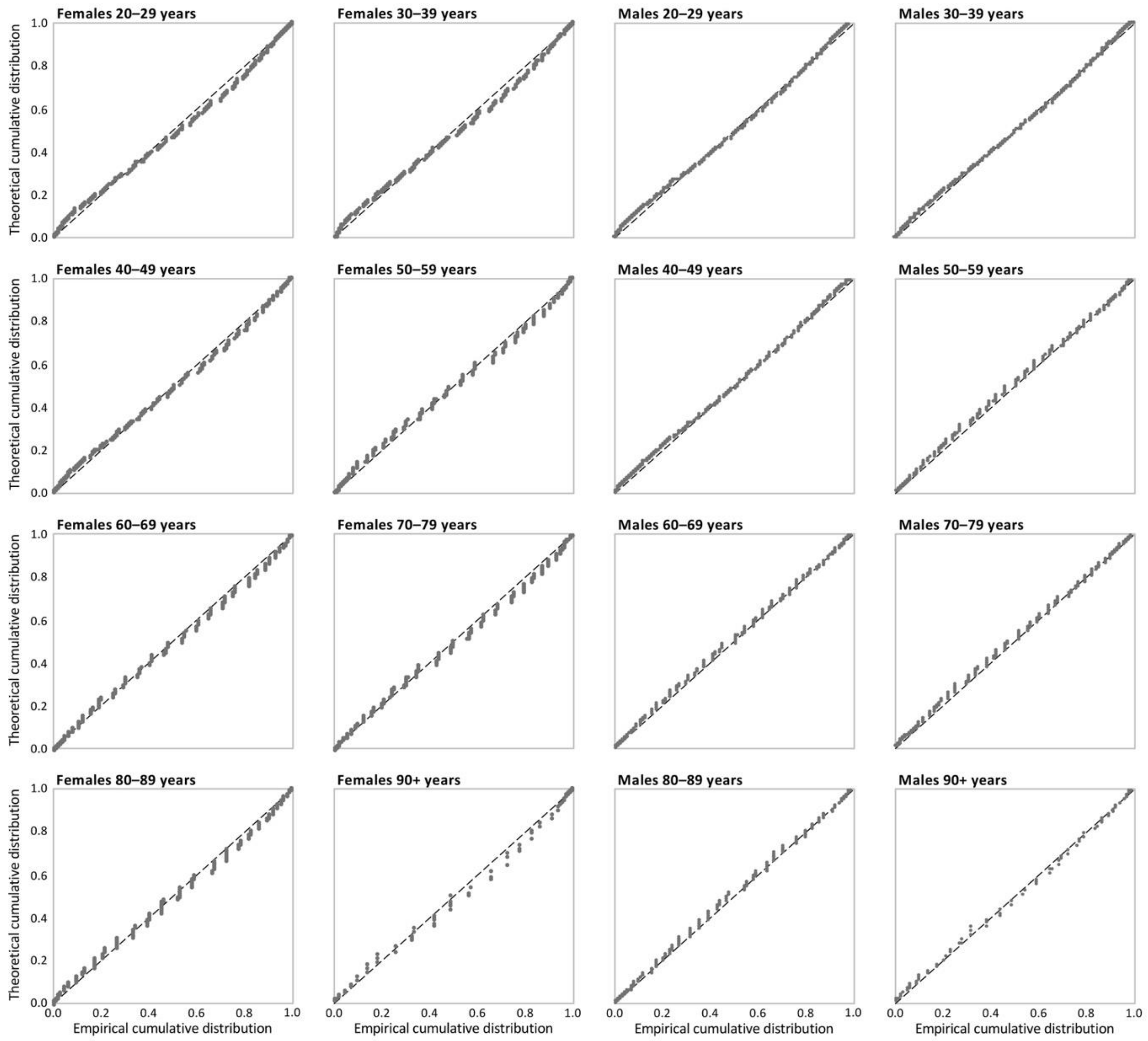

Supplementary Fig. 1C. Sex- and age-specific probability plots for absolute handgrip strength (in kilograms) generated from raw data (*n* = 366,367).

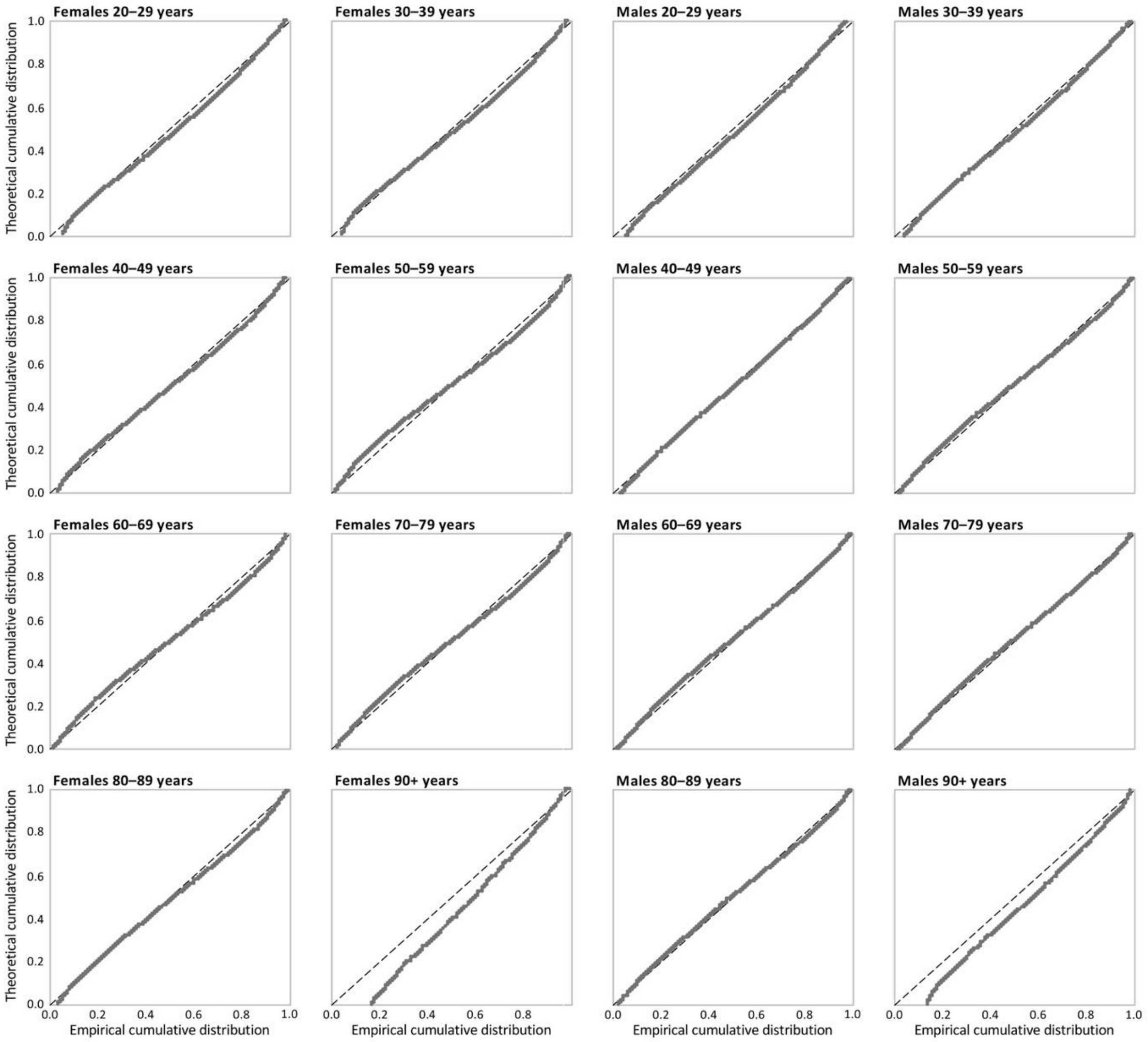

Supplementary Fig. 1D. Sex- and age-specific probability plots for normalized handgrip strength (handgrip strength in kilograms divided by height in meters squared (kg/m^2)) generated from raw data (*n* = 357,063).

Supplementary Notes and Abbreviations. Additional notes and abbreviations for Supplementary Table 1.

Notes: The GOS [5,6] used both hydraulic and electronic dynamometers for females and males, respectively. However, for this summary we counted dynamometer only as *hydraulic* because raw HGS data were pre-adjusted for dynamometer (by estimating hydraulic values from electronic values) before the descriptive data were shared; 11 studies (DBCS (1905, 1910, 1911, 1915) [51–53]; DTR (LSADT, MADT, MIDT) [51,54]; ELSA [146]; EPIC-Norfolk [148]; HRS [154,155]; LASI [67]; SAHR [132]; SHARE [10–16,19–25,34,35]; Taniguchi et al. [100]; TILDA [73]; UKHLS [152]) conducted HGS testing in a standing (preferred)/seated body position, which counted as *standing* for this summary; the GOS [5,6] provided no verbal or verbal support for females and males, respectively. However, for this summary we counted verbal support only as *no* because it was the more common protocol; The DBCS (1905, 1910, 1911, 1915) [51–53] used each hand or only the dominant hand. For this summary, we counted testing hand as *both* because the sample size was substantially larger for those who used each hand; Because 2–3 or 2+ reps per hand were performed for 6 studies (DBCS (1905, 1910, 1911, 1915) [51–53]; GOS [5,6]; Hogrel [58]; PFSMR [102–106]; ENSIN [48]; SABE [49]), for this summary we counted the reps per hand as the more common protocol; for the MEXT SPFMA [76–96], because descriptive data were available and HGS was calculated as the average of maxima (i.e., the average of the maximum values for each hand) between 2000 and 2012, and raw data were available and HGS was calculated as the maximum irrespective of hand from 2013–19 onwards, for this summary we counted summary statistic as the *average of maxima* as it was the more common protocol.

Abbreviations: DBCS = Danish Birth Cohort Studies of 1905, 1910, 1911, and 1915; DTR (LSADT, MADT, MIDT) = Danish Twin Registry (Longitudinal Study of Aging Danish Twins, Middle Age Danish Twins, MIddle age Danish Twins); ELSA = English Longitudinal Study of Ageing; ENSIN = Encuesta Nacional de la Situacion Nutricional en Colombia (English translation: 2015 National Nutritional Survey); EPIC-Norfolk = European Prospective Investigation into Cancer-Norfolk study; GOS = Geelong Osteoporosis Study; HRS = Health and Retirement Study; LASI = Longitudinal Ageing Study in India; MEXT SPFMA = Ministry of Education, Culture, Sports, Science and Technology’s Survey on Physical Fitness and Motor Abilities; PFSMR = Physical Fitness Study of Macao SAR Residents; SABE = SAlud, Bienestar, y Envejecimiento (English translation: Survey on Health, Well-Being, and Aging in Latin America and the Caribbean); SAHR = Study of Stress, Aging, and Health in Russia; SHARE = Survey of Health, Ageing and Retirement in

Europe; TILDA = The Irish Longitudinal Study on Ageing; UKHLS = Understanding Society: The UK Household Longitudinal Study.

Supplementary Table 4A. Descriptive characteristics of the included studies by country or region.

Country or region	Study	Year of testing	Age (years)	Sample size	Sex	Study design	Sampling strategy	Sample base	HDI	Absolute HGS	Normalized HGS
Argentina	PURE [2]	2003–09	35–69	7462	F (61.5%)<br>M (38.5%)	Cohort	Probability/<br>Non-probability	Non-national	0.842<br>(Very high)	•	•
Australia	1000 Norms Project [3]	2014–15	20–80+	700	F (50.0%)<br>M (50.0%)	Cross-sectional	Non-probability	Non-national	0.951<br>(Very high)	•	•
	ASPREE [4]	2010–14	70–85+	16,534	F (54.9%)<br>M (45.1%)	Cohort	Non-probability	Non-national		•	•
	GOS [5,6]	2011–14	30–85+	746	F (100%)	Cohort	Probability	Non-national		•	•
		2016–20	30–85+	581	M (100%)					•	•
	Ingram et al. [7]	2016–17	20–85+	366	F (53.0%)<br>M (47.0%)	Cross-sectional	Non-probability	Non-national		•	•
	NWAHS [8]	2004–06	20–94	3139	F (50.6%)<br>M (49.4%)	Cohort	Probability	Non-national		•	•
	PLSAW [9]	2003	75–89	1163	F (100%)	Cohort	Non-probability	Non-national		•	•
Austria	SHARE [10–16]	2004–06<br>2010–12	50–80+	4968	F (57.1%)<br>M (42.9%)	Cohort	Probability	National	0.916<br>(Very high)	•	•
Bangladesh	PURE [2]	2003–09	35–69	2712	F (54.6%)<br>M (45.4%)	Cohort	Probability/<br>Non-probability	Non-national	0.661<br>(Medium)	•	•
Barbados	SABE [17,18]	1999–2000	60–85+	1417	F (60.8%)<br>M (39.2%)	Cross-sectional	Probability	Non-national	0.79<br>(High)	•	•
Belgium	SHARE [10–16,19–25]	2004–06<br>2006–10<br>2010–12<br>2013<br>2015	50–80+	8617	F (52.6%)<br>M (47.4%)	Cohort	Probability	National	0.937<br>(Very high)	•	•
Brazil	Budziareck et al. [26]	2006	20–79	300	F (50.0%)<br>M (50.0%)	Cross-sectional	Non-probability	Non-national	0.754<br>(High)	•	•
	EDOC [27]	2014	20–85+	1425	F (61.8%)<br>M (38.2%)	Cross-sectional	Probability	Non-national		•	•
	ELSI-Brazil [28]	2019–21	50–85+	7905	F (60.0%)<br>M (40.0%)	Cohort	Probability	National		•	•
	Fernandes et al. [29]	2004	20–59	848	M (100%)	Cross-sectional	Probability	Non-national		•	•

	Fibra-BR [30]	2009–10	65–90+	6656	F (66.8%)<br>M (33.2%)	Cross-sectional	Probability	National		•	•
	PNAFS [31]	2003	20–70+	3050	F (63.2%)<br>M (36.8%)	Cross-sectional	Probability	Non-national		•	•
	PURE [2]	2003–09	35–69	5575	F (55.3%)<br>M (44.7%)	Cohort	Probability/<br>Non-probability	Non-national		•	•
	SABE [17,32]	2000–01	60–85+	1850	F (59.1%)<br>M (40.9%)	Cross-sectional	Probability	Non-national		•	•
	Saúde-AC [33]	2010	60–85+	460	F (57.2%)<br>M (42.8%)	Cross-sectional	Probability	Non-national		•	•
Bulgaria	SHARE [10,34,35]	2017	50–80+	1774	F (56.9%)<br>M (43.1%)	Cohort	Probability	National	0.795<br>(High)	•	•
Canada	CHMS [36–39]	2007–09<br>2009–11<br>2012–13<br>2014–15<br>2016–17<br>2018–19	20–79	19,372	F (51.5%)<br>M (48.5%)	Cross-sectional	Probability	National	0.936<br>Very high)	•	•
	CLSA [40]	2011–15	45–85	20,290	F (49.5%)<br>M (50.5%)	Cohort	Probability	National		•	
	PURE [2]	2003–09	35–69	10,011	F (53.6%)<br>M (46.4%)	Cohort	Probability/<br>Non-probability	Non-national		•	•
Chile	Gómez-Campos et al. [41]	2015–18	20–80	1690	F (66.2%)<br>M (33.8%)	Cross-sectional	Probability	Non-national	0.855<br>(Very high)	•	•[c]
	Leal Cárcamo et al. [42]	2013	20–69	401	F (50.6%)<br>M (49.4%)	Cross-sectional	Non-probability	Non-national		•	•
	PURE [2]	2003–09	35–69	3218	F (65.4%)<br>M (34.6%)	Cohort	Probability/<br>Non-probability	Non-national		•	•
	SABE [17]	1999–2000	60–85+	1220	F (66.1%)<br>M (33.9%)	Cross-sectional	Probability	Non-national		•	•
China[a]	CHARLS [43,44]	2013	45–85+	10,246	F (52.5%)<br>M (47.5%)	Cohort	Probability	National	0.768<br>(High)	•	•
	CNSAF [45]	2000<br>2005<br>2010<br>2014	20–69	719,885	F (50.0%)<br>M (50.0%)	Cross-sectional	Probability	National		•	•[c]

	PURE [2]	2003–09	35–69	46,966	F (58.5%)<br>M (41.5%)	Cohort	Probability/<br>Non-probability	Non-national		•	•
	SAGE [46]	2007–10	20–85+	13,389	F (52.1%)<br>M (47.9%)	Cohort	Probability	National		•	•
	Shanghai Administration of Sports [47]	2020	20–59	26,652	F (50.1%)<br>M (49.9%)	Cross-sectional	Probability	Non-national		•	•
Colombia	ENSIN [48]	2015	20–64	1026	F (43.9%)<br>M (56.1%)	Cross-sectional	Probability	National		•	•
	PURE [2]	2003–09	35–69	7483	F (64.1%)<br>M (35.9%)	Cohort	Probability/<br>Non-probability	Non-national	0.752<br>High)	•	•
	SABE [49]	2015	60–90+	4784	F (57.9%)<br>M (42.1%)	Cross-sectional	Probability	National		•	•
Croatia	SHARE [10,24,25,34,35]	2015<br>2017	50–80+	2533	F (54.8%)<br>M (45.2%)	Cohort	Probability	National	0.858<br>(Very high)	•	•
Cuba	SABE [17,18]	1999–2000	60–85+	1693	F (62.5%)<br>M (37.5%)	Cross-sectional	Probability	Non-national	0.764<br>(High)	•	•
Cyprus	SHARE [10,34,35]	2017	50–80+	1101	F (57.8%)<br>M (42.2%)	Cohort	Probability	National	0.896<br>(Very high)	•	•
Czechia	HAPIEE [50]	2002–05	45–74	5225	F (54.5%)<br>M (45.5%)	Cohort	Probability	Non-national	0.889<br>(Very high)	•	•
	SHARE [10,14–16,19–23]	2006–10<br>2010–12<br>2013	50–80+	7323	F (56.6%)<br>M (43.4%)	Cohort	Probability	National		•	•
Denmark	DBCS (1905, 1910, 1911, 1915) [51–53]<br><br>DTR (LSADT, MADT, MIDT) [51,54]	2000<br>2010<br>2011<br>2001<br>2008–11	30–100+	16,989	F (54.9%)<br>M (45.1%)	Cohort	Probability	National	0.948<br>(Very high)	•	•
	ECHA [55]	2002–04	40–100+	524	F (41.8%)<br>M (58.2%)	Cross-sectional	Non-probability	Non-national		•	•
	Health2006 [56]	2006–08	20–72	3426	F (55.2%)<br>M (44.8%)	Cross-sectional	Probability	Non-national		•	•
	SHARE [10–16,19–25]	2004–06<br>2006–10<br>2010–12<br>2013	50–80+	5176	F (52.5%)<br>M (47.5%)	Cohort	Probability	National		•	•

		2015								
	Suetta et al. [57]	2013–16	21–92	1236	F (55.4%)<br>M (44.6%)	Cross-sectional	Probability	Non-national		•	•
Ecuador	PURE [2]	2003–09	35–69	2026	F (72.1%)<br>M (27.9%)	Cohort	Probability/<br>Non-probability	Non-national	0.740<br>(High)	•	•
Estonia	SHARE [10,14–16,24,25]	2010–12<br>2015	50–80+	6550	F (60.1%)<br>M (39.9%)	Cohort	Probability	National	0.890<br>(Very high)	•	•
Finland	SHARE [10,34,35]	2017	50–80+	1924	F (53.2%)<br>M (46.8%)	Cohort	Probability	National	0.940<br>(Very high)	•	•
France	ECHA [55]	2002–04	40–100+	272	F (57.0%)<br>M (43.0%)	Cross-sectional	Non-probability	Non-national	0.903<br>(Very high)	•	•
	Hogrel [58]	2006–08	20–79	274	F (59.1%)<br>M (40.9%)	Cross-sectional	Non-probability	Non-national		•	•
	SHARE [10–16,19,20,24,25]	2004–06<br>2006–10<br>2010–12<br>2015	50–80+	6793	F (54.6%)<br>M (45.4%)	Cohort	Probability	National		•	•
Germany	FORMoSA [59]	2014	70–95	2288	F (57.8%)<br>M (42.2%)	Cross-sectional	Non-probability	Non-National	0.942<br>(Very high)	•	•
	KORA-Age [60]	2008–09	65–89	1045	F (49.9%)<br>M (50.1%)	Cohort	Probability	Non-national		•	•
	NAKO [60]	2014–19	20–74	200,377	F (50.2%)<br>M (49.8%)	Cohort	Probability	National		•	•
	OA [61]	2018–21	65–75	1647	F (53.2%)<br>M (46.8%)	Cohort	Probability	Non-national		•	•
	Schilling et al. [62]	2021	35–89	388	F (53.6%)<br>M (46.4%)	Cohort	Probability	Non-national		•	•
	SHARE [10–13,19–23]	2004–06<br>2006–10<br>2013	50–80+	7609	F (51.9%)<br>M (48.1%)	Cohort	Probability	National		•	•
	SOEP [63–65]	2006<br>2008<br>2010<br>2012<br>2014<br>2016<br>2018	20–80+	14,123	F (52.8%)<br>M (47.2%)	Cohort	Probability	National		•	•

Ghana	SAGE [46]	2007–08	20–85+	4480	F (45.4%)<br>M (54.6%)	Cohort	Probability	National	0.632<br>(Medium)	•	•
Greece	SHARE [10–13,19,20,24,25]	2004–06<br>2006–10<br>2015	50–80+	5425	F (52.7%)<br>M (47.3%)	Cohort	Probability	National	0.887<br>(Very high)	•	•
Greenland	Bjerregaard et al. [66]	2017–19	20–75+	1419	F (55.6%)<br>M (44.4%)	Cross-sectional	Probability	National	0.786<br>(High)	•	•
Hong Kong	Yu et al. [67]	2015	18–80+	4683	F (85.2%)<br>M (14.8%)	Cross-sectional	Non-probability	Non-national	0.952<br>(Very high)	•	
Hungary	SHARE [10,14–16]	2010–12	50–80+	2788	F (56.1%)<br>M (43.9%)	Cohort	Probability	National	0.846<br>(Very high)	•	•
India	LASI [68]	2017–18	30–95+	61,580	F (57.7%)<br>M (42.3%)	Cohort	Probability	National	0.633<br>(Medium)	•	•
	PURE [2]	2003–09	35–69	25,747	F (56.3%)<br>M (43.7%)	Cohort	Probability/<br>Non-probability	Non-national		•	•
	SAGE [46]	2007	20–85+	10,199	F (60.2%)<br>M (39.8%)	Cohort	Probability	National		•	•
	SANSCOG [69]	2018–20	45–89	4663	F (52.6%)<br>M (47.4%)	Cohort	Non-probability	Non-national		•	•
Iran (Islamic Republic of)	PURE [2]	2003–09	35–69	6011	F (52.2%)<br>M (47.8%)	Cohort	Probability/<br>Non-probability	Non-national	0.774<br>(High)	•	•
	Mohammadian et al. [70]	2012	20–75+	1008	F (47.8%)<br>M (52.2%)	Cross-sectional	Probability	National		•	•
	Rostamzadeh et al. [71]	2018–19	20–79	4282	F (49.4%)<br>M (50.6%)	Cross-sectional	Non-probability	Non-national		•	
Ireland	GenoFit [72]	2017–20	20–89	9403	F (57.1%)<br>M (42.9%)	Cross-sectional	Non-probability	National	0.945<br>(Very high)	•	•
	SHARE [10,19,20]	2006–10	50–80+	794	F (53.8%)<br>M (46.2%)	Cohort	Probability	National		•	•
	TILDA [73]	2009–11	50–79	4778	F (53.8%)<br>M (46.2%)	Cohort	Probability	National		•	•
Israel	Langer et al. [74]	2015–16	20–75+	618	F (46.0%)<br>M (54.0%)	Cross-sectional	Non-probability	National	0.919<br>(Very high)	•	
	SHARE [10–13,19–23,34,35]	2004–06<br>2006–10<br>2013<br>2017	50–80+	2896	F (53.3%)<br>M (46.7%)	Cohort	Probability	National		•	•

Italy	ECHA [55]	2002–04	40–100+	374	F (51.6%): M (48.4%)	Cross-sectional	Non-probability	Non-national	0.895 (Very high)	•	•
	Lookup 7+ [75]	2015–19	18–80+	11,314	F (56.3%) M (43.7%)	Cross-sectional	Non-probability	National		•	
	SHARE [10–16,19–25]	2004–06 2006–10 2010–12 2013 2015	50–80+	6603	F (52.7%) M (47.3%)	Cohort	Probability	National		•	•
Japan	MEXT SPFMA [76–96]	2000–19[b]	20–79	676,842	F (49.7%) M (50.3%)	Cross-sectional	Non-probability	National	0.925 (Very high)	•	•[d]
	NILS-LSA [97]	2010–12	50–91	1164	F (48.8%) M (51.2%)	Cohort	Probability	Non-national		•	•
	ROAD [98]	2008–09	40–90+	2468	F (66.5%) M (33.5%)	Cohort	Probability	Non-national		•	•
	SONIC [99]	2010–12 2015 2018	70–72 80–82 90–92	1779	F (52.6%) M (47.4%)	Cohort	Non-probability	Non-national		•	•
	Taniguchi et al. [100]	2002–11	70–89	501	F (58.3%) M (41.7%)	Cohort	Non-probability	Non-national		•	•
	TMIG-LISA (HaCS, ICS, KLS, NCS, YLS) [101]	2002 2004 2008 2010 2011	65–85+	4551	F (53.9%) M (46.1%)	Cohort	Probability/ Non-probability	Non-national		•	•
Kazakhstan	PURE [2]	2003–09	35–69	2277	F (80.1%) M (19.9%)	Cohort	Probability/ Non-probability	Non-national	0.811 (Very high)	•	•
Kyrgyzstan	PURE [2]	2003–09	35–69	1993	F (73.7%) M (26.3%)	Cohort	Probability/ Non-probability	Non-national	0.692 (Medium)	•	•
Latvia	SHARE [10,34,35]	2017	50–80+	1545	F (62.1%) M (37.9%)	Cohort	Probability	National	0.863 (Very high)	•	•
Lithuania	HAPIEE [50]	2002–05	45–74	6854	F (54.3%) M (45.7%)	Cohort	Probability	Non-national	0.875 (Very high)	•	•
	SHARE [10,34,35]	2017	50–80+	1892	F (63.4%) M (36.6%)	Cohort	Probability	National		•	•
Luxembourg	SHARE [10,21–25]	2013 2015	50–80+	1819	F (52.0%) M (48.0%)	Cohort	Probability	National	0.930 (Very high)	•	•

Macao	PFSMR [102–106]	2001<br>2005<br>2010<br>2015<br>2020	20–79	21,043	F (56.7%)<br>M (43.3%)	Cross-sectional	Probability	National	0.946<br>(Very high)	•	•[c]
Malaysia	Lam et al. [107]	2013	60–75+	362	F (59.9%)<br>M (40.1%)	Cross-sectional	Non-probability	Non-national	0.803<br>(Very high)	•	
	MELoR [108]	2014–16	55–80+	1371	F (57.0%)<br>M (43.0%)	Cohort	Probability	Non-national		•	•
	PURE [2]	2003–09	35–69	10,538	F (57.2%)<br>M (42.8%)	Cohort	Probability/<br>Non-probability	Non-national		•	•
	Shah et al. [109]	2018–20	60–80+	1204	F (42.6%)<br>M (57.4%)	Cross-sectional	Probability	Non-national		•	•
Malta	SHARE [10,34,35]	2017	50–80+	1207	F (55.6%)<br>M (44.4%)	Cohort	Probability	National	0.918<br>(Very high)	•	•
Mexico	Enríquez-Reyna et al. [110]	2015	60–90	417	F (100%)	Cross-sectional	Probability	Non-national	0.758<br>(High)	•	•
	Malina et al. [111]	2000	20–60+	400	F (61.8%)<br>M (38.3%)	Cross-sectional	Non-probability	Non-national		•	•
	Núñez-Othón et al. [112]	2019	60–84	509	F (69.2%)<br>M (30.8%)	Cross-sectional	Non-probability	Non-national		•	•
	Rodríguez-García et al. [113,114]	2012–14	20–75+	1221	F (61.9%)<br>M (38.1%)	Cross-sectional	Non-probability	Non-national		•	•
	SABE [17]	1999–2000	50–85+	1596	F (73.6%)<br>M (26.4%)	Cross-sectional	Probability	Non-national		•	•
	SAGE [46]	2009–10	20–85+	2231	F (61.1%)<br>M (38.9%)	Cohort	Probability	National		•	•
Nepal	Bimali et al. [115]	2018	20–69	361	F (46.5%)<br>M (53.5%)	Cross-sectional	Non-probability	Non-national	0.602<br>(Medium)	•	
	Kim et al. [116]	2019	60–80+	1009	F (55.5%)<br>M (44.5%)	Cross-sectional	Non-probability	Non-National		•	•[c]
Netherlands	SHARE [10–16,19–23]	2004–06<br>2006–10<br>2010–12<br>2013	50–80+	5662	F (52.8%)<br>M (47.2%)	Cohort	Probability	National	0.941<br>(Very high)	•	•
Norway	FYSIOPRIM [117]	2011–12	20–75+	370	F (51.9%)<br>M (48.1%)	Cross-sectional	Non-probability	Non-national	0.961<br>(Very high)	•	•

	Kjær et al. [118]	2009	20–64	722	F (48.1%)<br>M (51.9%)	Cross-sectional	Probability	National		•	•
	Nilsen et al. [119]	2008–09	20–80+	560	F (55.5%)<br>M (44.5%)	Cross-sectional	Non-probability	Non-national		•	
	Tromsø [120]	2015–16	40–84	7869	F (54.5%)<br>M (45.5%)	Cohort	Probability	Non-national		•	•
Pakistan	PURE [2]	2003–09	35–69	2144	F (52.4%)<br>M (47.6%)	Cohort	Probability/<br>Non-probability	Non-national	0.544<br>(Low)	•	•
Philippines	Afable et al. [121]	2018	60–84	3406	F (61.8%)<br>M (38.2%)	Cross-sectional	Probability	National	0.699<br>(Medium)	•	•
	PURE [2]	2003–09	35–69	4852	F (71.3%)<br>M (28.7%)	Cohort	Probability/<br>Non-probability	Non-national		•	•
Poland	HAPIEE [50]	2002–05	45–74	6387	F (51.6%)<br>M (48.4%)	Cohort	Probability	Non-national	0.876<br>(Very high)	•	•
	PURE [2]	2003–09	35–69	2020	F (62.9%)<br>M (37.1%)	Cohort	Probability/<br>Non-probability	Non-national		•	•
	SHARE<br>[10,19,20,24,25,34,35]	2006–10<br>2015<br>2017	50–80+	5424	F (53.7%)<br>M (46.3%)	Cohort	Probability	National		•	•
	Wiśniowska-Szurlej et al.<br>[122]	2019–20	65–85+	405	F (66.9%)<br>M (33.1%)	Cross-sectional	Non-probability	Non-national		•	•
Portugal	NUP65 [123]	2015–16	65–85+	1496	F (58.0%)<br>M (42.0%)	Cross-sectional	Probability	National	0.866<br>(Very high)	•	•[c]
	SHARE [10,14–16]	2010–12	50–80+	1754	F (55.8%)<br>M (44.2%)	Cohort	Probability	National		•	•
Republic of<br>Korea	KNHANES [124,125]	2014–15<br>2016–17<br>2018–19	20–79	31,181	F (55.4%)<br>M (44.6%)	Cross-sectional	Probability	National	0.925<br>(Very high)	•	•
	MCST NSPF [126–130]	2009<br>2011<br>2013<br>2015<br>2017	20–84	21,641	F (48.6%)<br>M (51.4%)	Cross-sectional	Probability	National		•	•
Romania	SHARE [10,34,35]	2017	50–80+	1951	F (56.3%)<br>M (43.7%)	Cohort	Probability	National	0.821<br>(Very high)	•	•
Russian<br>Federation	KYH [131]	2015–18	40–69	3833	F (57.7%)<br>M (42.3%)	Cross-sectional	Probability	Non-national	0.822<br>(Very high)	•	•

	PURE [2]	2003–09	35–69	2991	F (72.4%) M (27.6%)	Cohort	Probability/ Non-probability	Non-national		•	•
	SAGE [46]	2007–10	20–85+	3337	F (62.9%) M (37.1%)	Cohort	Probability	National		•	•
	SAHR [132]	2006–09	55–89	1780	F (53.7%) M (46.3%)	Cohort	Probability	Non-national		•	•
	Turusheva et al. [133]	2009	65–85+	598	F (72.6%) M (27.4%)	Cohort	Probability	Non-national		•	•
Saudi Arabia	Alqahtani et al. [134]	2017–18	65–80	1048	F (51.2%) M (48.8%)	Cross-sectional	Non-probability	Non-national	0.875 (Very high)	•	•
	Alrashdan et al. [135]	2021	20–70	289	F (49.5%) M (50.5%)	Cross-sectional	Non-probability	Non-national		•	•
	PURE [2]	2003–09	35–69	2045	F (43.1%) M (56.9%)	Cohort	Probability/ Non-probability	Non-national		•	•
Singapore	SIHLS [136]	2009	60–89	2664	F (55.6%) M (44.4%)	Cross-sectional	Probability	National	0.939 (Very high)	•	•
	WiSE [137]	2012–13	60–85+	2043	F (53.5%) M (46.5%)	Cross-sectional	Probability	National		•	•
	Yishun [138]	2017–19	20–80+	534	F (57.7%) M (42.3%)	Cross-sectional	Probability	Non-national		•	•
Slovakia	SHARE [10,34,35]	2017	50–80+	1712	F (51.4%) M (48.6%)	Cohort	Probability	National	0.848 (Very high)	•	•
Slovenia	SHARE [10,14–16,21–25]	2010–12<br>2013<br>2015	50–80+	4280	F (55.7%) M (44.3%)	Cohort	Probability	National	0.918 (Very high)	•	•
South Africa	PURE [2]	2003–09	35–69	3236	F (68.3%) M (31.7%)	Cohort	Probability/ Non-probability	Non-national	0.713 (High)	•	•
	SAGE [46]	2007–08	20–85+	3260	F (58.1%) M (41.9%)	Cohort	Probability	National		•	•
Spain	Pizzara [139]	2009–11	20–65+	808	F (55.3%) M (44.7%)	Cross-sectional	Probability	Non-national	0.905 (Very high)	•	•
	SHARE [10–16,19–23]	2004–06<br>2006–10<br>2010–12<br>2013	50–80+	6767	F (53.3%) M (46.7%)	Cohort	Probability	National		•	•
State of Palestine	PURE [2]	2003–09	35–69	1561	F (50.0%) M (50.0%)	Cohort	Probability/ Non-probability	Non-national	0.715 (High)	•	•

Sweden	Axelsson et al. [140]	2013–16	20–70+	459	F (48.6%)<br>M (51.4%)	Cross-sectional	Non-probability	Non-national	0.947<br>(Very high)	•	•
	MrOS [141]	2001–04	70–80	999	M (100%)	Cohort	Probability	National		•	•[c]
	PURE [2]	2003–09	35–69	4097	F (52.8%)<br>M (47.2%)	Cohort	Probability/<br>Non-probability	Non-national		•	•
	SHARE [10–13,19–23]	2004–06<br>2006–10<br>2013	50–80+	5770	F (52.0%)<br>M (48.0%)	Cohort	Probability	National		•	•
Switzerland	COmPLETE [142]	2018–19	20–80+	607	F (49.1%)<br>M (50.9%)	Cross-sectional	Probability	Non-national	0.962<br>(Very high)	•	•
	SHARE [10–16,19,20]	2004–06<br>2006–10<br>2010–12	50–80+	3981	F (52.8%)<br>M (47.2%)	Cohort	Probability	National		•	•
	Wearing et al. [143]	2016–17	75–99	243	F (64.2%)<br>M (35.8%)	Cross-sectional	Non-probability	Non-national		•	•
	Werle et al. [144]	2006–07	20–85+	914	F (49.3%)<br>M (50.7%)	Cross-sectional	Non-probability	Non-national		•	
Taiwan	ILAS [145]	2011–12	50–75+	531	F (46.9%)<br>M (53.1%)	Cohort	Probability	Non-national	0.926<br>(High)	•	•
Türkiye	PURE [2]	2003–09	35–69	4058	F (60.6%)<br>M (39.4%)	Cohort	Probability/<br>Non-probability	Non-national	0.838<br>(Very high)	•	•
United Arab Emirates	PURE [2]	2003–09	35–69	914	F (71.4%)<br>M (28.6%)	Cohort	Probability/<br>Non-probability	Non-national	0.911<br>(Very high)	•	•
United Kingdom	ELSA [146,147]	2004–05<br>2008–09<br>2012–13	50–85+	11,648	F (54.4%)<br>M (45.6%)	Cohort	Probability	National	0.929<br>(Very high)	•	•
	EPIC-Norfolk [148]	2004–11	50–92	8357	F (55.0%)<br>M (45.0%)	Cohort	Non-probability	Non-national		•	•
	HCS [149]	2000–04	60–73	2600	F (54.0%)<br>M (46.0%)	Cohort	Non-probability	Non-national		•	•
	Me, Human [150]	2019	20–55+	587	F (60.3%)<br>M (39.7%)	Cross-sectional	Non-probability	Non-national		•	
	Twenty-07 [151]	2007–08	35–37,<br>52–62,<br>74–78	2337	F (54.6%)<br>M (45.4%)	Cohort	Probability	Non-national		•	•
	UKHLS [152]	2010–12	20–85+	14,035	F (56.1%)<br>M (43.9%)	Cohort	Probability	National		•	•

	Wozny et al. [153]	2010	20–49	135	F (51.9%)<br>M (48.1%)	Cross-sectional	Non-probability	Non-national		•	
United Republic of Tanzania	PURE [2]	2003–09	35–69	1688	F (77.4%)<br>M (22.6%)	Cohort	Probability/<br>Non-probability	Non-national	0.549<br>(Low)	•	•
United States of America	ASPREE [4]	2010–14	65–85+	2358	F (66.3%)<br>M (33.7%)	Cohort	Non-probability	Non-national	0.921<br>(Very high)	•	•
	HRS [154,155]	2006<br>2008<br>2010<br>2012<br>2014<br>2016<br>2018	50–90+	23,946	F (56.7%)<br>M (43.3%)	Cohort	Probability	National		•	•
	MSK-FIT [156]	2017–21	20–79	2243	F (73.4%)<br>M (26.6%)	Cross-sectional	Non-probability	Non-national		•	•
	NHANES [157]	2011–14	20–79	9066	F (49.8%)<br>M (50.2%)	Cross-sectional	Probability	National		•	•
	NIH Toolbox [158,159]	2011	20–85	1394	F (64.1%)<br>M (35.9%)	Cross-sectional	Probability	National		•	•
Uruguay	SABE [17]	1999–2000	60–85+	177	F (61.6%)<br>M (38.4%)	Cross-sectional	Probability	Non-national	0.809<br>(Very high)	•	•
Zimbabwe	PURE [2]	2003–09	35–69	815	F (71.8%)<br>M (28.2%)	Cohort	Probability/<br>Non-probability	Non-national	0.593<br>(Medium)	•	•

Notes: HDI values of 0.800, 0.700 and 0.550 were used as thresholds for very high, high, and medium human development, respectively [160]; the sample sizes reflect the number of absolute HGS values; study design was coded as cross-sectional or cohort, despite only including cross-sectional data from cohort studies (unique individuals from baseline or refreshment samples); sampling strategy was coded as probability (i.e., random selection) or non-probability (i.e., non-random selection); sample base was coded as national (i.e., national sampling) or non-national (i.e., non-national sampling, including regional [state/provincial] and local [city/district] sampling); because the United Nations did not provide HDI estimates for Greenland, Macao, and Taiwan they were sourced from references 161, 162, and 163, respectively.

[a] China refers to the mainland of China;

[b] Data collected annually across the time period; data (e.g., sample sizes, year(s) of testing) may differ to published values because data measured from 2000 onwards were re-analyzed to meet our inclusion criteria;

[c] Mean normalized handgrip strength was estimated by dividing mean absolute handgrip strength (kg) by mean height in meters (m) squared, and the standard deviation of normalized handgrip strength was estimated from sex-specific sample-weighted coefficients of variation;

[d] Mean and standard deviation normalized handgrip strength were estimated for only the 2000–2005 testing years.

Abbreviations: HDI = Human Development Index (2021 estimates) [160]; HGS = handgrip strength; F = Female; M = Male; SAR = Special Administrative Region. Studies: ASPREE = ASPirin in Reducing Events in the Elderly study; CHARLS = China Health and Retirement Longitudinal Study; CHMS = Canadian Health Measures Survey; CLSA = Canadian Longitudinal Study on Ageing; CNSAF = Chinese National Survey on Adults' Fitness; COmPLETE = CardiO-PuLmonary Exercise Testing health study; DBCS = Danish Birth Cohort Studies of 1905, 1910, 1911,

and 1915; DTR (LSADT, MADT, MIDT) = Danish Twin Registry (Longitudinal Study of Aging Danish Twins, Middle Age Danish Twins, MIddle age Danish Twins); ECHA = European Challenge for Healthy Aging study; EDOC = Estudo das Doenças Crônicas (English translation: Study of Chronic Diseases); ELSA = English Longitudinal Study of Ageing; ELSI-Brazil = Brazilian Longitudinal Study of Aging; ENSIN = Encuesta Nacional de la Situacion Nutricional en Colombia (English translation: 2015 National Nutritional Survey); EPIC-Norfolk = European Prospective Investigation into Cancer-Norfolk study; Fibra-BR = Frailty in Brazilian Older People study; FORMoSA = Bavarian Research Foundation-Sarcopenia and Osteoporosis study; FYSIOPRIM = Research Program for Physiotherapy in Primary Health Care study; GenoFit = GenoFit study; GOS = Geelong Osteoporosis Study; HAPIEE = Health, Alcohol and Psychosocial factors In Eastern Europe study; HCS = Hertfordshire Cohort Study; Health2006 = Health2006 study; HRS = Health and Retirement Study; ILAS = I-Lan Longitudinal Aging Study; KNHANES = Korea National Health and Nutrition Examination Survey; KORA-Age = Kooperative Gesundheitsforschung in der Region Augsburg (English translation: Cooperative Health Research in the Region of Augsburg study); KYH = Know Your Heart study; LASI = Longitudinal Ageing Study in India; Lookup 7+ = Longevity check-up 7+ study; MCST NSPF = Ministry of Culture, Sports and Tourism’s National Survey of Physical Fitness; Me, Human = Me, Human project; MELoR = Malaysian Elders Longitudinal Research study; MEXT SPFMA = Ministry of Education, Culture, Sports, Science and Technology’s Survey on Physical Fitness and Motor Abilities; MrOS = Osteoporotic Fractures in Men (Sweden cohort) study; MSK-FIT = Musculoskeletal, Function, Imaging, and Tissue Resource Core; NAKO = NAKO Gesundheitsstudie (English translation: German National Cohort); NHANES = National Health and Nutrition Examination Survey; NIH Toolbox = U.S. National Institutes of Health Toolbox study; NILS-LSA = National Institute for Longevity Sciences-Longitudinal Study of Aging; NUP65 = Nutrition UP 65 study; NWAHS = North West Adelaide Health Study; OA = Outdoor Active study; Pizzara = Pizzara study; PFSMR = Physical Fitness Study of Macao SAR Residents; PLSAW = Perth Longitudinal Study of Ageing Women; PNAFS = Nutrition, Physical Activity and Health Survey; PURE = Prospective Urban Rural Epidemiology study; ROAD = Research on Osteoarthritis/Osteoporosis Against Disability study; SABE = SAlud, Bienestar, y Envejecimiento (English translation: Survey on Health, Well-Being, and Aging in Latin America and the Caribbean); SAGE = World Health Organization Study on global AGEing and adult health; SAHR = Study of Stress, Aging, and Health in Russia; SANSCOG = Srinivaspura NeuoSenescence, and COGnition study; Saúde-AC = English translation: Health-Antônio Carlos (Santa Catarina) study; SHARE = Survey of Health, Ageing and Retirement in Europe; SIHLS = Social Isolation, Health, and Lifestyles Survey; SONIC = Septuagenarians, Octogenarians, Nonagenarians Investigation with Centenarians study; SOEP = German Socio-Economic Panel survey; TILDA = The Irish Longitudinal Study on Ageing; TMIG-LISA (HaCS, ICS, KLS, NCS, YLS) = Tokyo Metropolitan Institute of Gerontology-Longitudinal Interdisciplinary Study on Aging (Hatoyama Cohort Study, Itabashi Cohort Studies of 2002 and 2011, Kusatsu Longitudinal Study, Nangai Cohort Study, Yoita Longitudinal Study); Tromsø = Norwegian Tromsø study; Twenty-07 = West of Scotland Twenty-07 study; UKHLS = Understanding Society: The UK Household Longitudinal Study; WiSE = Well-being of Singapore Elderly study; Yishun = Yishun study.

Supplementary Table 4B. Handgrip strength test protocols for the included studies by country or region.

Country or region	Study	Dyna-mometer	Body position	Shoulder position	Elbow position	Radioulnar position	Wrist position	Handle position	Time (min) between reps	Verbal support	Testing hand	Reps per hand	Summary statistic
Argentina	PURE [2]	Hydraulic	Standing	Adducted	Flexed	Neutral	Neutral	Hand size	<1	Yes	Both	3	Maximum
Australia	1000 Norms Project [3]	Electronic	Seated	Adducted	Flexed	Neutral	Neutral	Standard	<1	Yes	Dominant	3	Maximum
	ASPREE [4]	Hydraulic	Seated	Adducted	Flexed	Neutral	Neutral	Hand size	<1	Yes	Both	3	Maximum
	GOS [5,6]	Hydraulic	Seated	Adducted	Flexed	Neutral	Neutral	Standard	<1	No	Both	2	Maximum
		Electronic	Seated	Adducted	Flexed	Neutral	Neutral	Standard	<1	Yes	Both	3	Maximum
	Ingram et al. [7]	Electronic	Seated	Adducted	Flexed	Neutral	Neutral	Standard	<1	Yes	Dominant	3	Maximum
	NWAHS [8]	Hydraulic	Seated	Adducted	Flexed	Neutral	Neutral	Standard	NS	Yes	Both	3	Maximum
	PLSAW [9]	Hydraulic	Standing	Adducted	Extended	Neutral	Neutral	Hand size	NS	Yes	Dominant	3	Maximum
Austria	SHARE [10–16]	Mechanical	Standing/ Seated[b]	Adducted	Flexed	Neutral	Neutral	Hand size	NS	Yes	Both	2	Maximum
Bangladesh	PURE [2]	Hydraulic	Standing	Adducted	Flexed	Neutral	Neutral	Hand size	<1	Yes	Both	3	Maximum
Barbados	SABE [17,18]	Mechanical	Seated	Adducted	Flexed	Supinated	Neutral	Hand size	≥1	Yes	Dominant	2	Maximum
Belgium	SHARE [10–16,19–25]	Mechanical	Standing/ Seated[b]	Adducted	Flexed	Neutral	Neutral	Hand size	NS	Yes	Both	2	Maximum
Brazil	Budziareck et al. [26]	Hydraulic	Seated	Adducted	Flexed	Neutral	Neutral	Hand size	≥1	Yes	Both	3	Average
	EDOC [27]	Hydraulic	Seated	Adducted	Flexed	Neutral	Neutral	Standard	≥1	Yes	Both	3	Maximum
	ELSI-Brazil [28]	Mechanical	Seated	Adducted	Flexed	Neutral	Neutral	Hand size	≥1	Yes	Dominant	3	Maximum
	Fernandes et al. [29]	Hydraulic	Seated	Adducted	Flexed	Neutral	Neutral	Standard	≥1	No	Both	3	Maximum
	Fibra-BR [30]	Hydraulic	Seated	Adducted	Flexed	Neutral	Neutral	Hand size	≥1	Yes	Dominant	2	Maximum
	PNAFS [31]	Mechanical	Standing	Adducted	Extended	Neutral	Neutral	Hand size	≥1	NS	Both	3	Maximum
	PURE [2]	Hydraulic	Standing	Adducted	Flexed	Neutral	Neutral	Hand size	<1	Yes	Both	3	Maximum
	SABE [17,32]	Mechanical	Seated	Adducted	Flexed	Supinated	Neutral	Hand size	≥1	Yes	Dominant	2	Maximum
	Saúde-AC [33]	Mechanical	Seated	Adducted	Flexed	Supinated	Neutral	Hand size	≥1	Yes	Dominant	2	Maximum
Bulgaria	SHARE [10,34,35]	Mechanical	Standing/	Adducted	Flexed	Neutral	Neutral	Hand size	NS	Yes	Both	2	Maximum

			Seated[b]										
Canada	CHMS [36–39]	Mechanical	Standing	Abducted[c]	Extended	Neutral	Neutral	Hand size	NS	NS	Both	2	Maximum
	CLSA [40]	Electronic	Seated	Adducted	Flexed	Neutral	Neutral	Standard	<1	Yes	Dominant	3	Maximum
	PURE [2]	Hydraulic	Standing	Adducted	Flexed	Neutral	Neutral	Hand size	<1	Yes	Both	3	Maximum
Chile	Gómez-Campos et al. [41]	Hydraulic	Seated	Adducted	Flexed	Neutral	Neutral	Hand size	≥1	NS	Both[d]	2	Maximum
	Leal Cárcamo et al. [42]	Hydraulic	Seated	Adducted	Flexed	Neutral	Neutral	Standard	≥1	Yes	Both	3	Average
	PURE [2]	Hydraulic	Standing	Adducted	Flexed	Neutral	Neutral	Hand size	<1	Yes	Both	3	Maximum
	SABE [17]	Mechanical	Seated	Adducted	Flexed	Supinated	Neutral	Hand size	≥1	Yes	Dominant	2	Maximum
China[a]	CHARLS [43,44]	Mechanical	Standing	Adducted	Flexed	Neutral	Neutral	Hand size	<1	Yes	Both	2	Maximum
	CNSAF [45]	Electronic	Standing	Adducted	Extended	Neutral	Neutral	Hand size	NS	Yes	Dominant	2	Maximum
	PURE [2]	Hydraulic	Standing	Adducted	Flexed	Neutral	Neutral	Hand size	<1	Yes	Both	3	Maximum
	SAGE [46]	Mechanical	Seated	Adducted	Flexed	Neutral	Neutral	Hand size	<1	NS	Both	2	Maximum
	Shanghai Administration of Sports [47]	Electronic	Standing	Adducted	Extended	Neutral	Neutral	Hand size	NS	Yes	Dominant	2	Maximum
Colombia	ENSIN [48]	Electronic	Standing	Adducted	Extended	Neutral	Neutral	Hand size	<1	Yes	Both	2–3[e]	Maximum
	PURE [2]	Hydraulic	Standing	Adducted	Flexed	Neutral	Neutral	Hand size	<1	Yes	Both	3	Maximum
	SABE [49]	Electronic	Standing	Adducted	Extended	Neutral	Neutral	Hand size	<1	Yes	Both	2–3[e]	Maximum
Croatia	SHARE [10,24,25,34,35]	Mechanical	Standing/ Seated[b]	Adducted	Flexed	Neutral	Neutral	Hand size	NS	Yes	Both	2	Maximum
Cuba	SABE [17,18]	Mechanical	Seated	Adducted	Flexed	Supinated	Neutral	Hand size	≥1	Yes	Dominant	2	Maximum
Cyprus	SHARE [10,34,35]	Mechanical	Standing/ Seated[b]	Adducted	Flexed	Neutral	Neutral	Hand size	NS	Yes	Both	2	Maximum
Czechia	HAPIEE [50]	Mechanical	Standing	Adducted	Flexed	Neutral	Neutral	Hand size	≥1	Yes	Both	2	Maximum
	SHARE [10,14–16,19–23]	Mechanical	Standing/ Seated[b]	Adducted	Flexed	Neutral	Neutral	Hand size	NS	Yes	Both	2	Maximum
Denmark	DBCS (1905, 1910, 1911, 1915) [51–53]	Mechanical	Standing/ Seated[f]	Adducted	Flexed	Neutral	Neutral	Hand size	NS	Yes	Both/ Dominant[g]	2–3[h]	Maximum

	DTR (LSADT, MADT, MIDT) [51,54]	Mechanical	Standing/ Seated[f]	Adducted	Flexed	Neutral	Neutral	Hand size	NS	Yes	Both	3	Maximum
	ECHA [55]	Mechanical	Seated	Adducted	Flexed	Neutral	Neutral	Hand size	NS	Yes	Dominant	3	Maximum
	Health2006 [56]	Hydraulic	Seated	Adducted	Flexed	Neutral	Neutral	Hand size	<1	Yes	Dominant	3	Maximum
	SHARE [10–16,19–25]	Mechanical	Standing/ Seated[b]	Adducted	Flexed	Neutral	Neutral	Hand size	NS	Yes	Both	2	Maximum
	Suetta et al. [57]	Electronic	Seated	Adducted	Flexed	Neutral	Neutral	Hand size	<1	Yes	Dominant	3	Maximum
Ecuador	PURE [2]	Hydraulic	Standing	Adducted	Flexed	Neutral	Neutral	Hand size	<1	Yes	Both	3	Maximum
Estonia	SHARE [10,14–16,24,25]	Mechanical	Standing/ Seated[b]	Adducted	Flexed	Neutral	Neutral	Hand size	NS	Yes	Both	2	Maximum
Finland	SHARE [10,34,35]	Mechanical	Standing/ Seated[b]	Adducted	Flexed	Neutral	Neutral	Hand size	NS	Yes	Both	2	Maximum
France	ECHA [55]	Mechanical	Seated	Adducted	Flexed	Neutral	Neutral	Hand size	NS	Yes	Dominant	3	Maximum
	Hogrel [58]	Hydraulic	Seated	Adducted	Extended	Neutral	Neutral	Hand size	<1	Yes	Both	2+[e]	Maximum
	SHARE [10–16,19,20,24,25]	Mechanical	Standing/ Seated[b]	Adducted	Flexed	Neutral	Neutral	Hand size	NS	Yes	Both	2	Maximum
Germany	FORMoSA [59]	Hydraulic	Standing	Adducted	Extended	Neutral	Neutral	Hand size	<1	No	Both	2	Maximum
	KORA-Age [60]	Hydraulic	Standing	Adducted	Flexed	Neutral	Neutral	Hand size	<1	NS	Dominant	3	Maximum
	NAKO [60]	Electronic	Seated	Adducted	Flexed	Neutral	Neutral	Standard	<1	No	Both	3	Maximum
	OA [61]	Electronic	Standing	Adducted	Flexed	Neutral	Neutral	Hand size	<1	Yes	Both	2	Maximum
	Schilling et al. [62]	Electronic	Seated	Adducted	Flexed	Neutral	Neutral	Standard	<1	Yes	Both	2	Maximum
	SHARE [10–13,19–23]	Mechanical	Standing/ Seated[b]	Adducted	Flexed	Neutral	Neutral	Hand size	NS	Yes	Both	2	Maximum
	SOEP [63–65]	Mechanical	Standing	Adducted	Flexed	Neutral	Neutral	Hand size	NS	NS	Both	2	Maximum
Ghana	SAGE [46]	Mechanical	Seated	Adducted	Flexed	Neutral	Neutral	Hand size	<1	NS	Both	2	Maximum
Greece	SHARE [10–13,19,20,24,25]	Mechanical	Standing/ Seated[b]	Adducted	Flexed	Neutral	Neutral	Hand size	NS	Yes	Both	2	Maximum
Greenland	Bjerregaard et al. [66]	Electronic	Seated	Adducted	Flexed	Neutral	Neutral	Hand size	<1	No	Both	3	Maximum
Hong Kong	Yu et al. [67]	Electronic	Standing	Adducted	Extended	Neutral	Neutral	Standard	≥1	Yes	Both	2	Average
Hungary	SHARE [10,14–16]	Mechanical	Standing/ Seated[b]	Adducted	Flexed	Neutral	Neutral	Hand size	NS	Yes	Both	2	Maximum

India	LASI [68]	Mechanical	Standing/ Seated[f]	Adducted	Flexed	Neutral	Neutral	Hand size	≥1	NS	Both	2	Maximum
	PURE [2]	Hydraulic	Standing	Adducted	Flexed	Neutral	Neutral	Hand size	<1	Yes	Both	3	Maximum
	SAGE [46]	Mechanical	Seated	Adducted	Flexed	Neutral	Neutral	Hand size	<1	NS	Both	2	Maximum
	SANSCOG [69]	Electronic	Seated	Adducted	Flexed	Neutral	Neutral	Hand size	<1	Yes	Both	2	Maximum
Iran (Islamic Republic of)	PURE [2]	Hydraulic	Standing	Adducted	Flexed	Neutral	Neutral	Hand size	<1	Yes	Both	3	Maximum
	Mohammadian et al. [70]	Hydraulic	Seated	Adducted	Flexed	Neutral	Neutral	Standard	≥1	Yes	Both	3	Maximum
	Rostamzadeh et al. [71]	Hydraulic	Seated	Adducted	Flexed	Neutral	Neutral	Standard	≥1	Yes	Both	3	Maximum
Ireland	GenoFit [72]	Hydraulic	Standing	Adducted	Extended	Neutral	Neutral	Hand size	≥1	Yes	Both	2	Maximum
	SHARE [10,19,20]	Mechanical	Standing/ Seated[b]	Adducted	Flexed	Neutral	Neutral	Hand size	NS	Yes	Both	2	Maximum
	TILDA [73]	Hydraulic	Standing/ Seated[b]	Adducted	Flexed	Neutral	Neutral	Hand size	<1	No	Both	2	Maximum
Israel	Langer et al. [74]	Hydraulic	Seated	Adducted	Flexed	Neutral	Neutral	Standard	≥1	No	Both	3	Maximum
	SHARE [10–13,19–23,34,35]	Mechanical	Standing/ Seated[b]	Adducted	Flexed	Neutral	Neutral	Hand size	NS	Yes	Both	2	Maximum
Italy	ECHA [55]	Mechanical	Seated	Adducted	Flexed	Neutral	Neutral	Hand size	NS	Yes	Dominant	3	Maximum
	Lookup 7+ [75]	Hydraulic	Seated	Adducted	Flexed	Neutral	Neutral	Hand size	NS	Yes	Both	1	Maximum
	SHARE [10–16,19–25]	Mechanical	Standing/ Seated[b]	Adducted	Flexed	Neutral	Neutral	Hand size	NS	Yes	Both	2	Maximum
Japan	MEXT SPFMA [76–96]	Mechanical	Standing	Adducted	Extended	Neutral	Neutral	Hand size	NS	NS	Both	2	Maximum/ Average of maxima[i]
	NILS-LSA [97]	Electronic	Standing	Adducted	Extended	Neutral	Neutral	Hand size	NS	No	Both	2	Maximum
	ROAD [98]	Mechanical	Standing	Abducted[c]	Extended	Neutral	Neutral	Hand size	NS	No	Both	1	Maximum
	SONIC [99]	Mechanical	Seated	Adducted	Flexed	Neutral	Neutral	Hand size	<1	Yes	Dominant	2	Maximum
	Taniguchi et al. [100]	Hydraulic	Standing/ Seated[b]	Adducted	Extended	Neutral	Neutral	Hand size	<1	Yes	Dominant	2	Maximum
	TMIG-LISA (HaCS, ICS, KLS, NCS, YLS) [101]	Mechanical	Standing	Adducted	Extended	Neutral	Neutral	Hand size	<1	Yes	Dominant	1	Maximum

Kazakhstan	PURE [2]	Hydraulic	Standing	Adducted	Flexed	Neutral	Neutral	Hand size	<1	Yes	Both	3	Maximum
Kyrgyzstan	PURE [2]	Hydraulic	Standing	Adducted	Flexed	Neutral	Neutral	Hand size	<1	Yes	Both	3	Maximum
Latvia	SHARE [10,34,35]	Mechanical	Standing/ Seated[b]	Adducted	Flexed	Neutral	Neutral	Hand size	NS	Yes	Both	2	Maximum
Lithuania	HAPIEE [50]	Mechanical	Standing	Adducted	Flexed	Neutral	Neutral	Hand size	≥1	Yes	Both	2	Maximum
	SHARE [10,34,35]	Mechanical	Standing/ Seated[b]	Adducted	Flexed	Neutral	Neutral	Hand size	NS	Yes	Both	2	Maximum
Luxembourg	SHARE [10,21–25]	Mechanical	Standing/ Seated[b]	Adducted	Flexed	Neutral	Neutral	Hand size	NS	Yes	Both	2	Maximum
Macao	PFSMR [102]	Electronic	Standing	Adducted	Extended	Neutral	Neutral	Hand size	NS	NS	Dominant	3	Maximum
	PFSMR [103–106]	Electronic	Standing	Adducted	Extended	Neutral	Neutral	Hand size	NS	NS	Dominant	2	Maximum
Malaysia	Lam et al. [107]	Hydraulic	Seated	Adducted	Flexed	Neutral	Neutral	Hand size	<1	Yes	Both[d]	3	Average
	MELoR [108]	Electronic	Seated	Adducted	Flexed	Neutral	Neutral	Hand size	≥1	Yes	Both	3	Maximum
	PURE [2]	Hydraulic	Standing	Adducted	Flexed	Neutral	Neutral	Hand size	<1	Yes	Both	3	Maximum
	Shah et al. [109]	Mechanical	Standing	Adducted	Extended	Neutral	Neutral	Hand size	<1	Yes	Dominant	2	Average
Malta	SHARE [10,34,35]	Mechanical	Standing/ Seated[b]	Adducted	Flexed	Neutral	Neutral	Hand size	NS	Yes	Both	2	Maximum
Mexico	Enríquez-Reyna et al. [110]	Electronic	Standing	Abducted[c]	Extended	Neutral	Neutral	Hand size	≥1	Yes	Both	2	Maximum
	Malina et al. [111]	Mechanical	Standing	Adducted	Extended	Neutral	Neutral	Hand size	<1	No	Both	3	Maximum
	Núñez-Othón et al. [112]	Hydraulic	Standing	Adducted	Extended	Neutral	Neutral	Hand size	<1	Yes	Both	2	Maximum
	Rodríguez-García et al. [113,114]	Mechanical	Standing	Adducted	Extended	Neutral	Neutral	Hand size	≥1	No	Both	3	Maximum
	SABE [17]	Mechanical	Seated	Adducted	Flexed	Supinated	Neutral	Hand size	≥1	Yes	Dominant	2	Maximum
	SAGE [46]	Mechanical	Seated	Adducted	Flexed	Neutral	Neutral	Hand size	<1	NS	Both	2	Maximum
Nepal	Bimali et al. [115]	Hydraulic	Seated	Adducted	Flexed	Neutral	Neutral	Standard	≥1	Yes	Both	3	Maximum
	Kim et al. [116]	Electronic	Standing	Adducted	Extended	Neutral	Neutral	Standard	≥1	Yes	Both[d]	2	Average
Netherlands	SHARE [10–16,19–23]	Mechanical	Standing/ Seated[b]	Adducted	Flexed	Neutral	Neutral	Hand size	NS	Yes	Both	2	Maximum
Norway	FYSIOPRIM [117]	Hydraulic	Seated	Adducted	Flexed	Neutral	Neutral	Standard	<1	Yes	Both	2	Maximum

	Kjær et al. [118]	Hydraulic	Standing	Adducted	Extended	Neutral	Neutral	Hand size	NS	No	Dominant	3	Maximum
	Nilsen et al. [119]	Electronic	Seated	Adducted	Flexed	Neutral	Neutral	Standard	NS	NS	Both[d]	1	Maximum
	Tromsø [120]	Electronic	Seated	Adducted	Flexed	Neutral	Neutral	Standard	<1	Yes	Both	3	Maximum
Pakistan	PURE [2]	Hydraulic	Standing	Adducted	Flexed	Neutral	Neutral	Hand size	<1	Yes	Both	3	Maximum
Philippines	Afable et al. [121]	Mechanical	Standing	Adducted	Extended	Neutral	Neutral	Hand size	NS	No	Both	3	Maximum
	PURE [2]	Hydraulic	Standing	Adducted	Flexed	Neutral	Neutral	Hand size	<1	Yes	Both	3	Maximum
Poland	HAPIEE [50]	Mechanical	Standing	Adducted	Flexed	Neutral	Neutral	Hand size	≥1	Yes	Both	2	Maximum
	PURE [2]	Hydraulic	Standing	Adducted	Flexed	Neutral	Neutral	Hand size	<1	Yes	Both	3	Maximum
	SHARE [10,19,20,24,25,34,35]	Mechanical	Standing/ Seated[b]	Adducted	Flexed	Neutral	Neutral	Hand size	NS	Yes	Both	2	Maximum
	Wiśniowska-Szurlej et al. [122]	Electronic	Seated	Adducted	Flexed	Neutral	Neutral	Standard	≥1	NS	Dominant	3	Average
Portugal	NUP65 [123]	Electronic	Seated	Adducted	Flexed	Neutral	Neutral	Standard	≥1	NS	Non-dominant	3	Maximum
	SHARE [10,14–16]	Mechanical	Standing/ Seated[b]	Adducted	Flexed	Neutral	Neutral	Hand size	NS	Yes	Both	2	Maximum
Republic of Korea	KNHANES [124,125]	Electronic	Standing	Adducted	Extended	Neutral	Neutral	Hand size	≥1	Yes	Both	3	Maximum
	MCST NSPF [126–130]	Mechanical	Standing	Adducted	Extended	Neutral	Neutral	Hand size	≥1	Yes	Both	2	Maximum
Romania	SHARE [10,34,35]	Mechanical	Standing/ Seated[b]	Adducted	Flexed	Neutral	Neutral	Hand size	NS	Yes	Both	2	Maximum
Russian Federation	KYH [131]	Electronic	Seated	Adducted	Flexed	Neutral	Neutral	Standard	<1	Yes	Both	3	Maximum
	PURE [2]	Hydraulic	Standing	Adducted	Flexed	Neutral	Neutral	Hand size	<1	Yes	Both	3	Maximum
	SAGE [46]	Mechanical	Seated	Adducted	Flexed	Neutral	Neutral	Hand size	<1	NS	Both	2	Maximum
	SAHR [132]	Mechanical	Standing/ Seated[b]	Adducted	Flexed	Neutral	Neutral	Hand size	NS	Yes	Both	3	Maximum
	Turusheva et al. [133]	Electronic	Seated	Adducted	Flexed	Neutral	Neutral	Hand size	<1	Yes	Both	3	Maximum
Saudi Arabia	Alqahtani et al. [134]	Electronic	Standing	Adducted	Extended	Neutral	Neutral	Standard	≥1	Yes	Both	3	Maximum
	Alrashdan et al. [135]	Hydraulic	Seated	Adducted	Flexed	Neutral	Neutral	Standard	≥1	NS	Both	3	Maximum
	PURE [2]	Hydraulic	Standing	Adducted	Flexed	Neutral	Neutral	Hand size	<1	Yes	Both	3	Maximum
Singapore	SIHLS [136]	Mechanical	Standing	Adducted	Extended	Neutral	Neutral	Hand size	NS	No	Both	2	Maximum

	WiSE [137]	Electronic	Seated	Adducted	Flexed	Neutral	Neutral	Standard	<1	Yes	Dominant	2	Maximum
	Yishun [138]	Electronic	Seated	Adducted	Flexed	Neutral	Neutral	Hand size	≥1	Yes	Both	2	Maximum
Slovakia	SHARE [10,34,35]	Mechanical	Standing/ Seated[b]	Adducted	Flexed	Neutral	Neutral	Hand size	NS	Yes	Both	2	Maximum
Slovenia	SHARE [10,14–16,21–25]	Mechanical	Standing/ Seated[b]	Adducted	Flexed	Neutral	Neutral	Hand size	NS	Yes	Both	2	Maximum
South Africa	PURE [2]	Hydraulic	Standing	Adducted	Flexed	Neutral	Neutral	Hand size	<1	Yes	Both	3	Maximum
	SAGE [46]	Mechanical	Seated	Adducted	Flexed	Neutral	Neutral	Hand size	<1	NS	Both	2	Maximum
Spain	Pizzara [139]	Hydraulic	Seated	Adducted	Flexed	Neutral	Neutral	Hand size	≥1	Yes	Dominant	3	Maximum
	SHARE [10–16,19–23]	Mechanical	Standing/ Seated[b]	Adducted	Flexed	Neutral	Neutral	Hand size	NS	Yes	Both	2	Maximum
State of Palestine	PURE [2]	Hydraulic	Standing	Adducted	Flexed	Neutral	Neutral	Hand size	<1	Yes	Both	3	Maximum
Sweden	Axelsson et al. [140]	Hydraulic	Standing	Adducted	Flexed	Neutral	Neutral	Standard	NS	NS	Both	1	Maximum
	MrOS [141]	Hydraulic	Seated	Adducted	Flexed	Neutral	Neutral	Hand size	NS	NS	Both[d]	2	Maximum
	PURE [2]	Hydraulic	Standing	Adducted	Flexed	Neutral	Neutral	Hand size	<1	Yes	Both	3	Maximum
	SHARE [10–13,19–23]	Mechanical	Standing/ Seated[b]	Adducted	Flexed	Neutral	Neutral	Hand size	NS	Yes	Both	2	Maximum
Switzerland	COmPLETE [142]	Electronic	Standing	Adducted	Extended	Neutral	Neutral	Hand size	≥1	Yes	Dominant	3	Maximum
	SHARE [10–16,19,20]	Mechanical	Standing/ Seated[b]	Adducted	Flexed	Neutral	Neutral	Hand size	NS	Yes	Both	2	Maximum
	Wearing et al. [143]	Hydraulic	Seated	Adducted	Flexed	Neutral	Neutral	Standard	<1	No	Both	3	Maximum
	Werle et al. [144]	Hydraulic	Seated	Adducted	Flexed	Neutral	Neutral	Standard	<1	NS	Both[d]	3	Average
Taiwan	ILAS [145]	Mechanical	Standing	Adducted	Extended	Neutral	Neutral	Hand size	<1	Yes	Dominant	3	Maximum
Türkiye	PURE [2]	Hydraulic	Standing	Adducted	Flexed	Neutral	Neutral	Hand size	<1	Yes	Both	3	Maximum
United Arab Emirates	PURE [2]	Hydraulic	Standing	Adducted	Flexed	Neutral	Neutral	Hand size	<1	Yes	Both	3	Maximum
United Kingdom	ELSA [146,147]	Mechanical	Standing/ Seated[f]	Adducted	Flexed	Neutral	Neutral	Hand size	NS	Yes	Both	3	Maximum
	EPIC-Norfolk [148]	Mechanical	Standing/ Seated[b]	Adducted	Flexed	Neutral	Neutral	Hand size	NS	Yes	Both	2	Maximum

	HCS [149]	Hydraulic	Seated	Adducted	Flexed	Neutral	Neutral	Hand size	NS	Yes	Both	3	Maximum
	Me, Human [150]	Hydraulic	Seated	Adducted	Flexed	Neutral	Neutral	Hand size	≥1	Yes	Both	2	Maximum
	Twenty-07 [151]	Hydraulic	Standing	Adducted	Flexed	Neutral	Neutral	Hand size	NS	Yes	Both	3	Maximum
	UKHLS [152]	Mechanical	Standing/ Seated[f]	Adducted	Flexed	Neutral	Neutral	Hand size	NS	NS	Both	3	Maximum
	Wozny et al. [153]	Hydraulic	Seated	Adducted	Flexed	Neutral	Neutral	Standard	<1	Yes	Both[d]	3	Average
United Republic of Tanzania	PURE [2]	Hydraulic	Standing	Adducted	Flexed	Neutral	Neutral	Hand size	<1	Yes	Both	3	Maximum
United States of America	ASPREE [4]	Hydraulic	Seated	Adducted	Flexed	Neutral	Neutral	Hand size	<1	Yes	Both	3	Maximum
	HRS [154,155]	Mechanical	Standing/ Seated[b]	Adducted	Flexed	Neutral	Neutral	Hand size	NS	NS	Both	2	Maximum
	MSK-FIT [156]	Electronic	Seated	Adducted	Flexed	Neutral	Neutral	Standard	<1	Yes	Dominant	3	Maximum
	NHANES [157]	Electronic	Standing	Adducted	Extended	Neutral	Neutral	Hand size	≥1	Yes	Both	3	Maximum
	NIH Toolbox [158,159]	Hydraulic	Seated	Adducted	Flexed	Neutral	Neutral	Standard	NS	Yes	Both	1	Maximum
Uruguay	SABE [17]	Mechanical	Seated	Adducted	Flexed	Supinated	Neutral	Hand size	≥1	Yes	Dominant	2	Maximum
Zimbabwe	PURE [2]	Hydraulic	Standing	Adducted	Flexed	Neutral	Neutral	Hand size	<1	Yes	Both	3	Maximum

Notes: Test protocol data in this table may differ to published data (e.g., summary statistic) because we recalculated such data to harmonize for methodological variation by adjusting HGS values to a common metric, test, and reporting protocol. Types of handgrip dynamometers included hydraulic (i.e., used hydraulic fluid [e.g., Jamar J00105 and 5030J1, Seahan SH5001]), electronic (i.e., used electronic load cell [e.g., Jamar Plus+, Takei TKK 5401, Seahan DHD-3 (SH1003)]), and mechanical (i.e., used spring mechanism [e.g., Smedley Original TTM or Scandidact 281128, Takei TKK 5001]).

[a] China refers to the mainland of China;

[b] Although raw data were available, body position was not coded at the individual-level. However, because standing was preferred and published materials indicated that most participants were measured in standing, HGS data were coded as ‘standing’ before statistical adjustment;

[c] Abducted shoulder position was ‘slight’ (i.e., <45°) and was considered to be practically ‘adducted’ with no statistical adjustment for shoulder position;

[d] While descriptive data were published for each hand separately, because we did not have access to raw data, we used only descriptive HGS data for a single hand (right [41,107,119,141,153] or dominant [116,144]) and then performed statistical adjustments;

[e] Because 2 or more reps per hand were performed, we coded as ‘2 reps per hand’ before statistical adjustment because the 2-rep protocol was more common;

[f] With raw data available and body position coded at the individual-level, we separately calculated descriptive HGS data for those measured in standing (preferred) and seated before statistical adjustment;

[g] With raw data available and testing hand coded at the individual-level, we separately calculated descriptive HGS data for those measured with each hand or only the dominant hand before statistical adjustment;

[h] With raw data available and reps per hand coded at the individual-level, we separately calculated descriptive HGS data for those measured 2 or 3 reps per hand before statistical adjustment;

[i] With only descriptive data available and HGS calculated as the average of the maximum values for each hand between 2000 and 2012, and raw data available and HGS calculated as the maximum irrespective of hand from 2013 onwards, we separately calculated descriptive HGS data as the average of maxima value (i.e., the average of the maximum values for each hand) or the maximum value irrespective of hand before statistical adjustment. Supplement 3, Tables S3A–D show the corresponding adjustment factors and Supplementary Table SE summarizes the adjustments for the included studies.

Abbreviations: HGS = handgrip strength; Min = minute; NS = Not specified (i.e., not reported or controlled). Studies: ASPREE = ASPirin in Reducing Events in the Elderly study; CHARLS = China Health and Retirement Longitudinal Study; CHMS = Canadian Health Measures Survey; CLSA = Canadian Longitudinal Study on Ageing; CNSAF = Chinese National Survey on Adults' Fitness; COmPLETE = CardiO-PuLmonary Exercise Testing health study; DBCS = Danish Birth Cohort Studies of 1905, 1910, 1911, and 1915; DTR (LSADT, MADT, MIDT) = Danish Twin Registry (Longitudinal Study of Aging Danish Twins, Middle Age Danish Twins, MIddle age Danish Twins); ECHA = European Challenge for Healthy Aging study; EDOC = Estudo das Doenças Crônicas (English translation: Study of Chronic Diseases); ELSA = English Longitudinal Study of Ageing; ELSI-Brazil = Brazilian Longitudinal Study of Aging; ENSIN = Encuesta Nacional de la Situacion Nutricional en Colombia (English translation: 2015 National Nutritional Survey); EPIC-Norfolk = European Prospective Investigation into Cancer-Norfolk study; Fibra-BR = Frailty in Brazilian Older People study; FORMoSA = Bavarian Research Foundation-Sarcopenia and Osteoporosis study; FYSIOPRIM = Research Program for Physiotherapy in Primary Health Care study; GenoFit = GenoFit study; GOS = Geelong Osteoporosis Study; HAPIEE = Health, Alcohol and Psychosocial factors In Eastern Europe study; HCS = Hertfordshire Cohort Study; Health2006 = Health2006 study; HRS = Health and Retirement Study; ILAS = I-Lan Longitudinal Aging Study; KNHANES = Korea National Health and Nutrition Examination Survey; KORA-Age = Kooperative Gesundheitsforschung in der Region Augsburg (English translation: Cooperative Health Research in the Region of Augsburg study); KYH = Know Your Heart study; LASI = Longitudinal Ageing Study in India; Lookup 7+ = Longevity check-up 7+ study; MCST NSPF = Ministry of Culture, Sports and Tourism's National Survey of Physical Fitness; Me, Human = Me, Human project; MELoR = Malaysian Elders Longitudinal Research study; MEXT SPFMA = Ministry of Education, Culture, Sports, Science and Technology's Survey on Physical Fitness and Motor Abilities; MrOS = Osteoporotic Fractures in Men (Sweden cohort) study; MSK-FIT = Musculoskeletal, Function, Imaging, and Tissue Resource Core; NAKO = NAKO Gesundheitsstudie (English translation: German National Cohort); NHANES = National Health and Nutrition Examination Survey; NIH Toolbox = U.S. National Institutes of Health Toolbox study; NILS-LSA = National Institute for Longevity Sciences-Longitudinal Study of Aging; NUP65 = Nutrition UP 65 study; NWAHS = North West Adelaide Health Study; OA = Outdoor Active study; Pizzara = Pizzara study; PFSMR = Physical Fitness Study of Macao SAR Residents; PLSAW = Perth Longitudinal Study of Ageing Women; PNAFS = Nutrition, Physical Activity and Health Survey; PURE = Prospective Urban Rural Epidemiology study; ROAD = Research on Osteoarthritis/Osteoporosis Against Disability study; SABE = SAlud, Bienestar, y Envejecimiento (English translation: Survey on Health, Well-Being, and Aging in Latin America and the Caribbean); SAGE = World Health Organization Study on global AGEing and adult health; SAHR = Study of Stress, Aging, and Health in Russia; SANSCOG = Srinivaspura NeuoSenescence, and COGnition study; Saúde-AC = English translation: Health-Antônio Carlos (Santa Catarina) study; SHARE = Survey of Health, Ageing and Retirement in Europe; SIHLS = Social Isolation, Health, and Lifestyles Survey; SONIC = Septuagenarians, Octogenarians, Nonagenarians Investigation with Centenarians study; SOEP = German Socio-Economic Panel survey; TILDA = The Irish Longitudinal Study on Ageing; TMIG-LISA (HaCS, ICS, KLS, NCS, YLS) = Tokyo Metropolitan Institute of Gerontology-Longitudinal Interdisciplinary Study on Aging (Hatoyama Cohort Study, Itabashi Cohort Studies of 2002 and 2011, Kusatsu Longitudinal Study, Nangai Cohort Study, Yoita Longitudinal Study); Tromsø = Norwegian Tromsø study; Twenty-07 = West of Scotland Twenty-07 study; UKHLS = Understanding Society: The UK Household Longitudinal Study; WiSE = Well-being of Singapore Elderly study; Yishun = Yishun study.

Supplementary Table 5. Study quality scores based on the *standard quality assessment criteria for evaluating primary research papers from a variety of fields* tool.

Study	1. Question or objective sufficiently described?	2. Design evident and appropriate to answer study question? (If the study question is not given, infer from the conclusions)	3. Method of subject selection (and comparison group selection, if applicable) or source of information/input variables (e.g., for decision analysis) is described and appropriate.	4. Subject (and comparison group, if applicable) characteristics or input variables/information (e.g., for decision analyses) sufficiently described?	5. If random allocation to treatment group was possible, is it described?	6. If interventional and blinding of investigators to intervention was possible, is it reported?	7. If interventional and blinding of subjects to intervention was possible, is it reported?	8. Outcome and (if applicable) exposure measure(s) well defined and robust to measurement/misclassification bias? Means of assessment reported?	9. Sample size appropriate?	10. Analysis described and appropriate?	11. Some estimate of variance (e.g., confidence intervals, standard errors) is reported for the main results/outcomes (i.e., those directly addressing the study question/ objective upon which the conclusions are based)?	12. Controlled for confounding?	13. Results reported in sufficient detail?	14. Do the results support the conclusions?	Score
1000 Norms Project [3]	1	2	1	1	NA	NA	NA	0	2	1	1	NA	1	2	12
Afable et al. [121]	2	2	1	1	NA	NA	NA	1	2	2	2	NA	2	2	17
Alqahtani et al. [134]	2	2	2	2	NA	NA	NA	1	2	2	2	NA	2	2	19
Alrashdan et al. [135]	1	2	1	2	NA	NA	NA	1	2	2	2	NA	2	2	17
ASPREE [4]	2	2	1	2	NA	NA	NA	2	2	2	2	NA	2	2	19
Axelsson et al. [140]	1	1	1	2	NA	NA	NA	2	2	2	2	NA	2	2	17
Bimali et al. [115]	2	2	1	2	NA	NA	NA	2	2	2	2	NA	2	2	19
Bjerregaard et al. [66]	2	2	2	2	NA	NA	NA	1	2	2	2	NA	2	2	19
Budziareck et al. [26]	1	2	1	1	NA	NA	NA	1	2	2	2	NA	1	2	15
CHARLS [43,44]	2	2	2	2	NA	NA	NA	1	2	2	2	NA	2	2	19
CHMS [36–39]	2	2	2	2	NA	NA	NA	2	2	2	2	NA	2	2	20
CLSA [40]	2	2	1	2	NA	NA	NA	1	2	1	2	NA	2	2	17
CNSAF [45]	2	2	2	1	NA	NA	NA	0	2	2	2	NA	2	2	17
COmPLETE [142]	2	2	2	1	NA	NA	NA	1	2	2	2	NA	2	2	18

DBCS (1905, 1910, 1911, 1915) [51–53]	1	1	2	1	NA	NA	NA	1	2	2	2	NA	2	2	16
DTR (LSADT, MADT, MIDT) [51,54]	1	1	2	1	NA	NA	NA	1	2	2	2	NA	2	2	16
ECHA [55]	2	1	1	1	NA	NA	NA	1	2	2	2	NA	1	2	15
EDOC [27]	2	2	1	1	NA	NA	NA	1	2	2	2	NA	2	2	17
ELSA [146,147]	1	1	1	2	NA	NA	NA	1	2	2	0	NA	1	2	13
ELSI-Brazil [28]	2	2	2	2	NA	NA	NA	2	2	2	2	NA	2	2	20
Enríquez-Reyna et al. [110]	2	2	1	2	NA	NA	NA	1	2	1	2	NA	1	2	16
ENSIN [48]	2	2	1	1	NA	NA	NA	1	2	2	2	NA	2	2	17
EPIC-Norfolk [148]	1	2	2	2	NA	NA	NA	2	2	2	2	NA	2	2	19
Fernandes et al. [29]	1	1	1	1	NA	NA	NA	1	2	2	2	NA	2	2	15
Fibra-BR [30]	2	2	1	2	NA	NA	NA	2	2	2	2	NA	2	2	19
FORMoSA [59]	1	1	2	2	NA	NA	NA	1	2	2	2	NA	2	2	17
FYSIOPRIM [117]	1	2	2	2	NA	NA	NA	1	2	2	2	NA	2	2	18
GenoFit [72]	2	2	1	2	NA	NA	NA	1	2	2	2	NA	2	2	18
Gómez-Campos et al. [41]	2	2	1	2	NA	NA	NA	1	2	2	2	NA	2	2	18
GOS [5,6]	2	2	2	2	NA	NA	NA	2	2	2	2	NA	2	2	20
HAPIEE [50]	1	0	2	1	NA	NA	NA	0	2	1	1	NA	0	0	8
HCS [149]	1	2	1	1	NA	NA	NA	1	2	2	2	NA	2	2	16
Health2006 [56]	2	2	2	2	NA	NA	NA	2	2	2	2	NA	2	2	20
Hogrel [58]	2	1	1	1	NA	NA	NA	2	2	2	2	NA	2	2	17
HRS [154,155]	2	1	0	2	NA	NA	NA	1	2	1	2	NA	2	2	15
ILAS [145]	1	2	1	1	NA	NA	NA	1	2	1	2	NA	2	2	15
Ingram et al. [7]	1	1	2	2	NA	NA	NA	2	2	2	2	NA	2	2	18
Kim et al. [116]	1	2	1	1	NA	NA	NA	1	2	0	1	NA	1	2	12

Kjær et al. [118]	2	2	1	1	NA	NA	NA	2	2	2	2	NA	2	2	18
KNHANES [124,125]	2	2	1	1	NA	NA	NA	2	2	2	2	NA	1	2	17
KORA-Age [60]	2	2	2	2	NA	NA	NA	1	2	2	2	NA	2	2	19
KYH [131]	1	2	1	2	NA	NA	NA	2	2	2	2	NA	2	2	18
Lam et al. [107]	2	2	2	2	NA	NA	NA	2	2	2	2	NA	2	2	20
Langer et al. [74]	2	2	1	1	NA	NA	NA	1	2	2	2	NA	2	2	17
LASI [68]	1	2	2	2	NA	NA	NA	2	2	2	2	NA	2	2	19
Leal Cárcamo et al. [42]	2	2	2	1	NA	NA	NA	2	2	2	2	NA	2	2	19
Lookup 7+ [75]	2	1	2	2	NA	NA	NA	1	2	2	2	NA	2	2	18
Malina et al. [111]	1	1	1	1	NA	NA	NA	1	2	1	2	NA	1	2	13
MCST NSPF [126–130]	2	1	1	1	NA	NA	NA	0	2	1	1	NA	1	1	11
Me, Human [150]	2	2	1	1	NA	NA	NA	2	2	2	2	NA	2	2	18
MELoR [108]	1	2	1	2	NA	NA	NA	1	2	1	2	NA	2	2	16
MEXT SPFMA [76–96]	2	2	2	2	NA	NA	NA	2	2	2	2	NA	2	2	20
Mohammadian et al. [70]	2	2	1	1	NA	NA	NA	2	2	2	2	NA	2	2	18
MrOS [141]	2	2	1	2	NA	NA	NA	1	2	1	2	NA	2	2	17
MSK-FIT [156]	2	1	1	2	NA	NA	NA	2	2	2	2	NA	2	2	18
NAKO [60]	2	2	2	2	NA	NA	NA	1	2	2	2	NA	2	2	19
NHANES [157]	1	2	2	2	NA	NA	NA	2	2	2	2	NA	2	2	19
NIH Toolbox [158,159]	2	2	2	2	NA	NA	NA	2	2	2	2	NA	2	2	20
NILS-LSA [97]	1	2	2	1	NA	NA	NA	1	2	2	2	NA	2	2	17
Nilsen et al. [119]	2	2	1	2	NA	NA	NA	1	2	2	2	NA	2	2	18
Núñez-Othón et al. [112]	2	2	2	2	NA	NA	NA	2	2	2	2	NA	2	2	20
NUP65 [123]	2	2	2	2	NA	NA	NA	2	2	2	2	NA	2	2	20

NWAHS [8]	1	1	2	1	NA	NA	NA	1	2	1	2	NA	1	2	14
OA [61]	2	2	2	1	NA	NA	NA	1	2	2	2	NA	2	2	18
PFSMR [102–106]	1	2	2	2	NA	NA	NA	1	2	2	2	NA	2	2	18
Pizzara [139]	2	2	1	2	NA	NA	NA	1	2	2	2	NA	1	2	17
PLSAW [9]	1	2	2	2	NA	NA	NA	1	2	2	2	NA	2	2	18
PNAFS [31]	2	1	2	1	NA	NA	NA	1	2	1	2	NA	1	2	15
PURE [2]	1	2	1	2	NA	NA	NA	2	2	1	1	NA	1	2	15
ROAD [98]	1	2	2	1	NA	NA	NA	1	2	0	2	NA	1	2	14
Rodríguez-García et al. [113,114]	2	2	2	2	NA	NA	NA	1	2	2	2	NA	2	2	19
Rostamzadeh et al. [71]	2	2	2	2	NA	NA	NA	2	2	2	2	NA	2	2	20
SABE [17,18,32]	2	2	2	2	NA	NA	NA	1	2	1	2	NA	2	2	18
SABE [49]	2	2	2	2	NA	NA	NA	1	2	2	2	NA	2	2	19
SAGE [46]	2	2	2	2	NA	NA	NA	1	2	2	2	NA	2	2	19
SAHR [131]	1	2	2	2	NA	NA	NA	2	2	2	2	NA	2	2	19
SANSCOG [69]	2	2	2	1	NA	NA	NA	2	2	1	2	NA	1	2	17
Saúde-AC [33]	2	1	1	1	NA	NA	NA	1	2	2	2	NA	2	2	16
Schilling et al. [62]	2	2	2	2	NA	NA	NA	1	2	2	2	NA	2	2	19
Shah et al. [109]	1	2	1	1	NA	NA	NA	1	2	2	2	NA	1	2	15
Shanghai Administration of Sports [47]	2	2	2	1	NA	NA	NA	0	2	2	2	NA	2	2	17
SHARE [10–16,19–25,34,35]	2	2	2	2	NA	NA	NA	2	2	2	2	NA	2	2	20
SIHLS [136]	2	2	1	2	NA	NA	NA	1	2	2	2	NA	2	2	18
SOEP [63–65]	2	2	2	2	NA	NA	NA	1	2	2	2	NA	2	2	19
SONIC [99]	2	2	1	1	NA	NA	NA	2	2	2	2	NA	2	2	18
Suetta et al. [57]	2	2	2	2	NA	NA	NA	1	2	2	2	NA	2	2	19

Taniguchi et al. [100]	2	2	1	2	NA	NA	NA	1	2	2	2	NA	2	2	18
TILDA [73]	1	2	2	1	NA	NA	NA	1	2	2	2	NA	2	2	17
TMIG-LISA (HaCS, ICS, KLS, NCS, YLS) [101]	2	2	2	2	NA	NA	NA	1	2	2	2	NA	2	2	19
Tromsø [120]	2	2	2	2	NA	NA	NA	1	2	2	2	NA	2	2	19
Turusheva et al. [133]	2	2	1	2	NA	NA	NA	1	2	2	2	NA	2	2	18
Twenty-07 [151]	2	1	2	1	NA	NA	NA	1	2	1	1	NA	1	2	14
UKHLS [152]	2	1	1	2	NA	NA	NA	1	2	2	2	NA	2	2	17
Wearing et al. [143]	2	2	1	2	NA	NA	NA	2	2	2	2	NA	2	2	19
Werle et al. [144]	2	0	0	2	NA	NA	NA	2	2	2	2	NA	2	2	16
WiSE [137]	2	1	2	1	NA	NA	NA	2	2	2	2	NA	2	2	18
Wiśniowska-Szurlej et al. [122]	2	2	1	2	NA	NA	NA	2	2	2	2	NA	2	2	19
Wozny et al. [153]	2	2	2	2	NA	NA	NA	2	2	2	2	NA	2	2	20
Yishun [138]	2	1	1	2	NA	NA	NA	1	2	2	2	NA	2	2	17
Yu et al. [67]	2	2	2	2	NA	NA	NA	2	2	2	2	NA	2	2	20

Notes: Some studies (e.g., HAPIEE [50], MCST NSPF [126–130]) were protocol papers or technical reports and were therefore scored lower because they did not satisfy several of the quality assessment criteria.

Abbreviations*:* NA = Not applicable; Studies: ASPREE = ASPirin in Reducing Events in the Elderly study; CHARLS = China Health and Retirement Longitudinal Study; CHMS = Canadian Health Measures Survey; CLSA = Canadian Longitudinal Study on Ageing; CNSAF = Chinese National Survey on Adults' Fitness; COmPLETE = CardiO-PuLmonary Exercise Testing health study; DBCS = Danish Birth Cohort Studies of 1905, 1910, 1911, and 1915; DTR (LSADT, MADT, MIDT) = Danish Twin Registry (Longitudinal Study of Aging Danish Twins, Middle Age Danish Twins, MIddle age Danish Twins); ECHA = European Challenge for Healthy Aging study; EDOC = Estudo das Doenças Crônicas (English translation: Study of Chronic Diseases); ELSA = English Longitudinal Study of Ageing; ELSI-Brazil = Brazilian Longitudinal Study of Aging; ENSIN = Encuesta Nacional de la Situacion Nutricional en Colombia (English translation: 2015 National Nutritional Survey); EPIC-Norfolk = European Prospective Investigation into Cancer-Norfolk study; Fibra-BR = Frailty in Brazilian Older People study; FORMoSA = Bavarian Research Foundation-Sarcopenia and Osteoporosis study; FYSIOPRIM = Research Program for Physiotherapy in Primary Health Care study; GenoFit = GenoFit study; GOS = Geelong Osteoporosis Study; HAPIEE = Health, Alcohol and Psychosocial factors In Eastern Europe study; HCS = Hertfordshire Cohort Study; Health2006 = Health2006 study; HRS = Health and Retirement Study; ILAS = I-Lan Longitudinal Aging Study; KNHANES = Korea National Health and Nutrition Examination Survey; KORA-Age = Kooperative Gesundheitsforschung in der Region Augsburg (English translation: Cooperative Health Research in the Region of Augsburg study); KYH = Know Your Heart study; LASI = Longitudinal Ageing Study in India; Lookup 7+ = Longevity check-up 7+ study; MCST NSPF = Ministry of Culture, Sports and Tourism's National Survey of Physical Fitness; Me, Human = Me, Human project; MELoR = Malaysian Elders Longitudinal Research study; MEXT SPFMA = Ministry of Education, Culture, Sports, Science and Technology's Survey on Physical Fitness and Motor Abilities; MrOS = Osteoporotic Fractures in Men (Sweden cohort) study; MSK-FIT = Musculoskeletal, Function, Imaging, and Tissue Resource Core; NAKO = NAKO Gesundheitsstudie (English translation:

German National Cohort); NHANES = National Health and Nutrition Examination Survey; NIH Toolbox = U.S. National Institutes of Health Toolbox study; NILS-LSA = National Institute for Longevity Sciences-Longitudinal Study of Aging; NUP65 = Nutrition UP 65 study; NWAHS = North West Adelaide Health Study; OA = Outdoor Active study; Pizzara = Pizzara study; PFSMR = Physical Fitness Study of Macao SAR Residents; PLSAW = Perth Longitudinal Study of Ageing Women; PNAFS = Nutrition, Physical Activity and Health Survey; PURE = Prospective Urban Rural Epidemiology study; ROAD = Research on Osteoarthritis/Osteoporosis Against Disability study; SABE = SAlud, Bienestar, y Envejecimiento (English translation: Survey on Health, Well-Being, and Aging in Latin America and the Caribbean); SAGE = World Health Organization Study on global AGEing and adult health; SAHR = Study of Stress, Aging, and Health in Russia; SANSCOG = Srinivaspura NeuoSenescence, and COGnition study; Saúde-AC = English translation: Health-Antônio Carlos (Santa Catarina) study; SHARE = Survey of Health, Ageing and Retirement in Europe; SIHLS = Social Isolation, Health, and Lifestyles Survey; SONIC = Septuagenarians, Octogenarians, Nonagenarians Investigation with Centenarians study; SOEP = German Socio-Economic Panel survey; TILDA = The Irish Longitudinal Study on Ageing; TMIG-LISA (HaCS, ICS, KLS, NCS, YLS) = Tokyo Metropolitan Institute of Gerontology-Longitudinal Interdisciplinary Study on Aging (Hatoyama Cohort Study, Itabashi Cohort Studies of 2002 and 2011, Kusatsu Longitudinal Study, Nangai Cohort Study, Yoita Longitudinal Study); Tromsø = Norwegian Tromsø study; Twenty-07 = West of Scotland Twenty-07 study; UKHLS = Understanding Society: The UK Household Longitudinal Study; WiSE = Well-being of Singapore Elderly study; Yishun = Yishun study.

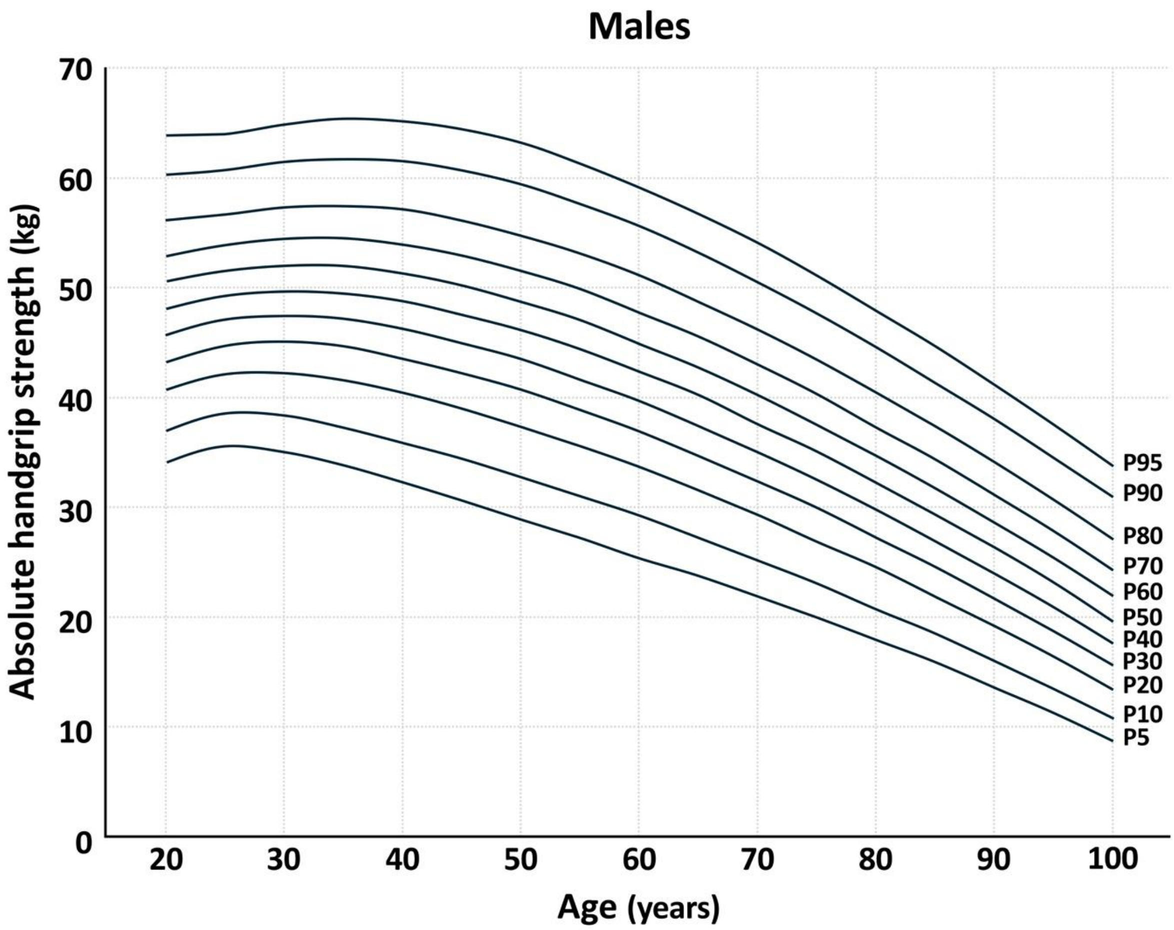

Supplementary Fig. 2A. Smoothed percentile curves (P_5 to P_{95}) for absolute handgrip strength in kilograms (kg) for males aged 20 to 100+ years.

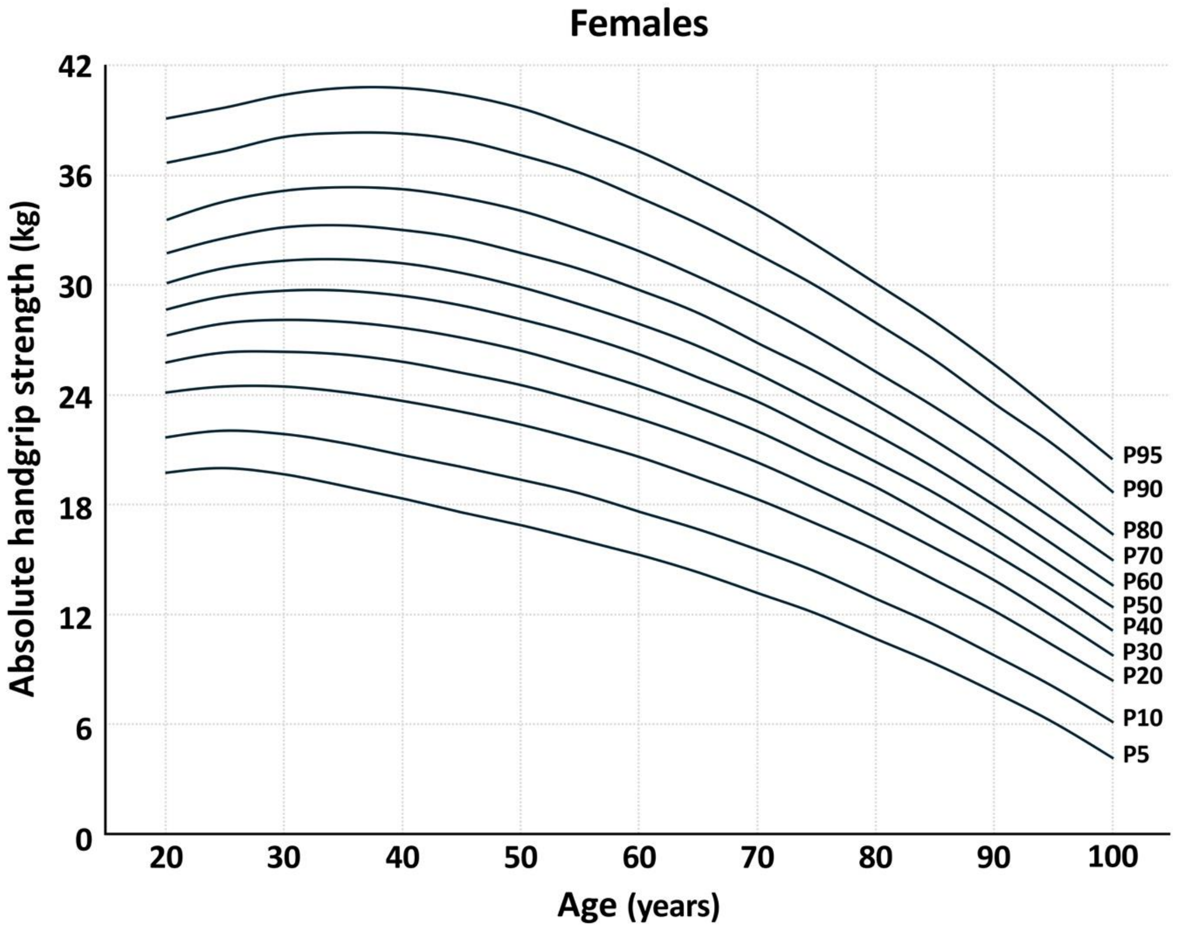

Supplementary Fig. 2B. Smoothed percentile curves (P_5 to P_{95}) for absolute handgrip strength in kilograms (kg) for females aged 20 to 100+ years.

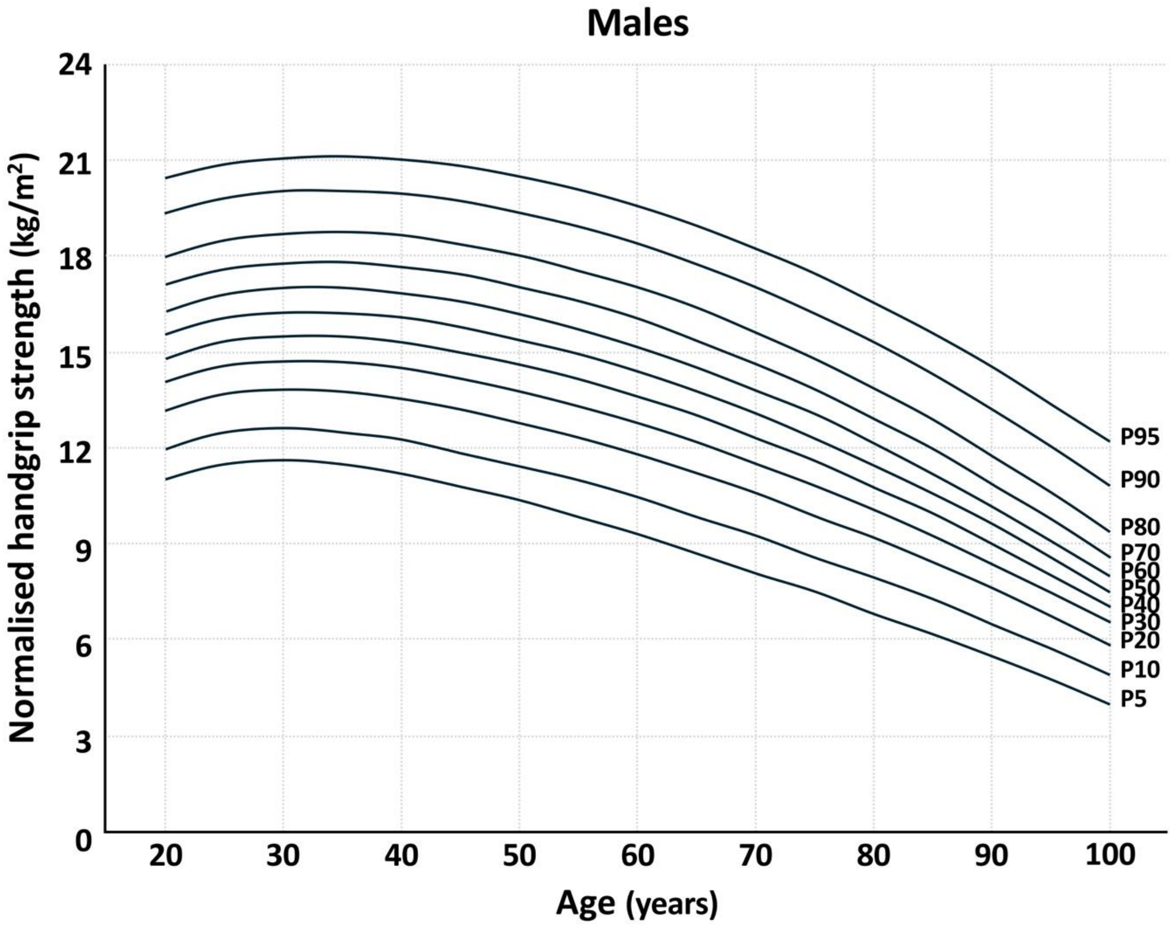

Supplementary Fig. 2C. Smoothed percentile curves (P_5 to P_{95}) for normalized handgrip strength (handgrip strength in kilograms divided by height in meters squared [kg/m^2]) for males aged 20 to 100+ years.

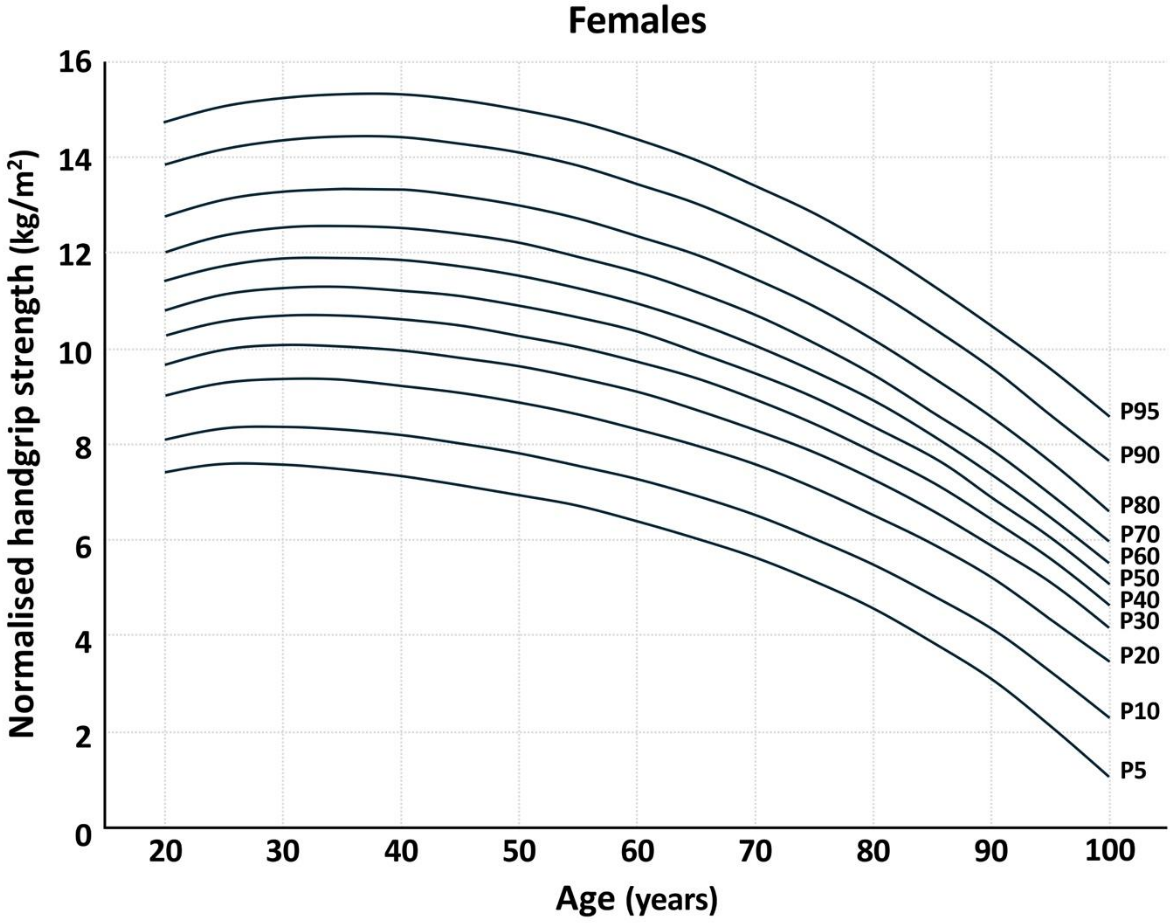

Supplementary Fig. 2D. Smoothed percentile curves (P_5 to P_{95}) for normalized handgrip strength (handgrip strength in kilograms divided by height in meters squared [kg/m^2]) for females aged 20 to 100+ years.

Supplementary Funding

We would like to acknowledge the funding received by the following group authors:

KB, AW: Institute for Sport & Sport Science, Karlsruhe, Germany.

CC: UK Medical Research Council (MC_PC_21003; MC_PC_21001).

MCER: Consejo Nacional de Ciencia y Tecnología.

NCH: UK Medical Research Council (MC_PC_21003; MC_PC_21001) and NIHR Southampton Biomedical Research Centre, UK.

MS: Royal Perth Hospital Career Advancement Fellowship and an Emerging Leader Fellowship from the Western Australian Future Health Research and Innovation Fund.

SJW: National Institutes of Health (NIH/NIAMS P30 AR072581) and the Indiana Clinical Translational Science Award/Institute (NCATS UL1TR002529-01).

LDW: UK Medical Research Council (MC_PC_21003; MC_PC_21001).

We would like to acknowledge the funding received by the following included studies (see Supplementary Table 4A and 4B for study and protocol details and Supplementary References for the full citations):

1000 Norms Project: Funded by National Health and Medical Research Council (NHMRC) of Australia Centre for Research Excellence in Neuromuscular Disorders (NHMRC 1031893) and the Australian Podiatry Education and Research Foundation.

Alqahtani et al. [134]: Funded by the Prince Sattam Bin Abdulaziz University (PSAU/2023/R/1444).

ASPREE (ASPirin in Reducing Events in the Elderly study): ASPREE was supported by a grant (U01AG029824) from the National Institute on Aging and the National Cancer Institute at the National Institutes of Health, by grants (334047 and 1127060) from the National Health and Medical Research Council (NHMRC) of Australia, and by Monash University and the Victorian Cancer Agency.

CHARLS (China Health and Retirement Longitudinal Study): Supported by the Behavioral and Social Research division of the National Institute on Aging of the National Institute of Health (Grants: 1-R21-AG031372-01, 1-R01-AG037031-01, and 3-R01AG037031-03S1), the Natural Science Foundation of China (Grants: 70773002, 70910107022, and 71130002), the World Bank (Contracts: 7145915 and 7159234), and Peking University.

CLSA (Canadian Longitudinal Study on Aging): Funding for the CLSA is provided by the Government of Canada through the Canadian Institutes of Health Research under grant

reference: LSA 9447 and the Canada Foundation for Innovation, as well as the following provinces: Newfoundland, Nova Scotia, Quebec, Ontario, Manitoba, Alberta, and British Columbia. The CLSA is led by Drs Parminder Raina, Christina Wolfson and Susan Kirkland.

COmPLETE (CardiO-PuLmonary Exercise Testing health study): Funded by the Swiss National Science Foundation (182815).

EDOC (Estudo das Doenças Crônicas (English translation: Study of Chronic Diseases)): Funded by the National Council for Scientific and Technological Development—CNPq (Call MCTI/CNPQ/ MS-SCTIE-DECIT n. 06/2013, for Health System by the Brazilian Network for Health Technology Assessment (REBRATS)). Acre Research Foundation—FAPAC (Calls PPSUS n. 001/2013 and n. 001/2015 of the Research Program for SUS: shared health management (MS/CNPq/ FAPAC/SESACRE).

ELSA (English Longitudinal Study of Ageing): Funded by the National Institute on Aging (Ref: R01AG017644) and by a consortium of UK government departments: Department for Health and Social Care; Department for Transport; Department for Work and Pensions, which is coordinated by the National Institute for Health Research (NIHR, Ref: 198-1074). Funding has also been provided by the Economic and Social Research Council (ESRC).

ELSI-Brazil (Brazilian Longitudinal Study of Aging): Supported by the Brazilian Ministry of Health: DECIT/SCTIE (Grants: 404965/2012-1 and TED 28/2017); COPID/DECIV/SAPS (Grants: 20836, 22566, 23700, 25560, 25552, and 27510).

EPIC-Norfolk (European Prospective Investigation into Cancer-Norfolk study): The EPIC-Norfolk study (DOI 10.22025/2019.10.105.00004) was funded by the Medical Research Council (MR/N003284/1 MC-UU_12015/1 and MC_UU_00006/1) and Cancer Research UK (C864/A14136). The genetics work was funded by the Medical Research Council (MC_PC_13048). We are grateful to all the participants who have been part of the project and to the many members of the study teams at the University of Cambridge who have enabled this research.

GOS (Geelong Osteoporosis Study): Funded by The Victorian Health Promotion Foundation (ID 91-0095) and the National Health and Medical Research Council (NHMRC) of Australia (ID 251638, 299831, 628582).

GenoFit: Supported by Genuity Science, University College Dublin and the Irish Research Council.

HAPIEE (Health, Alcohol and Psychosocial factors In Eastern Europe study): Funded by Wellcome Trust "Determinants of cardiovascular diseases in Eastern Europe:

Longitudinal follow-up of a multi-centre cohort study" (The HAPIEE Project) (081081/Z/06/Z).

HCS (Hertfordshire Cohort Study): Funded by the Medical Research Council University Unit Partnership (MRC_MC_UP_A620_1014).

Hogrel [58]: Supported in part by the Association Française contre les Myopathies (AFM).

HRS (Health and Retirement Study): Sponsored by the National Institute on Aging (grant number NIA U01AG009740) and conducted by the University of Michigan.

Kjær et al. [118]: Funded and initiated by the Norwegian Directorate of Health, with the Norwegian School of Sport Sciences leading the study and contributing funding. We are grateful toward the cooperative test centers involved in this larger study: Finnmark University College, Hedmark University College, Norwegian University of Science and Technology Social research, Sogn og Fjordane University College, University of Agder, University of Nordland, University of Stavanger, Telemark University College, and the Norwegian School of Sport Sciences.

KORA-Age (Kooperative Gesundheitsforschung in der Region Augsburg (English translation: Cooperative Health Research in the Region of Augsburg study)): Initiated and funded by the Helmholtz Zentrum München—German Research Center for Environmental Health, which is funded by the German Federal Ministry of Education and Research (BMBF) and by the State of Bavaria. Data collection in the KORA study was done in cooperation with the University Hospital of Augsburg. The KORA-Age project was financed by the German Federal Ministry of Education and Research (BMBF FKZ 01ET0713 and 01ET1003A) as part of the "Health in old age" program. We thank all participants for their long-term commitment to the KORA study, the staff for data collection and research data management, and the members of the KORA Study Group (https://www.helmholtz-munich.de/en/epi/cohort/kora), who are responsible for the design and conduct of the study.

LASI (Longitudinal Ageing Study in India): Funded by the Ministry of Health and Family Welfare, Government of India, the National Institute on Aging (R01 AG042778), and United Nations Population Fund, India.

MELoR (Malaysian Elders Longitudinal Research): This is now part of the Transforming Cognitive Frailty into Later-Life Self-Sufficiency (AGELESS) study, which is funded by the Malaysian Ministry of Higher Education (MOHE) Long Term Research Grant Scheme (LRGS/1/2019/UM//1/1) and was also funded by a MOHE High Impact Research fund (UM.C/625/1/HIR/MOHE/ASH/02).

MSK-FIT (Musculoskeletal, Function, Imaging, and Tissue Resource Core): National Institutes of Health (NIH/NIAMS P30 AR072581) and the Indiana Clinical Translational Science Award/Institute (NCATS UL1TR002529-01).
NAKO (NAKO Gesundheitsstudie (English translation: German National Cohort)): Conducted with data from the German National Cohort (NAKO) (www.nako.de). Funded by the Federal Ministry of Education and Research (BMBF) (01ER1301A/B/C, 01ER1511D, and 01ER1801A/B/C/D), federal states of Germany and the Helmholtz Association, the participating universities and the institutes of the Leibniz Association. We thank all participants who took part in the NAKO study and the staff of this research initiative.
PLSAW (Perth Longitudinal Study of Ageing Women): Funded by Healthway, the Western Australian Health Promotion Foundation, and by project grants 254627, 303169, and 572604 from the National Health and Medical Research Council (NHMRC) of Australia.
PNAFS (Nutrition, Physical Activity, and Health Survey): Funded by the Brazilian National Research Council (CNPq; grants 471172/2001-4 and 475122/2003-8) and the Oswaldo Cruz Foundation (PAPES III e Program to Support Strategic Projects in Health, no. 250.139). The authors received research productivity grants from CNPq (301076/89-8, 302992/2003-0, and 306523/2006-0).
SANSCOG (Srinivaspura NeuoSenescence, and COGnition study): Funded by the Centre for Brain Research, Indian Institute of Science, India. The funding source did not have any role in the study design; in the collection, analysis, and interpretation of data; in the writing of the manuscript; or in the decision to submit the article for publication.
Shah et al. [109]: Funded by the World Health Organization Centre for Health Development (WHO Kobe Centre, WKC: K18015).
SHARE (Survey of Health, Ageing and Retirement in Europe): This paper uses data from SHARE Waves 1, 2, 4, 5, 6, and 7 (DOIs: 10.6103/SHARE.w1.900, 10.6103/SHARE.w2.900, 10.6103/SHARE.w4.900, 10.6103/SHARE.w5.900, 10.6103/SHARE.w6.900, and 10.6103/SHARE.w7.900) see Börsch-Supan et al. (2013) for methodological details. The SHARE data collection has been funded by the European Commission, DG RTD through FP5 (QLK6-CT-2001-00360), FP6 (SHARE-I3: RII-CT-2006-062193, COMPARE: CIT5-CT-2005-028857, SHARELIFE: CIT4-CT-2006-028812), FP7 (SHARE-PREP: GA N°211909, SHARE-LEAP: GA N°227822, SHARE M4: GA N°261982, DASISH: GA N°283646) and Horizon 2020 (SHARE-DEV3: GA N°676536, SHARE-COHESION: GA N°870628, SERISS: GA N°654221, SSHOC: GA N°823782, SHARE-COVID19: GA N°101015924) and by DG Employment, Social Affairs & Inclusion

through VS 2015/0195, VS 2016/0135, VS 2018/0285, VS 2019/0332, VS 2020/0313, and SHARE-EUCOV: GA N°101052589 and EUCOVII: GA N°101102412. Additional funding from the German Ministry of Education and Research, the Max Planck Society for the Advancement of Science, the US National Institute on Aging (U01_AG09740-13S2, P01_AG005842, P01_AG08291, P30_AG12815, R21_AG025169, Y1-AG-4553-01, IAG_BSR06-11, OGHA_04-064, BSR12-04, R01_AG052527-02, HHSN271201300071C, RAG052527A), and from various national funding sources is gratefully acknowledged (see www.share-eric.eu).

SIHLS (Social Isolation, Health, and Lifestyles Survey): Funded by the Ministry of Social and Family Development. Development of handgrip strength normative values based on data from the survey was supported by the "Estate of Tan Sri Khoo Teck Puat" and the National University of Singapore (Khoo Student Research Award). It was also supported by a grant obtained by the Nihon University Population Research Institute from the "Academic Frontier" Project for Private Universities: matching fund subsidy from MEXT (Ministry of Education, Culture, Sports, Science and Technology), 2006–2010.

TILDA (The Irish Longitudinal Study on Ageing): Funded by the Irish Government, the Health Research Board (HRB) and the Atlantic Philanthropies. We would like to thank the TILDA participants, without whom this research would not be possible.

Twenty-07 (West of Scotland Twenty-07 study): Funded by the UK Medical Research Council. Data originally collected by the MRC Social and Public Health Sciences Unit (MC_A540_53462). Supported by the MRC-funded Inequalities in Health programmes (MC_UU_00022/2) and the Chief Scientist Office (CSO) (SPHSU17). We are grateful to all of the participants in the study and to the survey staff and research nurses who carried it out.

UKHLS (Understanding Society: The UK Household Longitudinal Study): Funded by the Economic and Social Research Council (ESRC).

Supplementary References

1 Nevill AM, Tomkinson GR, Lang JJ, Wutz W, Myers TD. How should adult handgrip strength be normalized? Allometry reveals new insights and associated reference curves. *Med Sci Sports Exerc 2022*;54(1):162–168. doi: 10.1249/MSS.0000000000002771.

2 Leong DP, Teo KK, Rangarajan S, Kutty R, Lanas F, Hui C, Quanyong X, Zhenzhen Q, Jinhua T, Noorhassim I, AlHabib KF, Moss SJ, Rosengren A, Akalin AA, Rahman O, Chifamba J, Orlandini A, Kumar R, Yeates K, Gupta R, Yusufali A, Dans A, Avezum A, Lopez-Jaramillo P, Poirier P, Heidari H, Zatonska K, Iqbal R, Khatib R, Yusuf S. Reference ranges of handgrip strength from 125,462 healthy adults in 21 countries: A prospective urban rural epidemiologic (PURE) study. *J Cachexia Sarcopenia Muscle* 2016;7(5):535–546. doi: 10.1002/jcsm.12112.

3 McKay MJ, Baldwin JN, Ferreira P, Simic M, Vanicek N, Burns J, 1000 Norms Project Consortium. Normative reference values for strength and flexibility of 1,000 children and adults. *Neurology* 2017;88(1):36–43. doi: 10.1212/WNL.0000000000003466.

4 Wu Z, Woods RL, Chong TTJ, Orchard SG, Shah RC, Wolfe R, Storey E, Sheets KM, Murray AM, McNeil JJ, Ryan J. Grip strength, gait speed, and trajectories of cognitive function in community-dwelling older adults: A prospective study. *Alzheimers Dement (Amst) 2023*;15(1):e12388. doi: 10.1002/dad2.12388.

5 Sui SX, Holloway-Kew KL, Hyde NK, Williams LJ, Tembo MC, Mohebbi M, Gojanovic M, Leach S, Pasco JA. Handgrip strength and muscle quality in Australian women: Cross-sectional data from the Geelong Osteoporosis Study. *J Cachexia Sarcopenia Muscle 2020*;11(3):690–697. doi: 10.1002/jcsm.12544.

6 Pasco JA, Sui SX, West EC, Holloway-Kew KL, Hyde NK, Stuart AL, Gaston J, Williams LJ. Operational definitions of sarcopenia should consider depressive symptoms. *JCSM Clinical Reports 2021*;6(2):62–68. doi: 10.1002/crt2.32.

7 Ingram LA, Butler AA, Walsh LD, Brodie MA, Lord SR, Gandevia SC. The upper limb Physiological Profile Assessment: Description, reliability, normative values and criterion validity. *PLoS One 2019*;14(6):e0218553. doi: 10.1371/journal.pone.0218553.

8 Massy-Westropp NM, Gill TK, Taylor, AW, Bohannon RW, Hill CL Hand grip strength: Age and gender stratified normative data in a population-based study. *BMC Res Notes* 2011;4:127. doi: 10.1186/1756-0500-4-127.

9 Sim M, Prince RL, Scott D, Daly RM, Duque G, Inderjeeth CA, Zhu K, Woodman RJ, Hodgson JM, Lewis JR. Sarcopenia definitions and their associations with mortality in

older Australian women. *J Am Med Dir Assoc 2019*;20(1):76–82.e2. doi: 10.1016/j.jamda.2018.10.016.

10 Börsch-Supan A, Brandt M, Hunkler C, Kneip T, Korbmacher J, Malter F, Schaan B, Stuck S, Zuber S; SHARE Central Coordination Team. Data resource profile: The Survey of Health, Ageing and Retirement in Europe (SHARE). *Int J Epidemiol 2013*;42(4):992–1001. doi: 10.1093/ije/dyt088.

11 SHARE-ERIC (2024). Survey of Health, Ageing and Retirement in Europe (SHARE) Wave 1. Release version: 9.0.0. SHARE-ERIC. Data set. doi: 10.6103/SHARE.w1.900. [accessed 09.02.2023].

12 Börsch-Supan A, Brugiavini A, Jürges H, Mackenbach J, Siegrist J, Weber G. *Health, ageing and retirement in Europe—First results from the Survey of Health, Ageing and Retirement in Europe.* Mannheim: Mannheim Research Institute for the Economics of Aging (MEA); 2005.

13 Börsch-Supan A, Jürges H (Eds.). *The Survey of Health, Ageing and Retirement in Europe—Methodology.* Mannheim: Mannheim Research Institute for the Economics of Aging (MEA); 2005.

14 SHARE-ERIC (2024). Survey of Health, Ageing and Retirement in Europe (SHARE) Wave 4. Release version: 9.0.0. SHARE-ERIC. Data set. doi: 10.6103/SHARE.w4.900. [accessed 09.02.2023].

15 Börsch-Supan A, Brandt M, Litwin H, Weber G (Eds). *Active ageing and solidarity between generations in Europe: First results from SHARE after the economic crisis*. Berlin: De Gruyter; 2013.

16 Malter F, Börsch-Supan A. (Eds.). *SHARE Wave 4: Innovations & Methodology*. Munich: MEA, Max Planck Institute for Social Law and Social Policy; 2013.

17 Pelaez M, Palloni A, Albala C, Alfonso JC, Ham-Chande R, Hennis A, Lebrao ML, Lesn-Diaz E, Pantelides E, Prats O. *SABE—Survey on Health, Well-Being, and Aging in Latin America and the Caribbean, 2000.* Ann Arbor, MI: Inter-university Consortium for Political and Social Research [distributor], 2006. https://doi.org/10.3886/ICPSR03546.v1. (see https://www.icpsr.umich.edu/web/NACDA/studies/3546). [accessed 16.05.2023].

18 Rodrigues-Barbosa A, de Miranda LM, Vieira-Guimarães A, Xavier-Corseuil H, Weber-Corseuil M. Age and gender differences regarding physical performance in the elderly from Barbados and Cuba. *Rev Salud Publica (Bogota) 2011*;13(1):54–66.

19 SHARE-ERIC. *Survey of Health, Ageing and Retirement in Europe (SHARE) Wave 2*. Release version: 9.0.0. SHARE-ERIC Data set; 2024. doi: 10.6103/SHARE.w2.900. [accessed 09.02.2023].

20 Börsch-Supan A, Brugiavini A, Jürges H, Kapteyn A, Mackenbach J, Siegrist J, Weber G. First results from the Survey of Health, Ageing and Retirement in Europe (2004-2007)—Starting the longitudinal dimension. Mannheim: Mannheim Research Institute for the Economics of Aging (MEA); 2008.

21 SHARE-ERIC. *Survey of Health, Ageing and Retirement in Europe (SHARE) Wave 5.* Release version: 9.0.0. SHARE-ERIC. Data set; 2024. doi: 10.6103/SHARE.w5.900. [accessed 09.02.2023].

22 Börsch-Supan A, Kneip T, Litwin H, Myck M, Weber G. (Eds.). A*geing in Europe—Supporting policies for an inclusive society.* Berlin: De Gruyter; 2015.

23 Malter F, Börsch-Supan A. (Eds.). SHARE Wave 5: Innovations & methodology. Munich: MEA, Max Planck Institute for Social Law and Social Policy; 2015.

24 SHARE-ERIC. *Survey of Health, Ageing and Retirement in Europe (SHARE) Wave 6.* Release version: 9.0.0. SHARE-ERIC. Data set; 2024. doi: 10.6103/SHARE.w6.900. [accessed 09.02.2023].

25 Malter F, Börsch-Supan A. (Eds.). *SHARE Wave 6: Panel innovations and collecting dried blood spots.* Munich: Munich Center for the Economics of Aging (MEA); 2017.

26 Budziareck MB, Duarte RRP, Barbosa-Silva MCG. Reference values and determinants for handgrip strength in healthy subjects. *Clin Nutr 2008*;27(3):357–362. doi: 10.1016/j.clnu.2008.03.008.

27 Amaral CA, Amaral TLM, Monteiro GTR, Vasconcellos MTL, Portela MC. Hand grip strength: Reference values for adults and elderly people of Rio Branco, Acre, Brazil. *PLoS One 2019*;14(1):e0211452. doi: 10.1371/journal.pone.0211452.

28 de Souza Moreira B, de Souza Andrade AC, Torres JL, de Souza Braga L, de Carvalho Bastone A, de Melo Mambrini JV, Lima-Costa MF. Nationwide handgrip strength values and factors associated with muscle weakness in older adults: Findings from the Brazilian Longitudinal Study of Aging (ELSI-Brazil). *BMC Geriatr 2022*;22(1):1005. doi: 10.1186/s12877-022-03721-0.

29 Fernandes AA, Natali AJ, Vieira BC, Neves do Valle MAA, Moreira DG, Massy-Westropp N, Marins JCB. The relationship between hand grip strength and anthropometric parameters in men. *Arch Med Deporte* 2014;31(3):160–164.

30 Reichenheim ME, Lourenço RA, Nascimento JS, Moreira VG, Neri AL, Ribeiro RM, Lustosa LP, Ferriolli E. Normative reference values of handgrip strength for Brazilian older people aged 65 to 90 years: Evidence from the multicenter Fibra-BR study. *PLoS One* 2021;16(5):e0250925. doi: 10.1371/journal.pone.0250925.

31 Schlüssel MM, dos Anjos LA, de Vasconcellos MTL, Kac G. Reference values of handgrip dynamometry of healthy adults: A population-based study. *Clin Nutr 2008*;27(4):601–607. doi: 10.1016/j.clnu.2008.04.004.

32 Barbosa AR, Souza JMP, Lebrão ML, Laurenti R, Marucci MDFN. Functional limitations of Brazilian elderly by age and gender differences: Data from SABE Survey. *Cad Saúde Pública 2005*;21(4):1177–1185. doi: 10.1590/s0102-311x2005000400020.

33 Confortin SC, Barbosa AR, Danielewics AL, Meneghini V, Testa WL. Desempenho motor de idosos de uma comunidade do sul do Brasil (Motor performance of elderly in a community in southern Brazil). *Rev Bras Cineantropom Desempenho Hum 2013*;15(4):417–426. doi: 10.5007/1980-0037.2013v15n4p417.

34 SHARE-ERIC. *Survey of Health, Ageing and Retirement in Europe (SHARE) Wave 7.* Release version: 9.0.0. SHARE-ERIC. Data set; 2024. doi: 10.6103/SHARE.w7.900. [accessed 09.02.2023].

35 Bergmann M, Scherpenzeel A, Börsch-Supan A. (Eds.) *SHARE Wave 7 Methodology: Panel innovations and life histories.* Munich: Munich Center for the Economics of Aging (MEA); 2019.

36 Wong S. Grip strength reference values for Canadians aged 6 to 79: Canadian Health Measures Survey, 2007 to 2013. *Health Reports 2016*;27(10):3–10.

37 Hoffmann MD, Colley RC, Doyon CY, Wong SL, Tomkinson GR, Lang JJ. Normative-referenced percentile values for physical fitness among Canadians. *Health Rep 2019*;30(10):14–22. doi: 10.25318/82-003-x201901000002-eng.

38 Statistics Canada. *Canadian Health Measures Survey (CHMS) Data User Guide: Cycle 4.* Ottawa (ON): Statistics Canada; 2020. Available by request: https://www.statcan.gc.ca/en/survey/household/5071.

39 Statistics Canada. *Canadian Health Measures Survey (CHMS) Data User Guide: Cycle 6.* Ottawa (ON): Statistics Canada; 2021. Available by request: https://www.statcan.gc.ca/en/survey/household/5071.

40 Mayhew AJ, So HY, Ma J, Beauchamp MK, Griffith LE, Kuspinar A, Lang JJ, Raina P. Normative values for grip strength, gait speed, timed up and go, single leg balance,

and chair rise derived from the Canadian Longitudinal Study on Ageing. *Age Ageing 2023*;52(4):afad054. doi: 10.1093/ageing/afad054.

41 Gómez-Campos R, Espinoza RV, de Arruda M, Vaz Ronque ER, Urra-Albornoz C, Minango JC, Alvear-Vasquez F, de la Torre Choque C, Correia de Campos LFC, Torres JS, Cossio-Bolaños M. Relationship between age and handgrip strength: Proposal of reference values from infancy to senescence. *Front Public Health* 2023;10:1072684. doi: 10.3389/fpubh.2022.1072684.

42 Leal Cárcamoa H, San-Martín Correa M, Martínez-Huenchullán S, Barría RM. Fuerza prensil en adultos Chilenos sanos de 20 a 69 anos: Un estudio transversal (Grip strength in Chilean healthy adults from 20 to 69 years: A cross-sectional study). *Fisioterapia 2021*:43(3);136–142. doi: 10.1016/j.ft.2020.10.003.

43 Zhao Y, Strauss J, Yang G. China Health and Retirement Longitudinal Study (2013, wave 2). Peking University Open Research Data Platform, V1; 2015. Available at: https://doi.org/10.18170/DVN/AQIU6C. [accessed 22.05.2023].

44 Zhao Y, Hu Y, Smith JP, Strauss J, Yang G. Cohort profile: the China Health and Retirement Longitudinal Study (CHARLS). *Int J Epidemiol 2014*;43(1):61–68. doi: 10.1093/ije/dys203.

45 Tian Y, Jiang C, Wang M, Cai R, Zhang Y, He Z, Wang H, Wu D, Wang F, Liu X, He Z, An P, Wang M, Tang Q, Yang Y, Zhao J, Lv S, Zhou W, Yu B, Lan J, Yang X, Zhang L, Tian H, Gu Z, Song Y, Huang T, McNaughton LR. BMI, leisure-time physical activity, and physical fitness in adults in China: Results from a series of national surveys, 2000–14. *Lancet Diabetes Endocrinol 2016*;4(6):487–497. doi: 10.1016/S2213-8587(16)00081-4.

46 Kowal P, Chatterji S, Naidoo N, Biritwum R, Fan W, Lopez Ridaura R, Maximova T, Arokiasamy P, Phaswana-Mafuya N, Williams S, Snodgrass JJ, Minicuci N, D'Este C, Peltzer K, Ties Boerma J; SAGE Collaborators. Data resource profile: The World Health Organization Study on global AGEing and adult health (SAGE). *Int J Epidemiol* 2012;41(6):1639–1649. doi: 10.1093/ije/dys210.

47 Shanghai Administration of Sports. *Report on 2020 National Physical Fitness Surveillance of Shanghai.* Available from: http://tyj.sh.gov.cn/zhxx1/20221221/b81d496efa124b1fb860398e1c7d5d63.html. [accessed 21.12.2022].

48 Ramírez-Vélez R, Rincón-Pabón D, Correa-Bautista JE, García-Hermoso A, Izquierdo M. Handgrip strength: Normative reference values in males and females aged 6–64

years old in a Colombian population. *Clin Nutr ESPEN 2021*;44:379–386. doi: 10.1016/j.clnesp.2021.05.009.

49 Ramírez-Vélez R, Correa-Bautista JE, García-Hermoso A, Cano CA, Izquierdo M. Reference values for handgrip strength and their association with intrinsic capacity domains among older adults. *J Cachexia Sarcopenia Muscle 2019*;10(2):278–286. doi: 10.1002/jcsm.12373.

50 Peasey A, Bobak M, Kubinova R, Malyutina S, Pajak A, Tamosiunas A, Pikhart H, Nicholson A, Marmot M. Determinants of cardiovascular disease and other non-communicable diseases in Central and Eastern Europe: Rationale and design of the HAPIEE study. *BMC Public Health 2006*;6:255. doi: 10.1186/1471-2458-6-255.

51 Frederiksen H, Hjelmborg J, Mortensen J, McGue M, Vaupel JW, Christensen K. Age trajectories of grip strength: Cross-sectional and longitudinal data among 8,342 Danes aged 46 to 102. *Ann Epidemiol* 2006;16(7):554–562. doi: 10.1016/j.annepidem.2005.10.006.

52 Rasmussen SH, Andersen-Ranberg K, Thinggaard M, Jeune B, Skytthe A, Christiansen L, Vaupel JW, McGue M, Christensen K. Cohort Profile: The 1895, 1905, 1910 and 1915 Danish Birth Cohort Studies—Secular trends in the health and functioning of the very old. *Int J Epidemiol 2017*;46(6):1746–1746j. doi: 10.1093/ije/dyx053.

53 Vestergaard S, Andersen-Ranberg K, Skytthe A, Christensen K, Robine JM, Jeune B. Health and function assessments in two adjacent Danish birth cohorts of centenarians: Impact of design and methodology. *Eur J Ageing 2015*;13(1):15–23. doi: 10.1007/s10433-015-0354-z.

54 Pedersen DA, Larsen LA, Nygaard M, Mengel-From J, McGue M, Dalgård C, Hvidberg L, Hjelmborg J, Skytthe A, Holm NV, Kyvik KO, Christensen K. The Danish Twin Registry: An updated overview. *Twin Res Hum Genet 2019*;22(6):499–507. doi: 10.1017/thg.2019.72.

55 Cournil A, Jeune B, Skytthe A, Gampe J, Passarino G, Robine JM. Handgrip strength: Indications of paternal inheritance in three European regions. *J Gerontol A Biol Sci Med Sci* 2010;65(10):1101–1106. doi: 10.1093/gerona/glq098.

56 Aadahl M, Beyer N, Linneberg A, Thuesen BH, Jørgensen T. Grip strength and lower limb extension power in 19–72-year-old Danish men and women: The Health2006 study. *BMJ Open 2011*;1(2):e000192. doi: 10.1136/bmjopen-2011-000192.

57 Suetta C, Haddock B, Alcazar J, Noerst T, Hansen OM, Ludvig H, Kamper RS, Schnohr P, Prescott E, Andersen LL, Frandsen U, Aagaard P, Bülow J, Hovind P,

Simonsen L. The Copenhagen Sarcopenia Study: Lean mass, strength, power, and physical function in a Danish cohort aged 20–93 years. *J Cachexia Sarcopenia Muscle 2019*;10(6):1316–29. doi: 10.1002/jcsm.12477.

58 Hogrel JY. Grip strength measured by high precision dynamometry in healthy subjects from 5 to 80 years. *BMC Musculoskelet Disord 2015*;16:139. doi: 10.1186/s12891-015-0612-4.

59 Kemmler W, Teschler M, Goisser S, Bebenek M, von Stengel S, Bollheimer LC, Sieber CC, Freiberger E. Prevalence of sarcopenia in Germany and the corresponding effect of osteoarthritis in females 70 years and older living in the community: Results of the FORMoSA study. *Clin Interv Aging 2015*;10:1565–1573. doi: 10.2147/CIA.S89585.

60 Huemer MT, Kluttig A, Fischer B, Ahrens W, Castell S, Ebert N, Gastell S, Jöckel KH, Kaaks R, Karch A, Keil T, Kemmling Y, Krist L, Leitzmann M, Lieb W, Meinke-Franze C, Michels KB, Mikolajczyk R, Velásquez IM, Pischon T, Schipf S, Schmidt B, Schöttker B, Schulze MB, Stocker H, Teismann H, Wirkner K, Drey M, Peters A, Thorand B. Grip strength values and cut-off points based on over 200,000 adults of the German National Cohort—A comparison to the EWGSOP2 cut-off points. *Age Ageing 2023*;52(1):afac324. doi: 10.1093/ageing/afac324.

61 Albrecht BM, Stalling I, Bammann K. Sex- and age-specific normative values for handgrip strength and components of the Senior Fitness Test in community-dwelling older adults aged 65–75 years in Germany: Results from the OUTDOOR ACTIVE study. *BMC Geriatr 2021*;21(1):273. doi: 10.1186/s12877-021-02188-9.

62 Schilling R, Schmidt SCE, Fiedler J, Woll A. Associations between physical activity, physical fitness, and body composition in adults living in Germany: A cross-sectional study. *PLoS One 2023*;18(10):e0293555. doi: 10.1371/journal.pone.0293555.

63 Steiber N. Strong or weak handgrip? Normative reference values for the German population across the life course stratified by sex, age, and body height. *PLoS One 2016*;11(10):e0163917. doi: 10.1371/journal.pone.0163917.

64 Goebel J, Grabka MM, Liebig S, Kroh M, Richter D, Schröder C, Schupp J. The German Socio-Economic Panel (SOEP). *J Econ Stat 2019*;239(2):345–360. doi: 10.1515/jbnst-2018-0022.

65 Liebig S, Goebel J, Grabka M, Schröder C, Zinn S, Bartels C, Franken A, Gerike M, Geschke S-C, Griese F, Kara S, König J, Krause P, Kröger H, Liebau E, Nebelin J, Petrenz M, Richter D, Schupp J, Siegers R, Steinhauer HW, Wenzig K, Zimmermann

S. *Socio-Economic Panel, data from 1984–2020.* SOEP-Core, v37, International Edition; 2022. doi: 10.5684/soep.core.v37i.

66 Bjerregaard P, Ottendahl CB, Jørgensen ME. Hand grip strength and chair stand test amongst Greenlandic Inuit: Reference values and international comparisons. *Int J Circumpolar Health 2021*;80(1):1966186. doi: 10.1080/22423982.2021.1966186.

67 Yu R, Ong S, Cheung O, Leung J, Woo J. Reference values of grip strength, prevalence of low grip strength, and factors affecting grip strength values in Chinese adults. *J Am Med Dir Assoc 2017*;18(6):551.e9–551.e16. doi: 10.1016/j.jamda.2017.03.006.

68 International Institute for Population Sciences. *Data user guide—Longitudinal Ageing Study in India (LASI) Wave 1, 2017–18, IIPS*. Mumbai; 2020.

69 Sundarakumar JS, Raviteja KV, Muniz-Terrera G, Ravindranath V. Normative data for three physical frailty parameters in an aging, rural Indian population. *Health Sci Rep 2022*;5(2):e567. doi: 10.1002/hsr2.567.

70 Mohammadian M, Choobineh A, Haghdoost A, Hasheminejad N. Normative data of grip and pinch strengths in healthy adults of Iranian population. *Iran J Public Health 2014*;43(8):1113–1122.

71 Rostamzadeh S, Saremi, M Bradtmiller B. Age, gender and side-stratified grip strength norms and related socio-demographic factors for 20–80 years Iranian healthy population: Comparison with consolidated and international norms. *Int J Ind Ergon 2020*;80:103003. doi: 10.1016/j.ergon.2020.103003.

72 Pratt J, De Vito G, Narici M, Segurado R, Dolan J, Conroy J, Boreham C. Grip strength performance from 9431 participants of the GenoFit study: Normative data and associated factors. *Geroscience 2021*;43(5):2533–2546. doi: 10.1007/s11357-021-00410-5.

73 Kenny RA, Coen RF, Frewen J, Donoghue OA, Cronin H, Savva GM. Normative values of cognitive and physical function in older adults: Findings from The Irish Longitudinal Study on Ageing. *J Am Geriatr Soc 2013*;61(S2):s279–290. doi: 10.1111/jgs.12195.

74 Langer D, Melchior H, Mazor-Karsenty T. Grip strength in healthy Israeli adults: Comparison to internationally reported normative data. *Work* 2022;71(3):787–794. doi: 10.3233/WOR-205330.

75 Landi F, Calvani R, Martone AM, Salini S, Zazzara MB, Candeloro M, Coelho-Junior HJ, Tosato M, Picca A, Marzetti E. Normative values of muscle strength across ages in

a 'real world' population: Results from the longevity check-up 7+ project. *J Cachexia Sarcopenia Muscle 2020*;11(6):1562–1569. doi: 10.1002/jcsm.12610.

76 Tomkinson GR, Kidokoro T, Dufner T, Noi S, Fitzgerald JS, McGrath RP. Temporal trends in handgrip strength for older Japanese adults between 1998 and 2017. *Age Ageing 2020*;49(4):634–639. doi: 10.1093/ageing/afaa021.

77 Ministry of Education, Culture, Sports, Science and Technology. R*eport book on the survey of physical fitness and athletic ability.* Tokyo: Ministry of Education, Culture, Sports, Science and Technology; 2001.

78 Ministry of Education, Culture, Sports, Science and Technology. *Report book on the survey of physical fitness and athletic ability.* Tokyo: Ministry of Education, Culture, Sports, Science and Technology; 2002.

79 Ministry of Education, Culture, Sports, Science and Technology. *Report book on the survey of physical fitness and athletic ability.* Tokyo: Ministry of Education, Culture, Sports, Science and Technology; 2003.

80 Ministry of Education, Culture, Sports, Science and Technology. *Report book on the survey of physical fitness and athletic ability.* Tokyo: Ministry of Education, Culture, Sports, Science and Technology; 2004.

81 Ministry of Education, Culture, Sports, Science and Technology. *Report book on the survey of physical fitness and athletic ability.* Tokyo: Ministry of Education, Culture, Sports, Science and Technology; 2005.

82 Ministry of Education, Culture, Sports, Science and Technology. *Report book on the survey of physical fitness and athletic ability.* Tokyo: Ministry of Education, Culture, Sports, Science and Technology; 2006.

83 Ministry of Education, Culture, Sports, Science and Technology. *Report book on the survey of physical fitness and athletic ability.* Tokyo: Ministry of Education, Culture, Sports, Science and Technology; 2007.

84 Ministry of Education, Culture, Sports, Science and Technology. *Report book on the survey of physical fitness and athletic ability.* Tokyo: Ministry of Education, Culture, Sports, Science and Technology; 2008.

85 Ministry of Education, Culture, Sports, Science and Technology. *Report book on the survey of physical fitness and athletic ability.* Tokyo: Ministry of Education, Culture, Sports, Science and Technology; 2009.

86 Ministry of Education, Culture, Sports, Science and Technology. *Report book on the survey of physical fitness and athletic ability.* Tokyo: Ministry of Education, Culture, Sports, Science and Technology; 2010.

87 Ministry of Education, Culture, Sports, Science and Technology. *Report book on the survey of physical fitness and athletic ability.* Tokyo: Ministry of Education, Culture, Sports, Science and Technology; 2011.

88 Ministry of Education, Culture, Sports, Science and Technology. *Report book on the survey of physical fitness and athletic ability.* Tokyo: Ministry of Education, Culture, Sports, Science and Technology; 2012.

89 Ministry of Education, Culture, Sports, Science and Technology. *Report book on the survey of physical fitness and athletic ability.* Tokyo: Ministry of Education, Culture, Sports, Science and Technology; 2013.

90 Ministry of Education, Culture, Sports, Science and Technology. *Report book on the survey of physical fitness and athletic ability.* Tokyo: Ministry of Education, Culture, Sports, Science and Technology; 2014.

91 Ministry of Education, Culture, Sports, Science and Technology. *Report book on the survey of physical fitness and athletic ability.* Tokyo: Ministry of Education, Culture, Sports, Science and Technology; 2015.

92 Ministry of Education, Culture, Sports, Science and Technology. *Report book on the survey of physical fitness and athletic ability.* Tokyo: Ministry of Education, Culture, Sports, Science and Technology; 2016.

93 Ministry of Education, Culture, Sports, Science and Technology. *Report book on the survey of physical fitness and athletic ability.* Tokyo: Ministry of Education, Culture, Sports, Science and Technology; 2017.

94 Ministry of Education, Culture, Sports, Science and Technology. *Report book on the survey of physical fitness and athletic ability.* Tokyo: Ministry of Education, Culture, Sports, Science and Technology; 2018.

95 Ministry of Education, Culture, Sports, Science and Technology. *Report book on the survey of physical fitness and athletic ability.* Tokyo: Ministry of Education, Culture, Sports, Science and Technology; 2019.

96 Ministry of Education, Culture, Sports, Science and Technology. *Report book on the survey of physical fitness and athletic ability.* Tokyo: Ministry of Education, Culture, Sports, Science and Technology; 2020.

97 Kozakai R, Nishita Y, Otsuka R, Ando F, Shimokata H. Age-related changes in physical fitness among community-living middle-aged and older Japanese: A 12-year longitudinal study. *Res Q Exerc Sport 2020*;91(4):662–675. doi: 10.1080/02701367.2019.1697418.

98 Yoshimura N, Oka H, Muraki S, Akune T, Hirabayashi N, Matsuda S, Nojiri T, Hatanaka K, Ishimoto Y, Nagata K, Yoshida M, Tokimura F, Kawaguchi H, Nakamura K. Reference values for hand grip strength, muscle mass, walking time, and one-leg standing time as indices for locomotive syndrome and associated disability: The second survey of the ROAD study. *J Orthop Sci* 2011;16(6):768–777. doi: 10.1007/s00776-011-0160-1.

99 Matsumoto K, Gondo Y, Masui Y, Yasumoto S, Yoshida Y, Ikebe K, Arai Y, Kabayama M, Kamide K, Akasaka H, Ishizaki T. Physical performance reference values for Japanese oldest old: A SONIC study. *BMC Geriatr 2022*;22(1):748. doi: 10.1186/s12877-022-03299-7.

100 Taniguchi Y, Fujiwara Y, Murayama H, Yokota I, Matsuo E, Seino S, Nofuji Y, Nishi M, Matsuyama Y, Shinkai S. Prospective study of trajectories of physical performance and mortality among community-dwelling older Japanese. *J Gerontol A Biol Sci Med Sci 2016*;71(11):1492–1499. doi: 10.1093/gerona/glw029.

101 Seino S, Shinkai S, Fujiwara Y, Obuchi S, Yoshida H, Hirano H, Kim HK, Ishizaki T, Takahashi R, TMIG-LISA Research Group. Reference values and age and sex differences in physical performance measures for community-dwelling older Japanese: A pooled analysis of six cohort studies. *PLoS One 2014*;9(6):e99487. doi: 10.1371/journal.pone.0099487.

102 Macao Sport Development Board, Macao SAR. *Report of the study of health and fitness on adults in Macao SAR in 2001.* Macao: Macao Sport Development Board, Macao SAR; 2003. Available at: https://www.sport.gov.mo/en/cmd/downloadflyer. [accessed 15.12.2023] ISBN 99937-682-2-7.

103 Macao Sport Development Board, Macao SAR. *2005 Physical Fitness Report of Macao SAR Citizens.* Macao: Macao Sport Development Board, Macao SAR, 2006. Available at: https://www.sport.gov.mo/en/cmd/downloadflyer. [accessed 15.12.2023] ISBN 99937-682-9-4.

104 Macao Sport Development Board, Macao SAR. *2010 Physical Fitness Report of Macao SAR Citizens.* Macao: Macao Sport Development Board, Macao SAR, 2011.

Available at: https://www.sport.gov.mo/en/cmd/downloadflyer. [accessed 15.12.2023] ISBN 978-99937-892-9-1.

105 Sports Bureau, Macao SAR. *2015 Physical Fitness Report of Macao SAR Residents.* Macao: Sports Bureau, Macao SAR, 2017. Available at: https://www.sport.gov.mo/en/cmd/downloadflyer. [accessed 15.12.2023] ISBN 978-99981-897-3-7.

106 Sports Bureau, Macao SAR. *2020 Physical Fitness Report of Macao SAR Residents.* Macao: Sports Bureau, Macao SAR; 2023. Available at: https://www.sport.gov.mo/en/cmd/downloadflyer. [accessed 15.12.2023] ISBN 978-99981-897-3-7.

107 Lam NW, Goh HT, Kamaruzzaman SB, Chin AV, Poi PJH, Tan MP. Normative data for hand grip strength and key pinch strength, stratified by age and gender for a multiethnic Asian population. *Singapore Med J 2016*;57(10):578–584. doi: 10.11622/smedj.2015164.

108 Alex D, Khor HM, Chin AV, Hairi NN, Cumming RG, Othman S, Khoo S, Kamaruzzaman SB, Tan MP. Factors associated with falls among urban-dwellers aged 55 years and over in the Malaysian Elders Longitudinal Research (MELoR) study. *Front Public Health 2020*;8:506238. doi: 10.3389/fpubh.2020.506238.

109 Shah SA, Safian N, Mohammad Z, Nurumal SR, Ibadullah WAHW, Mansor J, Ahmad S, Hassan MR, Shobugawa Y. Factors associated with handgrip strength among older adults in Malaysia. *J Multidiscip Healthc 2022*;15:1023–1034. doi: 10.2147/JMDH.S363421.

110 Enríquez-Reyna MC, Bautista DC, Orocio RN. Nivel de actividad física, masa y fuerza muscular de mujeres mayores de la comunidad: Diferencias porgrupo etario (Physical activity level, muscle mass and strength of community elderly women: Differences by age group). *Retos* 2019;35:121–125. doi: 10.47197/retos.v0i35.59956.

111 Malina RM, Peña Reyes ME, Gonzalez Alvarez C, Little BB. Age and secular effects on muscular strength of indigenous rural adults in Oaxaca, Southern Mexico: 1978–2000. *Ann Hum Biol* 2011;38(2):175–187. doi: 10.3109/03014460.2010.504196.

112 Núñez-Othón G, Romero-Pérez EM, Camberos NA, Horta-Gim MA, Tánori-Tapia JM, de Paz JA. Functional capacity of noninstitutionalized older adults from northwest Mexico: reference values. *Healthcare (Basel)* 2023;11(12):1733. doi: 10.3390/healthcare11121733.

113 Rodríguez-García WD, García-Castañeda L, Orea-Tejeda A, Mendoza-Núñez V, González-Islas DG, Santillán-Díaz C, Castillo-Martínez L. Handgrip strength: Reference values and its relationship with bioimpedance and anthropometric variables. *Clinical Nutrition ESPEN 2017*;19:54–8. doi: 10.1016/j.clnesp.2017.01.010.

114 Rodríguez-García WD, García-Castañeda L, Vaquero-Barboas N, Mendoza-Núñez V, Orea-Tejeda A, Perkisas S, Vandewoude M, Castillo-Martínez L. Prevalence of dynapenia and presarcopenia related to aging in adult community-dwelling Mexicans using two different cut-off points. *Eur Geriatr Med 2018*;9(2):219–225. doi: 10.1007/s41999-018-0032-8.

115 Bimali I, Opsana R, Jeebika S. Normative reference values on handgrip strength among healthy adults of Dhulikhel, Nepal: A cross-sectional study. *J Family Med Prim Care 2020*;9(1):310–314. doi: 10.4103/jfmpc.jfmpc_785_19.

116 Kim JK, Son WI, Sim YJ, Lee JS, Saud KO. The study of health-related fitness normative scores for Nepalese older adults. *Int J Environ Res Public Health 2020*;17(8):2723. doi: 10.3390/ijerph17082723.

117 Tveter AT, Dagfinrud H, Moseng T, Holm I. Health-related physical fitness measures: Reference values and reference equations for use in clinical practice. *Arch Phys Med Rehabil 2014*;95(7):1366–1373. doi: 10.1016/j.apmr.2014.02.016.

118 Kjær IGH, Torstveit MK, Kolle E, Hansen BH, Anderssen SA. Normative values for musculoskeletal- and neuromotor fitness in apparently healthy Norwegian adults and the association with obesity: A cross-sectional study. *BMC Sports Sci Med Rehabil 2016*;8:37. doi: 10.1186/s13102-016-0059-4.

119 Nilsen T, Hermann M, Eriksen CS, Dagfinrud H, Mowinckel P, Kjeken I. Grip force and pinch grip in an adult population: Reference values and factors associated with grip force. *Scand J Occup Ther* 2012;19(3):288–296. doi: 10.3109/11038128.2011.553687.

120 Svinøy OE, Hilde G, Bergland A, Strand BH. Reference values for Jamar+ digital dynamometer hand grip strength in healthy adults and in adults with non-communicable diseases or osteoarthritis: the Norwegian Tromsø study 2015–2016. *Eur J Ageing 2023*;20(1):44. doi: 10.1007/s10433-023-00791-w.

121 Afable SB, Cruz G, Saito Y, Malhotra R. Normative values of hand grip strength of older Filipinos aged 60 to 85 years. *Aging Health Res 2022*;2(4):100108. doi: 10.1016/j.ahr.2022.100108.

122 Wiśniowska-Szurlej A, Ćwirlej-Sozańska A, Kilian J, Wołoszyn N, Sozański B, Wilmowska-Pietruszyńska A. Reference values and factors associated with hand grip

strength among older adults living in southeastern Poland. *Sci Rep 2021*;11(1):9950. doi: 10.1038/s41598-021-89408-9.

123 Mendes J, Amaral TF, Borges N, Santos A, Padrão P, Moreira P, Afonso C, Negrão R. Handgrip strength values of Portuguese older adults: A population based study. *BMC Geriatr 2017*;17(1):191. doi: 10.1186/s12877-017-0590-5.

124 Kim M, Won CW, Kim M. Muscular grip strength normative values for a Korean population from the Korea National Health and Nutrition Examination Survey, 2014–2015. *PLoS One 2018*;13(8):e0201275. doi: 10.1371/journal.pone.0201275.

125 Kweon S, Kim Y, Jang MJ, Kim Y, Kim K, Choi S, Chun C, Khang YH, Oh K. Data resource profile: The Korea National Health and Nutrition Examination Survey (KNHANES). *Int J Epidemiol* 2014;43(1):69–77. doi: 10.1093/ije/dyt228.

126 Ministry of Culture, Sports and Tourism. *National survey of physical fitness.* Seoul: Korean Institute of Sports Science; 2009.

127 Ministry of Culture, Sports and Tourism. *National survey of physical fitness.* Seoul: Korean Institute of Sports Science; 2011.

128 Ministry of Culture, Sports and Tourism. *National survey of physical fitness.* Seoul: Korean Institute of Sports Science; 2013.

129 Ministry of Culture, Sports and Tourism. *National survey of physical fitness.* Seoul: Korean Institute of Sports Science; 2015.

130 Ministry of Culture, Sports and Tourism. *National survey of physical fitness.* Seoul: Korean Institute of Sports Science; 2017.

131 Cooper R, Shkolnikov VM, Kudryavtsev AV, Malyutina S, Ryabikov A, Arnesdatter Hopstock L, Johansson J, Cook S, Leon DA, Strand BH. Between-study differences in grip strength: A comparison of Norwegian and Russian adults aged 40–69 years. *J Cachexia Sarcopenia Muscle 2021*;12(6):2091–2100. doi: 10.1002/jcsm.12816.

132 Oksuzyan A, Demakakos P, Shkolnikova M, Thinggaard M, Vaupel JW, Christensen K, Shkolnikov VM. Handgrip strength and its prognostic value for mortality in Moscow, Denmark, and England. *PLoS One 2017*;12(9):e0182684. doi: 10.1371/journal.pone.0182684.

133 Turusheva A, Frolova E, Degryse J-M. Age-related normative values for handgrip strength and grip strength's usefulness as a predictor of mortality and both cognitive and physical decline in older adults in northwest Russia. *J Musculoskelet Neuronal Interact 2017*;17(1):417–432.

134 Alqahtani B, Alenazi A, Alshehri M, Alqahtani M, Elnaggar R. Reference values and associated factors of hand grip strength in elderly Saudi population: A cross-sectional study. *BMC Geriatr 2019*;19(1):271. doi: 10.1186/s12877-019-1288-7.

135 Alrashdan A, Ghaleb AM, Almobarek M. Normative static grip strength of Saudi Arabia’s population and influences of numerous factors on grip strength. *Healthcare (Basel) 2021*;9(12):1647. doi: 10.3390/healthcare9121647.

136 Malhotra R, Ang S, Allen JC, Tan NC, Østbye T, Saito Y, Chan A. Normative values of hand grip strength for elderly Singaporeans aged 60 to 89 years: A cross-sectional study. *J Am Med Dir Assoc 2016*;17(9):864.e1–7. doi: 10.1016/j.jamda.2016.06.013.

137 Ong HL, Abdin E, Chua BY, Zhang Y, Seow E, Vaingankar JA, Chong SA, Subramaniam M. Hand-grip strength among older adults in Singapore: A comparison with international norms and associative factors. *BMC Geriatr 2017*;17(1):176. doi: 10.1186/s12877-017-0565-6.

138 Lee SY, Choo PL, Pang BWJ, Lau LK, Jabbar KA, Seah WT, Chen KK, Ng TP, Wee S-L. SPPB reference values and performance in assessing sarcopenia in community-dwelling Singaporeans—Yishun study. *BMC Geriatr 2021;*21(1):213. doi: 10.1186/s12877-021-02147-4.

139 Sánchez Torralvo FJ, Porras N, Abuín Fernández J, García Torres F, Tapia MJ, Lima F, Soriguer F, Gonzalo M, Rojo Martínez G, Olveira G. Normative reference values for hand grip dynamometry in Spain. Association with lean mass. *Nutr Hosp 2018*;35(1):98–103. doi: 10.20960/nh.1052.

140 Axelsson P, Fredrikson P, Nilsson A, Andersson JK, Kärrholm J. Forearm torque and lifting strength: Normative data. *J Hand Surg Am* 2018;43(7):677.e1–677.e17. doi: 10.1016/j.jhsa.2017.12.022.

141 Ribom EL, Mellström D, Ljunggren O, Karlsson MK. Population-based reference values of handgrip strength and functional tests of muscle strength and balance in men aged 70–80 years. *Arch Gerontol Geriatr 2011*;53(2):e114–117. doi: 10.1016/j.archger.2010.07.005.

142 Lichtenstein E, Wagner J, Knaier R, Infanger D, Roth R, Hinrichs T, Schmidt-Trucksaess A, Faude O. Norm values of muscular strength across the life span in a healthy Swiss population: The COmPLETE study. *Sports Health 2023*;15(4):547–557. doi: 10.1177/19417381221116345.

143 Wearing J, Konings P, Stokes M, de Bruin ED. Handgrip strength in old and oldest old Swiss adults—A cross-sectional study. *BMC Geriatr 2018*;18(1):266. doi: 10.1186/s12877-018-0959-0.

144 Werle S, Goldhahn J, Drerup S, Simmen B, Sprott H, Herren D. Age- and gender-specific normative data of grip and pinch strength in a healthy adult Swiss population. *J Hand Surg Eur Vol* 2009;34(1):76–84. doi: 10.1177/1753193408096763.

145 Liu LK, Lee WJ, Liu CL, Chen LY, Lin MH, Peng LN, Chen LK. Age-related skeletal muscle mass loss and physical performance in Taiwan: Implications to diagnostic strategy of sarcopenia in Asia. *Geriatr Gerontol Int 2013*;13(4):964–971. doi: 10.1111/ggi.12040.

146 Dodds RM, Pakpahan E, Granic A, Davies K, Sayer AA. The recent secular trend in grip strength among older adults: Findings from the English Longitudinal Study of Ageing. *Eur Geriatr Med* 2019;10(3):395–401. doi: 10.1007/s41999-019-00174-4.

147 Banks J, Batty GD, Breedvelt J, Coughlin K, Crawford R, Marmot M, Nazroo J, Oldfield Z, Steel N, Steptoe A, Wood M, Zaninotto P. *English Longitudinal Study of Ageing: Waves 0–9, 1998–2019. [data collection]. 38th Edition.* UK Data Service. SN: 5050 2023. doi: 10.5255/UKDA-SN-5050-25.

148 Keevil VL, Hayat S, Dalzell N, Moore S, Bhaniani A, Luben R, Wareham NJ, Khaw KT. The physical capability of community-based men and women from a British cohort: The European Prospective Investigation into Cancer (EPIC)-Norfolk study. *BMC Geriatr 2013*;13:93. doi: 10.1186/1471-2318-13-93.

149 Syddall HE, Aihie Sayer A, Dennison EM, Martin HJ, Barker DJP, Cooper C. Cohort profile: The Hertfordshire Cohort Study. *Int J Epidemiol 2005*;34(6):1234–1242. doi: 10.1093/ije/dyi127.

150 Bardo A, Kivell TL, Town K, Donati G, Ballieux H, Stamate C, Edginton T, Forrester GS. Get a grip: Variation in human hand grip strength and implications for human evolution. *Symmetry* 2021;13(7):1142. doi: 10.3390/sym13071142.

151 Benzeval M, Der G, Ellaway A, Hunt K, Sweeting H, West P, Macintyre S. Cohort profile: west of Scotland Twenty-07 study: Health in the community. *Int J Epidemiol 2009*;38(5):1215–23. doi: 10.1093/ije/dyn213.

152 University of Essex, Institute for Social and Economic Research. *Understanding Society: Waves 2–3 Nurse Health Assessment, 2010–2012. [data collection]. 5th Edition.* UK Data Service. SN: 7251 2023. doi: 10.5255/UKDA-SN-7251-5.

153 Wozny R, Pratt AL, Pereira C. A study of grip strength among 20–49-year-old British adults and comparison to existing norms. *Hand Ther 2015*;20(4):115–123. doi:10.1177/1758998315599792.

154 Health and Retirement Study. *HRS Data Book.* Available at: https://hrs.isr.umich.edu/about/data-book?_ga=2.177450149.1489958521.1509473800-353572931.1501594459. [accessed 01.01.2023].

155 Health and Retirement Study. *HRS Data Products.* Available at: https://hrs.isr.umich.edu/data-products. [accessed 01.01.2023].

156 Warden SJ, Liu Z, Moe SM. Sex- and age-specific centile curves and downloadable calculator for clinical muscle strength tests to identify probable sarcopenia. *Phys Ther 2022*;102(3):pzab299. doi:10.1093/ptj/pzab299.

157 Wang Y-C, Bohannon RW, Li X, Yen S-C, Sindhu B, Kapellusch J. Summary of grip strength measurements obtained in the 2011–2012 and 2013–2014 National Health and Nutrition Examination Surveys. *J Hand Ther 2019*;32(4):489–496. doi: 10.1016/j.jht.2018.03.002.

158 Wang Y-C, Bohannon RW, Li X, Sindhu B, Kapellusch J. Hand-grip strength: Normative reference values and equations for individuals 18 to 85 years of age residing in the United States. *J Orthop Sports Phys Ther 2018*;48(9):685–693. doi: 10.2519/jospt.2018.7851.

159 Gershon R. *NIH Toolbox Norming Study;* 2016. Available at: https://doi.org/10.7910/DVN/FF4DI7, Harvard Dataverse, V4, UNF:6:bOqMnZEEG/rBz6SQyN4t2g== [fileUNF] [accessed 15.01.2023].

160 United Nations Development Programme. *The 2021/2022 Human Development Report: Uncertain times, unsettled lives Shaping our future in a transforming world.* New York: United Nations; 2022.

161 Avakov AV. *Quality of life, balance of powers, and nuclear weapons (2012): A statistical yearbook for statesmen and citizens.* New York, NY: Algora Publishing; 2012.

162 Government of Macao Special Administrative Region Statistics and Census Service. *Macao in figures.* Government of Macao Special Administrative Region Statistics and Census Service 2023. Available at: https://www.dsec.gov.mo. [accessed 05.02.2024]

163 *Human Development Index, HDI.* Available at: https://eng.stat.gov.tw/News_Content.aspx?n=4610&s=233232. [accessed 05.07.2024].
